# Effectiveness of interventions to promote social inclusion of people with disabilities in high-income countries: a systematic review of quantitative evaluation studies

**DOI:** 10.1136/bmjopen-2026-117213

**Published:** 2026-06-16

**Authors:** Manon Schroeder, Germain Weber, Marc Suhrcke

**Affiliations:** 1 Luxembourg Institute of Socio-Economic Research, Esch-sur-Alzette, Luxembourg; 2 Department of Social Sciences, University of Luxembourg, Esch-sur-Alzette, Luxembourg; 3 Department of Clinical and Health Psychology, University of Vienna Faculty of Psychology, Vienna, Austria; 4 Centre for Health Economics, University of York, York, England, UK

**Keywords:** Disabled Persons, Systematic Review, Community Participation

## Abstract

**Abstract:**

**Objectives:**

Despite policy commitments to social inclusion for people with disabilities, the effectiveness of interventions remains unclear, particularly in high-income countries. This systematic review synthesises evidence and examines variation by population, setting and intervention.

**Design:**

Systematic review.

**Setting:**

All settings in which social inclusion interventions might be implemented in high-income countries.

**Participants:**

Children and adults with disabilities (0–65 years) living in high-income countries.

**Interventions:**

Promoting social inclusion for people with disabilities.

**Eligibility criteria:**

Studies were eligible if they were peer-reviewed, quantitative evaluations of interventions aimed at improving social inclusion among people with disabilities. The studies had to have been conducted in high-income countries and report pre/post or between-group outcomes. We included randomised controlled trials, non-randomised designs and before–after designs. We excluded qualitative studies, studies with fewer than 20 participants and research conducted outside high-income settings.

**Data sources:**

We searched seven databases (Web of Science, Scopus, PubMed, MEDLINE, CINAHL Complete, APA PsycInfo and Eric), including studies published up to 20 March 2026.

**Risk of bias:**

Risk of bias was assessed using ROB2 (revised Cochrane risk-of-bias tool for randomised trials) and ROBINS-I (risk of bias for non-randomised studies – of interventions) tools.

**Synthesis of results:**

Results were synthesised narratively and structured by intervention type and outcome domain.

**Primary and secondary outcome measures:**

Outcome measures related to social inclusion, including social skills, broad social participation and interpersonal and community relationships, classified within an ecological framework aligned with the WHO CBR (community-based rehabilitation) domains.

**Results:**

50 studies met the inclusion criteria, covering a total of 10 904 participants. 20 studies (40%) reported significant positive effects, 14 (28%) no effect and 16 (32%) mixed outcomes. Positive effects were most frequently observed for interventions targeting social and communication skills, whereas null effects were more common for broader community participation outcomes. Risk of bias was low in 8 studies (16%), moderate in 21 (42%) and high in 21 (42%), with no significant association between risk of bias and reported effectiveness. Evidence gaps included limited representation of diverse disability groups, few structural interventions and sparse outcomes on identity, rights and justice.

**Conclusion:**

Some promising practices exist (notably family-centred and interpersonal), but the evidence base is fragmented and weighted towards individual change. Future research and policy should prioritise holistic, multilevel strategies that address relational and structural barriers alongside individual supports.

**Trial registration:**

The systematic review was preregistered on the Open Science Framework (https://osf.io/9wfqyhttps://osf.io/9wfqy).

**STRENGTHS AND LIMITATIONS OF THIS STUDY**

- A comprehensive systematic search was conducted, and the quality of the studies was assessed using validated risk-of-bias tools (ROB2 (revised Cochrane risk-of-bias tool for randomised trials) and ROBINS-I (risk of bias for non-randomised studies – of interventions)).
- Including non-randomised studies reflected the field’s methodological reality and enabled a more comprehensive synthesis of the available evidence.
- The review was limited to quantitative studies from high-income countries with sample sizes of over 20, which may have excluded small-scale or qualitative evidence.
- Many non-randomised studies demonstrated low or moderate methodological quality, primarily due to inadequate control of confounding factors, which limited comparability and the strength of inferences.

## Introduction

 People with disabilities remain systematically excluded from full participation in society on multiple levels, as they are likely to experience exclusion from family and community activities, to be unemployed and experience impoverished living conditions.^1^,^3^ Previous community-based rehabilitation (CBR) programmes have predominantly focused on health issues and clinical rehabilitation, often overlooking the social dimensions of the lives of people with disabilities. However, the inclusion of people with disabilities into family and community life is crucial for their personal development, shaping identity, self-esteem and quality of life.^4^ The International Classification of Functioning, Disability and Health (ICF) underscores this, by conceptualising functioning as the dynamic interaction of health conditions with contextual factors (environmental and personal). The ICF integrates the medical and social perspectives, with participation designated as a fundamental domain.^5^

Social inclusion encompasses both the process and goal of improving participation in social, economic, cultural and political domains of society.^6^ It is not solely determined by individual characteristics but also by interpersonal processes^7^ that ensure equal access for people with disabilities.^6 8^ Simplican *et al*^3^ proposed an ecological model emphasising two interconnected domains of social inclusion: interpersonal relationships and community participation, both of which are embedded within individual, organisational, community and socio-political contexts.

Despite social inclusion having been recognised as a major stated policy aim, not least via the ratification of the United Nations (UN) Convention on the Rights of Persons with Disabilities (CRPD)^9^ by a large number of countries, progress towards achieving this goal has been inconsistent to date, even among some of the wealthiest countries,^10^,^12^ and the road ahead is still long to eliminate barriers to the inclusion of people with disabilities.^13^ Persistent barriers prevent individuals with disabilities from engaging equally in educational, occupational, civic and social domains.^14^ These barriers occur across individual, community and socio-political levels,^3^ including negative attitudes from the community,^15^,^17^ inaccessible environments such as transport and housing^18^,^20^ or personal factors, including the level of disability, gender and health.^20^ Overcoming these barriers requires enhanced opportunities for people with disabilities,^21^ better access to resources, giving them a voice and respecting their rights.^22^ Addressing these needs will require targeted interventions and research at the local and population level^2 3 23^ tailored to the personal and environmental factors of people with disabilities.^3 24^ To provide guidance, the WHO developed the CBR guidelines to support stakeholders in promoting the full participation of people with disabilities in all aspects of social life, through strategies ranging from strengthening social skills to addressing negative attitudes and driving systemic change.^4^

The effectiveness of social inclusion interventions has previously been reviewed across various target populations and intervention types, including attitudes,^25^,^27^ social,^28^ health and care,^29^ multifaceted approaches,^30^ befriending^24^ or sport participation interventions.^31 32^ Most of these reviews focused on specific disability types, for example, people with intellectual,^24^,^2733^ learning disabilities,^29^ psychosocial disabilities, autism spectrum disorder^36^ or neurodevelopmental disabilities.^37^ A meta-analysis conducted by Saran *et al*^38^ examined the effectiveness of social inclusion interventions in low- and middle-income countries. No systematic review, however, appears to exist on social inclusion interventions with a more comprehensive scope across types of disability, while accounting for the heterogeneity of the target population, and focusing on high-income countries.

This systematic review seeks to fill this gap, by assessing the effectiveness of interventions designed to promote social inclusion for people with disabilities in high-income countries. It uses a combined ecological framework, based on the model by Simplican *et al*^3^ and the WHO^4^ CBR matrix, to classify interventions and outcomes. It also explores whether effectiveness varies by intervention category, disability type, respondent type and outcome domain. In doing so, the review contributes to a clearer understanding of what works to support inclusion, for whom and in what contexts. Consistent with the ICF, we interpret participation as both a health-related outcome and a determinant of well-being, making observed inclusion effects equity-relevant.^5 14^

## Materials and methods

The reviews followed Preferred Reporting Items for Systematic Reviews and Meta-Analyses (PRISMA) guidelines and were preregistered on the Open Science Framework (OSF) (https://osf.io/9wfqy). It includes randomised and non-randomised studies evaluating the effectiveness of interventions aiming to improve social inclusion for people with disabilities in high-income countries.

### Study inclusion and exclusion criteria

Studies were included if they:

- Reported original, peer-reviewed research.
- Were conducted in high-income countries (as defined by World Bank as of 1 July 2023).
- Included participants with disabilities according to the UNCRPD definition.
- Included children (and youth) and working-age adults (0–65 years).
- Evaluated an intervention aiming to improve at least one domain of social inclusion (see *Types of interventions* for details of the interventions).
- Used quantitative designs (randomised control trials (RCT), quasi-experimental, non-RCT or single-group before-and-after studies).
- Reported measurable pre/post or between-group outcomes related to inclusion (see *Types of outcome measures* for details of the outcomes).

Studies were excluded:

- If the sample size was very small (<20 participants), to ensure a minimum level of statistical robustness, though we acknowledge this threshold is ultimately arbitrary;
- If no statistical methods were used for the outcome of interest;
- If they did not include an impact evaluation of an intervention or if the impact evaluation did not assess outcomes related to social inclusion for people with disabilities;
- If the references were not found; or
- If the study used exclusively qualitative methods.
  (see online supplemental appendix 1 for details of the inclusion/exclusion criteria).

Qualitative studies were excluded, because the review focused on quantitatively measured changes in social inclusion outcomes, in order to evaluate the effectiveness of interventions. While qualitative research provides valuable insights into participants’ lived experiences and perspectives, it does not yield the outcome measures needed to assess whether interventions produce measurable change.

The age range of 0–65 years was selected to capture social inclusion across childhood, youth and working-age adulthood. The upper limit was applied to keep the review focused on disability-related social inclusion interventions and to avoid conflating these with issues more specific to ageing, retirement and later-life care, as participation in later life is commonly examined within distinct research traditions concerned with age-related health and long-term care (eg, studies by Levasseur *et al*, Fujita *et al* and Harwood *et al*^39^,^41^). Both children and adults were included because social inclusion is a life-course construct, and the review aimed to examine whether intervention patterns and effectiveness differ across age groups. No lower age limit was applied, as social exclusion associated with disability often begins in early childhood, and many interventions aiming to improve social inclusion are implemented in educational, family and community settings. In addition, several studies in this field include both children and adults within the same intervention, such that excluding children would have led to the omission of relevant evidence.

### Types of interventions

The interventions that met the criteria for inclusion were to be designed to evaluate the promotion of social inclusion for people with disabilities. These interventions could be implemented at the individual, interpersonal, organisational, community or socio-political level.^3^ The following intervention categories were defined: relationships, marriage and family; culture and arts; recreation, leisure and sports; justice; assistive technology and rehabilitation; and policies and programmes.^42^ A brief overview of both frameworks and their respective categories is provided in table 1, while the full classification matrix is presented in online supplemental appendix 2.

**Table 1:** Summary of frameworks used to classify social inclusion interventions

Dimension 1: CBR matrix intervention domains (WHO;^4^ Saran *et al*^42^)	
Intervention domain	Key components
Personal assistance	Formal and informal personal support
Relationship, marriage and family	Social networking, community attitude improvement, communication skill training, violence prevention
Culture and arts	Access to cultural programmes, arts, drama, theatre, religious activities
Recreation, leisure and sports	Access to sports events, recreation and leisure activities
Justice	Accessibility of and access to the legal system
Assistive technology and rehabilitation	Assistive technology, rehabilitation, medical care
Policies and programmes	International legislation, social inclusion policies

Dimension 2: ecological levels of intervention (Simplican *et al*^3^)	
Ecological level	Definition
Individual	Personal-level factors
Interpersonal	Attitudes of social network members; relationships with staff, family and friends
Organisational	Group home culture, staff attitudes and training, organisational cultures of community institutions (eg, schools, employment centres, law enforcement), access to communication services
Community	Availability and access to appropriate services, transportation, online communities, community attitudes, culture, geography
Socio-political	Laws, legal enforcement, market forces, state perspectives, histories of service delivery, legislative cutbacks

Each intervention identified in this review was classified using both dimensions simultaneously, as illustrated in the full matrix of social inclusion outcomes (online supplemental appendix 2)*.*

CBR, community-based rehabilitation.

To classify the interventions of the studies, a matrix was developed, combining the social inclusion intervention categories related to the social pillar of the CBR matrix,^4^ complemented by Saran *et al* (2021) and Simplican *et al*’s (2015) ecological levels (see online supplemental appendix 2). Due to the multilevel-approach of some interventions, they were classified into multiple categories.

### Types of outcome measures

The outcomes of interest in this review were to focus on changes in various dimensions of social inclusion, with an emphasis on the social pillar of the CBR component matrix: social (social identity, personal assistance), skills for social inclusion (social and communication skills, social behaviour), broad-based social inclusion and participation measures (community inclusion, community participation, access to justice) and relationships (interpersonal and family relationship, peer and community relationship, violence and abuse). These outcomes were at the individual, interpersonal, organisational, community or socio-political level.^3^ Although we did not require clinical endpoints, the inclusion outcomes we focused on (eg, participation, social relationships, community engagement) are established determinants of health and well-being, and thus equity-relevant. A brief overview of both frameworks and their respective outcome categories is provided in table 2, while the full classification matrix is presented in online supplemental appendix 3.

**Table 2:** Summary of frameworks used to classify social inclusion outcomes

Dimension 1: CBR matrix outcome domains (WHO^4^; Saran *et al*^42^)		
Outcome domain	Subdomain	Key components
Social	Social identity	Self-concept, sense of belonging to a social group
	Personal assistance	Individual support plans, family support access
Skills for social inclusion	Social and communication skills	Verbal/nonverbal behaviour, communication aids, speech and reading devices
	Social behaviour	Behaviours that connect individuals socially
Broad-based social inclusion and participation	Social inclusion	Time spent outside home, travelling
	Community integration	Optimising personal, social and vocational competencies to live in the community
	Community participation	Leisure, civic, productive, religious and cultural activities in community
	Access to justice	Access to or interaction with the legal system
Relationships	Interpersonal and family relationships	Sense of belonging, household participation, positive family attitudes
	Peer and community relationships	Community acknowledgement of meaningful relationships, marriage, parenthood
	Violence and abuse	Protection from violence, stakeholder collaboration

Dimension 2: ecological levels of outcomes (Simplican *et al*^3^)	
Ecological level	Definition
Individual	Increased happiness, improved self-esteem, sense of belonging
Interpersonal	Respect and trust between people, increased social capital
Organisational	Changes in organisational culture toward greater inclusion
Community	Availability of and access to appropriate services and transportation, positive community attitudes
Socio-political	Political change, new or revised laws

Each outcome was classified using both dimensions simultaneously as illustrated in the full matrix of social inclusion interventions (online supplemental appendix 3).

CBR, community-based rehabilitation.

The social inclusion outcomes of the included studies were classified using a matrix combining the social inclusion outcomes categories related to Simplican *et al*’s^3^ ecological levels and to the social pillar of the CBR matrix complemented by Saran *et al*’s^42^ (see online supplemental appendix 3). Due to the multi-approach nature of the outcomes, it is possible for one outcome to be classified into several categories of the matrix.

### Search strategy

A comprehensive search was conducted across seven multidisciplinary databases: Web of Science (Clarivate), Scopus (Elsevier), PubMed (NCBI), MEDLINE (Ovid), CINAHL Complete (EBSCOhost), APA PsycInfo (Ovid) and Eric (EBSCOhost), between 8 April 2024 and 20 March 2026. The search was updated on 20 March 2026 to identify any additional studies published since the initial search.

The search combined terms related to disability, social inclusion, high-income countries (following the World Bank classification) and intervention effectiveness. Keywords related to disability and high-income countries were informed by the systematic review by Saran *et al*^38 42^ and adapted for the purposes of the present study. The search strategy was developed by the first author and reviewed by the coauthors prior to implementation. The full search strategy for all databases is provided in online supplemental appendix 4. No limits or filters were applied in terms of language or publication date. The search reporting follows the PRISMA-S, a PRISMA search reporting extension.^43^

### Study quality assessment

The risk of bias was assessed using the revised Cochrane risk-of-bias tool for randomised trials (ROB2)^44^ and the risk of bias for non-randomised studies – of interventions (ROBINS-I) assessment tool for non-randomised studies.^45^ This approach was selected as it permits a comprehensive evaluation of bias across diverse study designs.^46^ The ROB2 tool classifies risk of bias across five domains: the randomisation process, deviations from intended interventions, missing outcome data, outcome measurement and selection of reported results. Each domain is rated as *low, some concern* or *high*. The ROBINS-I tool evaluates risk of bias across seven domains: domains confounding, participant selection, intervention classification, deviations from intended interventions, missing outcome data, outcome measurement and selection of reported results, with judgments categorised as *low, moderate, serious* or *critical* risk of bias.

Given the range of study designs in our review, we added further considerations for single-group before–after studies when applying the ROBINS-I: in Stage 2 (bias in selection of participants) and Stage 3 (bias in classification of interventions), we introduced a *Not Applicable* category for single-group studies to avoid penalising them for inherent design characteristics.

The risk-of-bias tools were piloted on a random sample of included studies to ensure the usability and consistency of their application, including the proposed adaptions.

One researcher (MSc) applied the assessment to all included studies, complemented by an independent double-review by another researcher (MSu) on a randomised 25% of the studies. Independent assessments were conducted using the Covidence systematic literature tool, which ensured that reviewers were blinded to each other’s ratings. Any disagreements were resolved through discussion.

Although the two risk-of-bias tools use different terminologies, with ‘high risk’ in ROB2 corresponding to ‘critical risk’ in ROBINS-I, these categories were considered comparable for the purpose of synthesis and were hence discussed jointly. This approach is justified by the shared conceptual framework of both tools, which assess similar domains of bias adapted to different study designs.

No study was excluded solely due to a high or critical risk of bias. However, findings from such studies were interpreted with caution. In addition, Fisher’s exact tests were performed to determine whether the assessed risk of bias was associated with the reported effectiveness of the intervention.

### Screening and data extraction strategy

The literature search produced 3994 records across all databases. The number of records retrieved from each database is provided in online supplemental appendix 5. All records were imported into the Covidence systematic review software.^47^ Duplicate records were identified and removed automatically in Covidence (n=1437), with an additional 90 duplicates removed manually. After de-duplication, 1816 titles and abstracts were screened by the first author (MSc). A sample of 10% of the titles and abstracts were randomly selected for independent double-review by the second author (MSu). At all stages of screening, Covidence’s built-in blinding functionality ensured that each reviewer’s decisions remained concealed from the other until both had completed their independent assessments, preventing any influence between reviewers. Subsequently, the first author evaluated 168 full-text papers against the pre-established inclusion and exclusion criteria, of which 50% were randomly selected for independent review by coauthor MSu, resulting in 50 articles for the final analysis. Inter-rater agreement was 83% at title and abstract screening stage and 82% at full-text screening stage, indicating substantial agreement. Disagreements between reviewers at any stage of the review process were resolved through discussion between both researchers.

A data extraction table was designed based on both the Covidence Data Extraction Guide^48^ and on the systematic extraction frameworks used by Saran *et al*^38 42^ to record study characteristics, population, intervention design, outcomes and results (see full extraction table in online supplemental appendix 6). Researcher MSc completed data extraction for all included studies, while the second researcher (MSu) independently extracted data from 20% of the studies. Disagreements were again resolved through discussion. When relevant data were not available in the main publication, associated protocols were consulted where available.

### Data synthesis and presentation

Data were synthesised and structured by intervention category, ecological level, outcome domain and age group, using the combined CBR-ecological framework described in sections *Types of interventions* and *Types of outcome measures*. This grouping was chosen because the heterogeneity of intervention types, populations and outcome measures precluded statistical pooling. The theoretical basis for expecting these interventions to improve social inclusion outcomes is described above in the Introduction, drawing on the ecological model of Simplican *et al*^3^ and the WHO CBR framework.^4^ Following data extraction, three new intervention categories were created for studies meeting all inclusion criteria but not fitting the existing matrix: ‘moving from institutional to community living’, ‘supported internship and work skills’ and ‘promoting electoral participation’. In addition, a disaggregation by age group was undertaken, as the diversity of populations identified across included studies suggested this would provide meaningful insight into potentially differential effectiveness between children and adults.

Meta-analysis and effect-size pooling were not appropriate for this review, as the included studies differed considerably in study design, intervention type, outcome constructs and reporting metrics, seriously compromising direct statistical comparison of effect estimates. The synthesis method used was therefore vote counting based on direction of effect, complemented by textual description and tabulation of study characteristics and findings, following the Synthesis Without Meta-analysis (SWiM) reporting guideline.^49^

The standardised metric was the direction of effect based on statistical significance as reported by study authors. Where available, effect sizes, p values and CIs were also extracted. Studies were classified as ‘positive’ if all social inclusion outcomes reached statistical significance in the expected direction; as ‘no effect’ if none did; as ‘mixed’ if some outcomes were significant while others were not; or as ‘negative’ if all outcomes showed a statistically significant effect in the opposite direction. Mixed-effect studies were subsequently disaggregated to the outcome level, with each individual outcome reclassified as positive, no effect or negative. Fisher’s exact tests were used to explore associations between effectiveness and intervention category, ecological level and outcome category, as well as between effectiveness and risk of bias. These tests were selected due to small sample sizes and low expected cell counts in several subgroups, which limited the applicability of χ^2^ or other parametric tests.

All 50 included studies were considered in the synthesis regardless of methodological quality, to provide a comprehensive overview of the available evidence. Findings from studies rated as high or critical risk of bias were interpreted with caution, and the association between risk of bias and reported effectiveness was explored using Fisher’s exact tests. Certainty of evidence was not formally assessed, as the heterogeneity of study designs and outcome measures combined with the use of vote counting did not allow such judgement; risk of bias was therefore used as a proxy indicator. Heterogeneity was investigated informally by structuring results across subgroups and using Fisher’s exact tests to explore associations between subgroup characteristics and effectiveness. These analyses were largely exploratory and not pre-specified in the protocol.

**Figure 1:**
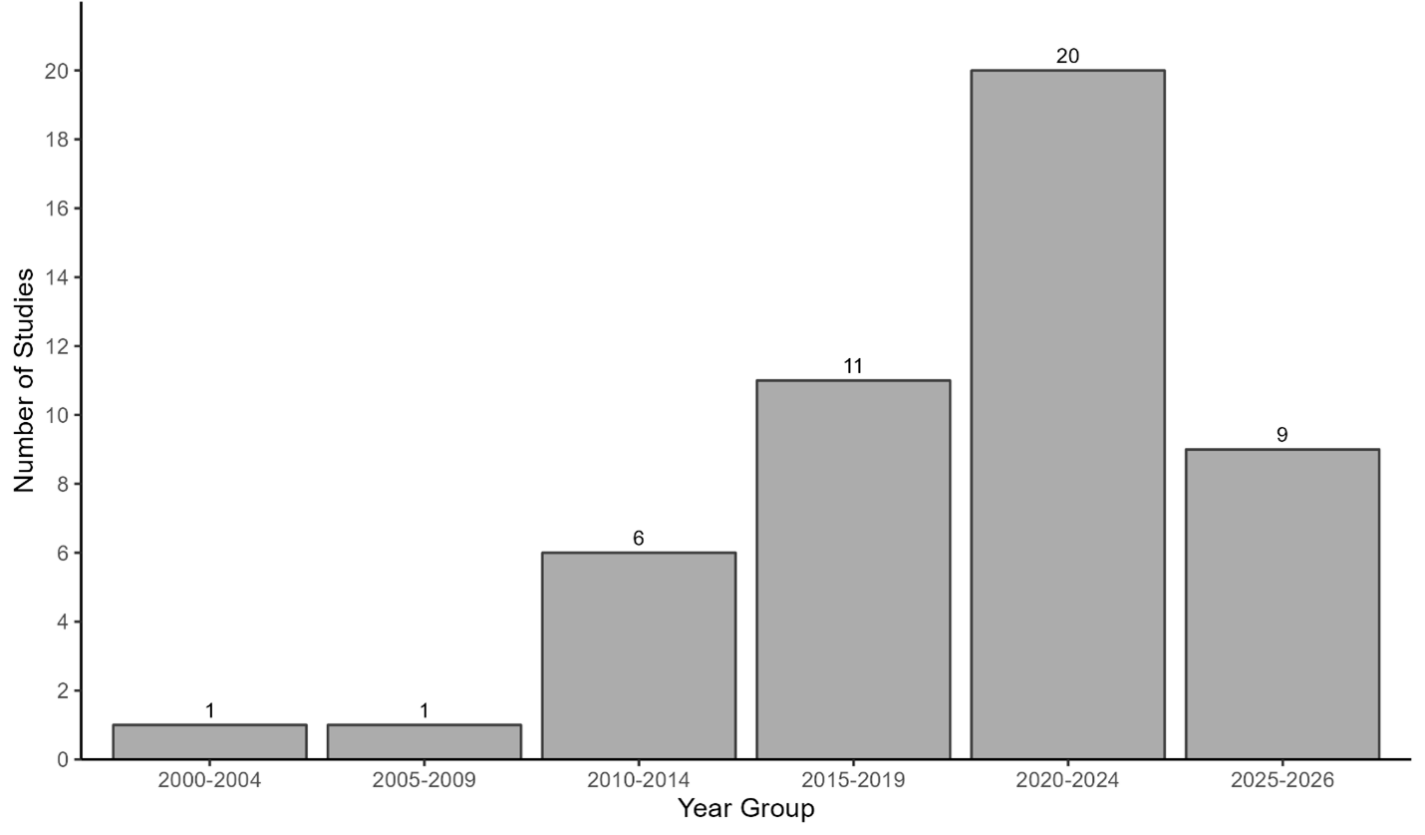
Numbers of studies published per 5-year period.

**Figure 2:**
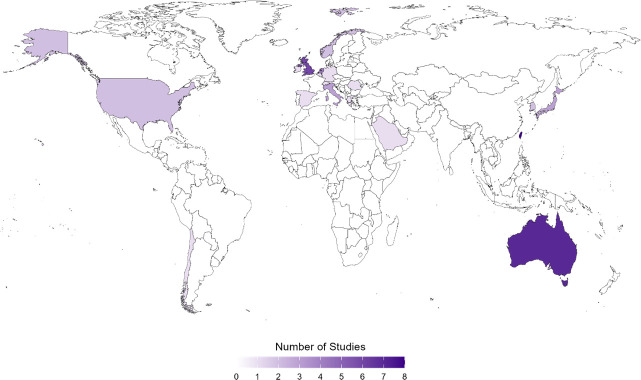
World map showing the geographical distribution of included studies (n=X). Country shading reflects the number of studies conducted in each region, ranging from 0 (white) to 8 (dark purple).

**Figure 3:**
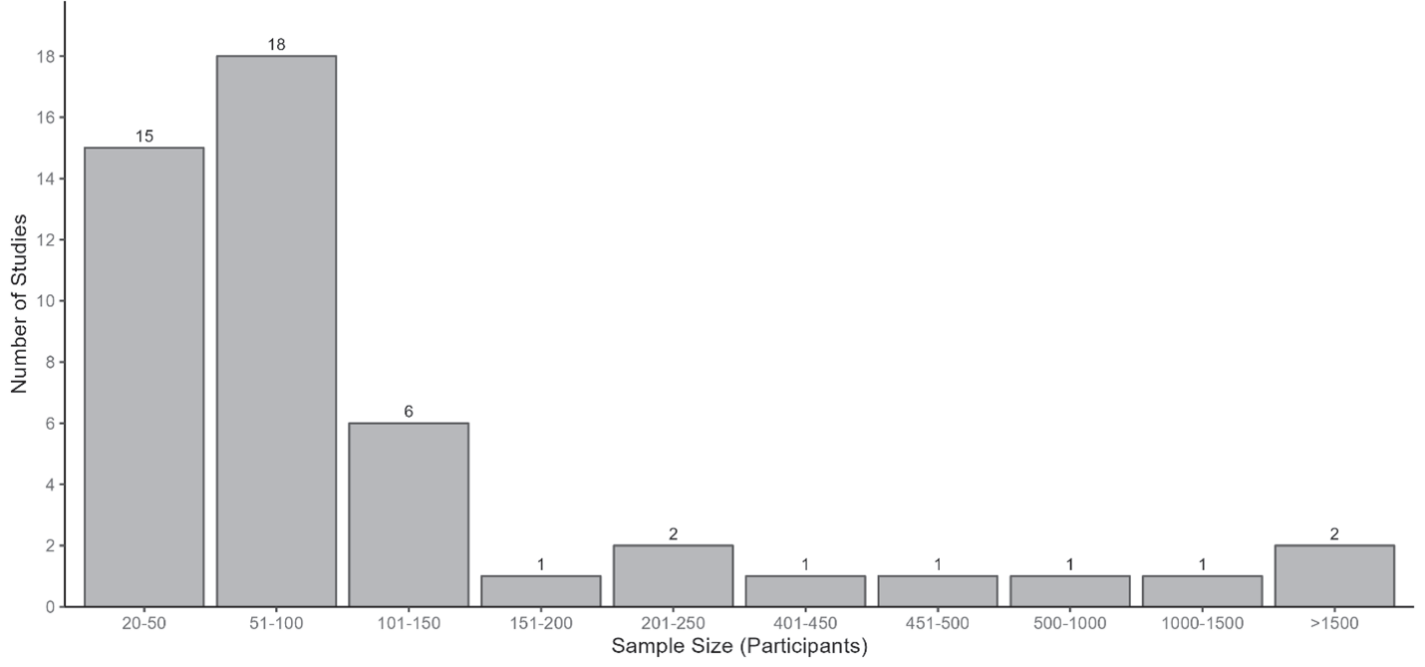
Distribution of included studies in the systematic review by sample size, grouped in intervals of 50 participants for the range 20–500. No studies were identified with sample sizes between 251 and 400 participants. For larger sample sizes, broader intervals of 500 participants were applied (500–1000, 1000–1500 and >1500).

**Figure 4:**
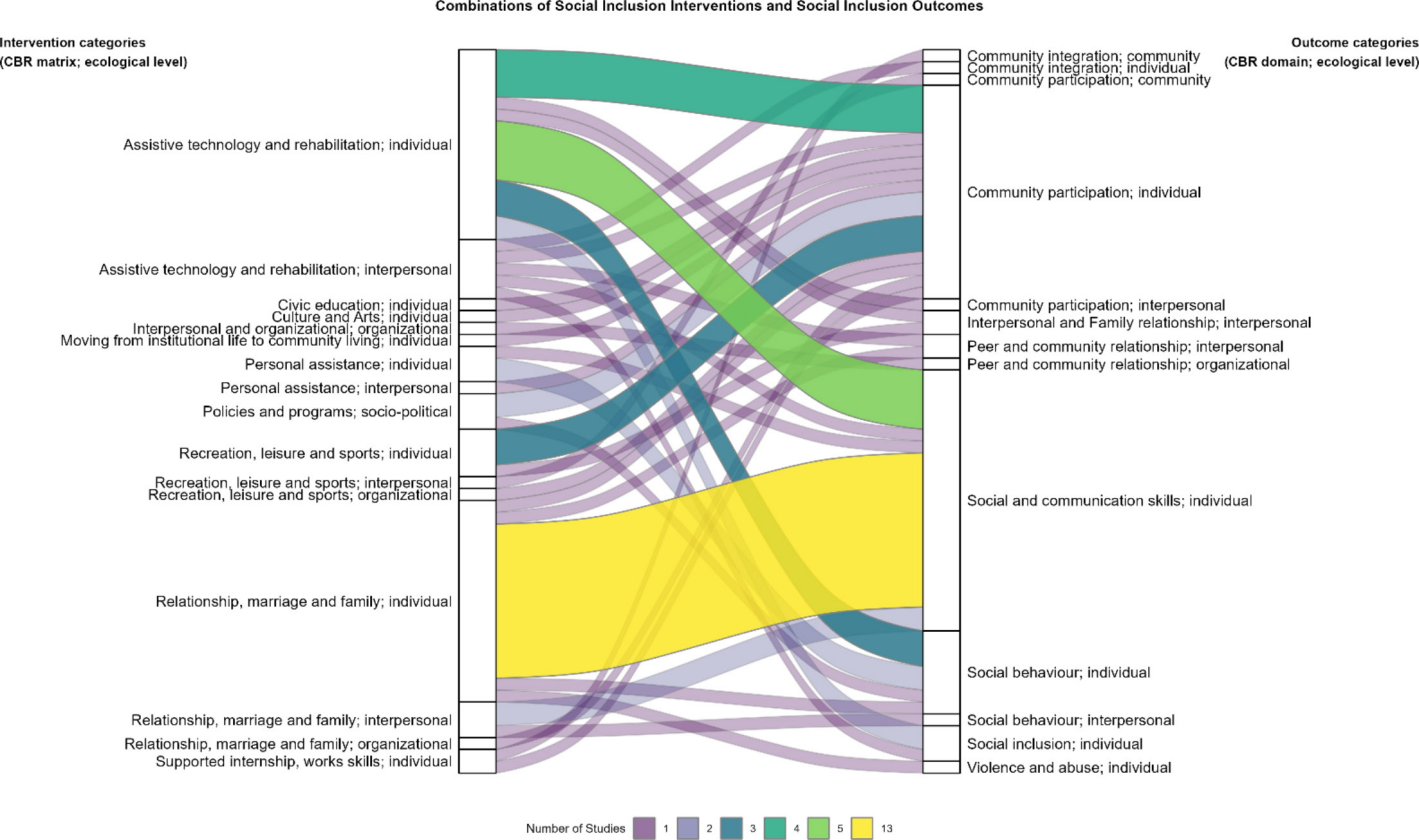
Combinations of social inclusion interventions and social inclusion outcomes. CBR, community-based rehabilitation.

Results are presented in a compact summary table in the online supplementary appendix 7, with studies ordered by effectiveness, alongside a detailed extraction table in the online supplementary appendix 8. Risk of bias is presented in two separate colour-coded tables, one for RCTs, based on ROB2 (online supplemental appendix 11), and one for non-randomised studies, based on ROBINS-I (online supplemental appendix 11). The filled-in classification matrices present the distribution of studies across intervention and outcome categories, and selected figures illustrate the distribution of studies by publication year (figure 1), country (figure 2), sample size (figure 3) and intervention-outcome combinations (figure 4).

### Patient and public involvement

No patients or members of the public were involved in developing the research question, designing the protocol, performing the review or interpreting the results.

## Results

### Study selection and characteristics

**Figure 5:**
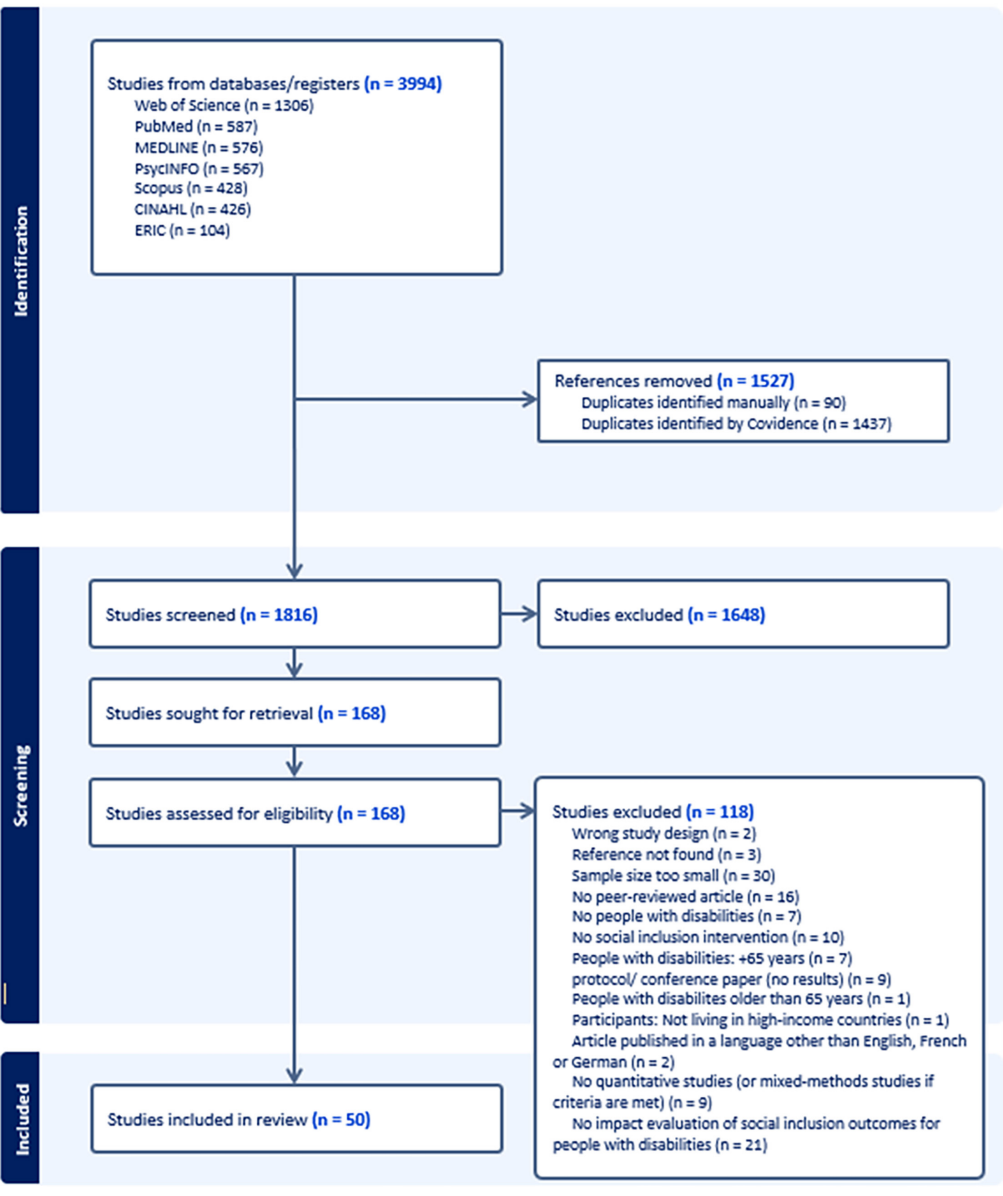
PRISMA (Preferred Reporting Items for Systematic Reviews and Meta-Analyses) flowchart.

The initial search identified 3994 records. After removing duplicates and screening titles, abstracts and full texts, 50 studies were included (see PRISMA flow diagram, figure 5). The included studies are summarised in detail in online supplemental appendix 8 and in a compact summary table presenting intervention characteristics and effects by CBR domain and ecological level (online supplemental appendix 7).

Most studies were published between 2015 and 2026 (see figure 1). Taiwan and Australia were the most frequently represented countries (eight and seven studies respectively), followed by the UK and the Netherlands, with additional studies from Europe, Oceania, North America, South America, Asia and the Middle East (see figure 2).

Study designs included 27 RCTs, 9 non-RCTs and 14 single-group before-and-after studies. While the latter two categories are both non-randomised in nature, they are distinguished here by the presence or absence of a control group: non-RCTs included a comparison group without random allocation, while before-and-after studies measured outcomes in a single group without a control group.

Sample sizes ranged from 21 to 1845 (median=71, mean=207) (see figure 3).

### Participants and disability characteristics

25 studies focused on adults with disabilities, 21 on children and 4 included both. Most interventions related to individuals with a specific type of disability, with only three studies involving more than one disability type,^50 51^ and one study targeting students rather than people with disabilities directly, aiming to enhance social inclusion through a disability awareness programme.^52^ Studies targeted a wide range of disability groups. Autism spectrum disorder was the most represented condition (n=16), predominantly in children,^53^,^68^ followed by intellectual or developmental disabilities (n=8),^69^,^76^ severe or chronic mental health conditions, predominantly in adults (n=8),^77^,^84^ stroke and aphasia (n=4)^85^,^88^ and traumatic brain injury (n=3).^89^,^91^ Fewer studies included individuals with physical disabilities (n=2),^92 93^ cerebral palsy (n=2),^51 94^ visual disabilities (n=1),^95^ Rett syndrome (n=1),^96^ chronic musculoskeletal pain (n=1)^97^ or Parkinson’s disease (n=1).^98^

### Types of social inclusion interventions

Intervention classification was based on the ecological-CBR social intervention matrix (see online supplemental appendix 9 for details). Most interventions were delivered at the individual level (n=41), followed by interpersonal (n=15), organisational (n=3), socio-political levels (n=2), and community level (n=1). Five studies examined interventions operating at both the individual and interpersonal level simultaneously.

As for the social inclusion intervention categories, the majority of the interventions reviewed were in ‘relationship, marriage and family’ (n=27), followed by ‘assistive technology and rehabilitation’ (n=20). Six studies have addressed interventions in relation to the ‘recreation, leisure and sports’ category and four studies in the ‘personal assistance’ category. Two studies have tested the outcomes of a social inclusion intervention in relation to ‘policies and programmes’, one study examined interventions addressing ‘culture and arts’. No study could be assigned to the ‘justice’ category.

The most frequently completed boxes in the matrix were ‘individual; assistive technology and rehabilitation’ and ‘individual; relationship, marriage, and family’ (both n=17), followed by ‘interpersonal; relationship, marriage, and family’ (n=11).

Some studies could not be classified within the existing matrix categories, prompting the creation of new intervention categories: three studies were classified as ‘individual; moving from institutional to community living’, two studies as ‘individual; supported internship and work skills’ and one study as ‘individual; promoting electoral participation’.

### Outcome characteristics

Outcomes were mapped using the ecological-CBR outcome matrix (see online supplemental appendix 10 for details). Most were assessed at the individual level (n=45), with interpersonal-level outcomes being less common (n=6); organisational and community-level outcomes were rare (n=1 each). Two studies examined social inclusion outcomes at both the individual and interpersonal levels, one study combined interpersonal and organisational level outcomes and another study assessed outcomes at both the interpersonal and community levels. No study was identified that targeted an outcome at the socio-political level.

Most social inclusion outcomes focused on assessing the impact of social inclusion interventions on ‘community participation’ (n=25), followed by ‘social and communication skills’ (n=23). 15 studies examined the effects of interventions on ‘social behaviour’ and 13 studies on ‘interpersonal and family relationships’. Four studies each fell into the category of ‘social inclusion’ and ‘community integration’, another two under ‘peer and community relationships’ and one study in the outcome category ‘violence and abuse’. No studies addressed the ‘social domain’.

In the matrix, the most prevalent combination of outcome categories and ecological levels was the box ‘individual; community participation’ (n=24). This was followed by ‘individual; social and communication skills’ (n=22), ‘individual; social behaviour’ (n=14) and ‘individual; interpersonal and family relationships’ (n=11).

### Combinations of social inclusion interventions and social inclusion outcomes

Intervention-outcome combinations were highly varied. The most commonly observed pairing was the intervention category ‘relationship, marriage and family; individual’ targeting the outcome category ‘social and communication skills; individual’ (n=13), followed by five studies pairing ‘assistive technology and rehabilitation; individual’ with ‘social and communication skills; individual’ outcomes and four studies combined ‘assistive technology and rehabilitation; individual’ with the outcome category ‘community participation; individual’. Three studies paired ‘recreation, leisure and sports; individual’ with ‘community participation; individual’. These intervention-outcome combinations are visually represented in figure 4.

### Intervention effectiveness

A variety of measurement tools were used to assess social inclusion in the included studies, including the Social Responsiveness Scale (n=13), the Canadian Occupational Performance Measure (n=7) and Goal Attainment Scaling (n=7). Other tools included the Social Functioning Scale and the Participation Assessment with Recombined Tools-Objective (n=3), as well as several context-specific measures (see online supplemental appendix 8 for further details).

Respondents included individuals with disabilities and proxies, such as parents, clinicians and support staff. 16 adult-focused studies relied solely on self-report, while seven combined self and proxy reports (including via support staff, medical staff and/or volunteers). Two adult studies additionally included parents as proxy respondents. In studies involving children, outcome data were most commonly obtained from a combination of child self-report and parent report (n=5), while six studies relied on parents only, and two on children alone. Four studies used mixed reporter combinations including teachers, medical staff and/or parents. One study used researchers as the sole assessors. Two studies used responses from non-disabled individuals—one from school pupils^52^ and one from university students^99^—to assess attitudes toward peers with disabilities (see online supplemental appendix 8 for further details).

Effectiveness was categorised as positive (all outcomes statistically significant in the expected direction), no effect (no outcomes statistically significant) or mixed (some outcomes significant, others not), based on statistical significance as reported by study authors. Among the studies reviewed, 20 demonstrated statistically significant positive effects of the social inclusion intervention^5152 55 58 63 65^,^68 72^ (n=2384 participants), while 14 studies found no effects^6471 75 77^,^81 84 86 87 89 92 94^ (n=3481 participants) and 16 studies showed mixed results, with some outcomes exhibiting positive effects and others yielding non-significant results^5053 54 56 57 59^,^62 69 69 82 90 91 95 96^ (n=5039 participants), across a total of 10 904 participants. It should be noted that overall participant numbers were heavily influenced by a small number of large studies.

When classified by intervention type, the categories ‘relationship, marriage, and family; Individual’ (n=9), followed by ‘assistive technology and rehabilitation; individual’ had the highest number of positive-effect studies (n=7). However, the latter also included the highest number of studies reporting no significant effects (n=5). Mixed results were distributed across multiple categories, with the highest number of mixed-effect studies found in studies targeting intervention in ‘relationship, marriage, and family; individual’ (n=6) and ‘assistive technology and rehabilitation; individual’ (n=4). No significant association was found between intervention category and effectiveness (p=0.278), and between effectiveness and ecological level (p=0.947).

In order to unpack the potential nuance captured by the often multiple within-study results (which is particularly relevant for the studies we labelled as ‘mixed results’), we disaggregated the effect estimates of all 50 studies at the outcome level, then classifying the result for any given outcome as either positive, null or negative effects. Positive effects were most often observed in ‘social and communication skills; individual’ (n=17), followed by ‘social behavior; individual’ (n=10) and ‘community participation; individual’ (n=10), while null effects were most commonly found in the latter (n=15). Statistical analysis using Fisher’s exact test found a significant association between outcome category and effectiveness (p=0.017), but no significant association between ecological level and effectiveness (p=0.358).

### Age: grouped disaggregated findings

Of the 50 included studies, 21 focused exclusively on children, 25 on adults and 4 included mixed-age samples. As shown in table 3, the two age groups differed markedly in disability type, intervention type and outcome domain.

**Table 3:** Intervention characteristics, outcome domains and effectiveness by age group

	Child-only (n=21)	Adult-only (n=25)	Mixed-age (n=4)
Effectiveness			
Positive effect	n=10 (48%)	n=10 (40%)	n=3 (75%)
No effect	n=3 (14%)	n=11 (44%)	n=0 (0)
Mixed effect	n=8 (38)	n=4 (16)	n=1 (25)
Main disability type			
Autism spectrum disorder	n=16 (76)	n=0 (0%)	n=0 (0%)
Mental health condition	n=0 (0%)	n=7 (28)	n=0 (0%)
Stroke/TBI/acquired disability	n=0 (0%)	n=8 (32%)	n=0 (0%)
Intellectual/developmental disabilities	n=1 (5%)	n=5 (20%)	n=1 (25%)
Other	n=4 (19%)	n=5 (20%)	n=3 (75%)
Main intervention type			
Social skills training	n=14 (67%)	n=0 (0%)	n=1 (25%)
Rehabilitation/community support	n=5 (24%)	n=19 (76%)	n=1 (25%)
Supported employment	n=0 (0%)	n=2 (8%)	n=0 (0%)
Arts/culture/recreation	n=1 (5%)	n=2 (8%)	n=1 (25%)
Other	n=1 (5%)	n=2 (8%)	n=1 (25%)
Main outcome domain targeted			
Social and communication skills	n=18 (86%)	n=3 (12%)	n=2 (50%)
Community participation	n=2 (10%)	n=22 (88%)	n=1 (25%)
Other outcomes	n=1 (4%)	n=3 (12%)	n=1 (25%)

Percentages are calculated within each age group. Studies may target multiple outcome domains; percentages may therefore exceed 100%.

TBI, traumatic brain injury.

Child-focused studies were predominantly conducted with children with autism spectrum disorder (n=16; 76%), and the large majority targeted social and communication skills outcomes (n=18; 86%) through social skills training interventions (n=14; 67%). Positive or mixed effects were observed in 86% of child-focused studies (positive: n=10; 48%; mixed: n=8; 38%), with 14% (n=3) reporting null findings.

Adult-focused studies covered a broader range of disability types, most commonly mental health conditions (n=7; 28%), stroke (n=3; 12%) and traumatic brain injury (n=3; 12%). The majority targeted community participation outcomes (n=22; 88%) through rehabilitation or community support interventions (n=19, 76%). Null findings were substantially more frequent in adult-focused studies (n=11; 44%) compared with child-focused studies (n=3; 14%). Among adult studies, those involving participants with mental health conditions showed the highest proportion of null findings (n=6 out of 7; 86%). When mental health condition studies are excluded, the positive effect rate in adult studies was 50% (n=9 out of 18). By contrast, studies involving children with autism spectrum disorder showed the highest rate of positive or mixed results across all disability groups, with 94% (n=15 out of 16) reporting positive or mixed findings and only 6% (n=1) reporting null findings.

### Risk of bias

Using ROB2 and ROBINS-I, 21 studies were rated as moderate risk or ‘some concerns’, 21 as high or serious risk and 8 as low risk. All low-risk studies were RCTs. The most common source of bias in non-randomised studies was confounding (15 of 23 studies assessed with ROBINS-I) (see online supplemental appendix 11 for further details). A Fisher’s exact test showed no significant association between risk of bias and reported intervention effectiveness (p=0.621), indicating that studies with higher quality in their research design were not systematically more or less likely to report positive outcomes.

## Discussion

### Statement of the principal findings

This systematic review synthesised evidence from 50 studies on interventions aimed at promoting social inclusion among people with disabilities in high-income countries. The interventions identified were diverse in type, target populations and settings, reflecting the multifaceted nature of social inclusion. Despite this diversity, clear patterns emerged.

As for the key point of intervention effectiveness, the evidence base is mixed. 40% of the studies reported statistically significant positive effects of the interventions on at least some social inclusion outcomes. For example, social skills group training for children or adolescents with autism spectrum disorder led to improved social communication abilities, and certain rehabilitation or assistive technology programmes improved participants’ community participation or daily functioning. However, about one-quarter found no significant effects of the intervention on the targeted inclusion outcomes. About one-third reported mixed results, meaning some outcome measures improved while others showed no change. Notably, no study reported overall negative effects or harm, but the absence of positive change in a substantial portion of the interventions might suggest that several current approaches have only limited impact on social inclusion.

Several factors may help explain why effects were inconsistent across studies. First, the interventions were highly heterogeneous in content, intensity, duration and target population, which limits comparability across studies. Second, the strongest effects were seen for more proximal outcomes such as social and communication skills and social behaviour, whereas null findings were more common for broader community participation outcomes—a pattern that likely reflects the fact that proximal skills are more directly trainable, while broader participation depends on environmental accessibility, social attitudes, organisational openness and wider structural supports, and is therefore harder to change through individual-level approaches alone.^36^ Indeed, even when an intervention successfully builds an individual’s capacity or confidence, additional barriers in the environment—physical, social or attitudinal—may still limit actual inclusion.^15 19 36^ For instance, teaching social skills to a child with autism spectrum disorder can yield improvements in a clinical setting or school context,^100^ but if peer attitudes remain negative or activities are not adapted to be inclusive, broader participation in community life may not increase.^101^ Third, many studies measured outcomes only shortly after the intervention, which may have been insufficient for more distal changes in participation or belonging to emerge. Social inclusion is a dynamic process that may evolve over time, and brief follow-up periods may not adequately capture whether intervention effects are sustained. Finally, the evidence base used diverse and sometimes narrow outcome measures, which may not have captured subjective or longer-term dimensions of inclusion. Taken together, these factors suggest that many interventions may be capable of improving discrete interpersonal skills without necessarily producing broader social inclusion when contextual barriers remain unchanged.

Statistical tests revealed no significant relationship between intervention categories and effectiveness, nor between ecological level and effectiveness. However, a significant association was found between outcome category and effectiveness, suggesting that the type of outcome targeted plays an important role in determining whether an intervention succeeds. Specifically, positive effects were most frequently observed for outcomes related to social and communication skills (n=17) and social behaviour (n=10), while null effects were most commonly found for community participation outcomes (n=15). This may reflect the fact that social and communication skills are more proximal and directly trainable outcomes, whereas community participation is a broader and more distal outcome, influenced by a wider range of contextual and societal factors beyond the intervention itself. This aligns with Saran *et al*,^38^ who found a large effect for skills-based interventions, but more variable effects for broader social inclusion outcomes, and with Shaw *et al*,^102^ who argued that interventions addressing only surface-level barriers tend to result in short-term and superficial forms of inclusion. This observed heterogeneity in the current evidence suggests that the success of a given intervention may depend not only on the type of intervention, but also on the precise outcome being targeted and on the complexity of the social context, in which the intervention is delivered. This supports the understanding that social inclusion is multifaceted, and that improvements can come from various routes (social skills training, employment support, peer mentorship, community activities, etc), but each route must effectively engage participants and tackle relevant barriers to make a difference.^103^

When examined by age group, child-focused studies more often reported positive or mixed results, while adult-focused studies showed a higher proportion of null findings—a distinction that of course does not mean that child-focused interventions are inherently more effective than those for adults. In the studies reviewed, the two age groups differed substantially not only in disability type and outcome domain, but also in the type of intervention used. Child-focused studies predominantly used social skills training, while adult-focused studies predominantly used rehabilitation or community support approaches. Some research has demonstrated that social skills training for children with autism spectrum disorder produces small to moderate positive effects,^100^ though effects are frequently not robust across all outcomes.^104^ A closer examination by disability type further suggests that the concentration of mental health-focused studies in the adult group largely may explain the difference in effectiveness: studies involving adults with mental health conditions showed the highest rate of null findings of any disability group, with 86% reporting no significant effects. Killapsy *et al*^105^ concluded that social interventions for people with severe mental illness are among the most complex to implement effectively and require multilevel stakeholder commitment. Furthermore, qualitative evidence suggests that barriers such as stigma, structural exclusion and the multidimensional nature of social inclusion operate across almost every aspect of life for people with severe mental illness, including their own self-perception.^106^

When mental health-focused studies are excluded, adult studies showed a positive effect rate comparable to studies on children. By contrast, studies involving children with autism spectrum disorder showed the highest rate of positive or mixed results, likely reflecting the strong evidence base for social skills training targeting social and communication skills in this population—the outcome domain most consistently associated with positive effects in this review.

These findings suggest that disability type, intervention type and outcome domain are stronger predictors of effectiveness than age group per se, and that interventions focused on social and communication skills, particularly those embedded in structured skills training models, showed more consistent positive results than those targeting broader community participation outcomes, a finding consistent with the observation that community participation outcomes are complex and difficult to change through individual-level approaches alone.^36^ Mixed interventions that combined multiple domains or ecological levels did not consistently outperform more focused ones.

The majority of interventions operated at the individual level of the social-ecological model of inclusion^3^ often targeting the improvement of personal skills or capacities (eg, through assistive technology, rehabilitation programmes or social skills training). A much smaller subset of interventions included elements that addressed interpersonal factors, community, organisational change or broader socio-political elements. This imbalance suggests that interventions to date have predominantly viewed social inclusion as an individual outcome rather than a shared community or societal responsibility. Many studies have been published only in the last decade, indicating that research on social inclusion interventions has been growing recently, possibly in response to international policy attention (eg, the UNCRPD).

The outcomes assessed also reflected an individual-level focus (eg, personal skill acquisition, community participation), similar to the intervention-focus. Far fewer studies measured change at interpersonal, community or organisational levels. Importantly, no studies examined outcomes related to social identity, empowerment in the justice system or experiences of reduced stigma/abuse. This indicates that certain dimensions of social inclusion, for example, feeling a sense of belonging or changes in societal attitudes, self-determination and rights, have not been well captured in intervention studies to date. This gap is particularly notable because existing high-level frameworks such as the UNCRPD explicitly emphasise rights, civic participation and freedom from discrimination as core dimensions of inclusion—dimensions that remain largely absent from the intervention research base.^9^

Data were often collected from multiple perspectives, including self-reports and proxy reports by parents or professionals, particularly for children or individuals with communication difficulties, while others relied solely on proxies without directly engaging people with disabilities. This raises concerns about the extent to which these studies uphold the principle of self-representation by people with disabilities.^107 108^

Research has shown that agreement between self-report and proxy-report in disability populations is mixed,^109^ and that proxy respondents may apply more clinical or external thresholds, resulting in systematic differences in how outcomes are classified,^110^ introducing a potential source of measurement bias particularly in studies relying entirely on caregiver or professional report.

We also assessed the methodological quality of the evidence base. The risk of bias was often moderate to high. All low-risk studies were RCTs. Common methodological limitations included lack of blinding, absence of control groups in non-randomised designs and unaddressed confounding in observational studies, where none of the non-RCTs achieved a low risk of bias. These issues highlight the need to interpret those results in light of potential biases that could inflate the perceived effectiveness. Several studies with serious risk (eg, studies by Ison *et al*, Tateno *et al*, Caliendo *et al*, Alecu *et al* and Imamoto *et al*^52 63 65 68 83^) reported strong positive effects, underscoring the importance of considering both effect size and study quality. Conversely, some rigorous studies (eg, studies by Sanches *et al* and Borgen *et al*^80 89^) failed to find significant effects, which may indicate that truly effective inclusion strategies have been challenging to identify or implement based on the hitherto most frequently used approaches. However, a Fisher’s exact test showed no significant association between risk of bias and reported intervention effectiveness, indicating that studies with higher methodological quality were not systematically more or less likely to report positive outcomes.

Taken together, these findings suggest that the current portfolio of social inclusion interventions addresses only part of the inclusion puzzle. The individual-centric focus may reflect the greater ease of designing and evaluating person-level interventions, but also a possible misalignment between what may be needed for full inclusion—changes at community, organisational and structural levels—and what is currently being tested.^36 38 102 111 112^

This gap is noteworthy because true social inclusion arguably requires changes at those higher levels. For example, an individual’s social skills may improve, but without welcoming community spaces or inclusive organisational practices, that person may still remain socially isolated or excluded.^3 36 113^ The relative evidence gap could be due to multiple factors: individual-level interventions are often easier to design, standardise and evaluate within the timeframe of a study, whereas community or policy-level interventions tend to be more complex, slower to show impact and harder to fit into experimental research designs. It may also reflect historical service models that focus on rehabilitating or ‘fixing’ the person with a disability rather than modifying societal barriers, a critique often raised in disability studies.^111 112^

It is also possible that current outcome measures are not fully capturing the nuances of inclusion—for example, an intervention might improve a person’s sense of belonging or confidence in subtle ways that standard scales or short follow-up periods fail to detect. This highlights a persistent challenge in the field: measuring social inclusion is inherently difficult, as it spans subjective feelings, objective participation indicators and societal conditions.

Lastly, our findings reflect certain gaps in the literature that carry conceptual implications. One objective of this study was to examine whether effectiveness varied by disability type and contextual factors. The majority of reviewed studies focused on a single type of disability with no insights into whether interventions are effective across diverse disability situations. Only one study, targeting individuals with traumatic brain injury, took disability severity into consideration; however, this study found no heterogeneity effect. The age-disaggregated analysis conducted in this review provides an initial step in this direction, suggesting that disability type and outcome domain might be stronger predictors of effectiveness than age group per se, and that future research should systematically examine how intervention outcomes differ across factors such as age, disability type, severity and socio-economic context. In addition, the absence of studies addressing outcomes related to ‘social identity’ or targeting justice and rights indicates that some dimensions of inclusion, particularly those related to empowerment and societal change, are neglected in intervention research. These are domains strongly emphasised in frameworks and by disability advocates; for instance, being free from abuse and having one’s rights respected are fundamental to inclusion.^9 22 114 115^ The lack of intervention studies focusing on these outcomes might suggest that such issues are being handled through policy and advocacy rather than formal intervention trials. This points to a possible disconnect between policy aspirations (eg, those outlined in international conventions or national strategies which call for inclusive societies at all levels) and the research evidence base that is informing practice. Thus, the interpretation of our review is that while progress has been made in developing and testing interventions for social inclusion, a more holistic approach aligning with the full breadth of the inclusion concept is still lacking.

### Strength and weakness of the study

While a number of studies incorporated both an intervention group and a control group, relatively few studies employed an active control group, in which participants engaged in an alternative activity not designed to influence social inclusion outcomes. The absence of active control groups limits the ability to determine whether observed effects are attributable to the specific components of the intervention or to non-specific factors, including increased attention, social interaction or changes in routine. This is a particularly important limitation in social inclusion research, where the social and relational components of participation in any structured group activity may themselves produce inclusion effects, independently of the specific intervention content.^105^

This review benefited from a comprehensive and systematic search across seven databases with no language restrictions, updated in 2026 to capture the most recent evidence. The review was preregistered on OSF, ensuring transparency and limiting the risk of outcome reporting bias (https://osf.io/9wfqy). Blinded independent double-screening and double-extraction at multiple stages strengthened the reliability of study selection and data extraction. Unlike previous reviews focusing on specific disability types, this review adopted a cross-disability scope, providing a broader evidence base. Risk of bias was assessed using validated tools (ROB2 and ROBINS-I) applied according to study design, with an adaptation of ROBINS-I for single-group before-after studies allowing bias assessment for a more inclusive synthesis. Another strength was the mapping of findings onto a comprehensive theoretical framework, which helped identify overarching themes beyond those of individual studies. Together, these approaches helped ensure a broad yet structured synthesis.

Nonetheless, this review and the underlying evidence base present several limitations. Many included studies had small sample sizes, limited diversity or lacked robust designs, issues also noted in previous reviews.^25 29 116^ A substantial number were non-randomised or poorly controlled, raising concerns about risk of bias. The heterogeneity of outcome measures further complicates interpretation, with ‘social inclusion’ defined and assessed inconsistently. Few studies included long-term follow-ups, despite the time it may take for inclusion outcomes to materialise.

Our review was limited to high-income countries and quantitative studies with sample sizes above 20, which may have excluded valuable insights from small-scale or qualitative research.^117^,^120^ Publication bias is possible, though the presence of numerous null-effect studies suggests a mix of findings in the literature. The exclusion of qualitative studies, while appropriate for an effectiveness review requiring quantitative outcome data, means that important insights into participants’ lived experiences, implementation processes and intervention mechanisms are not captured in this synthesis. A complementary review incorporating qualitative evidence would be valuable to provide a fuller understanding of how and why social inclusion interventions work.

Classifying interventions and outcomes required some subjective judgement, and the statistical analysis was constrained by small group sizes, reducing power to detect patterns. While we found some associations (eg, between intervention domain and effectiveness), these should be interpreted cautiously. Fisher’s exact test, used due to small subgroup sizes, has limited power, so non-significant results should not be over-interpreted as proof of no difference; it may simply reflect insufficient sample size rather than no effect.

While age-disaggregated patterns were described, the review was not designed or powered to support fine-grained subgroup conclusions. The number of studies within many subgroup cells was too small for meaningful statistical comparison, and apparent differences across age groups likely reflect variation in disability type, intervention approach and outcome domain rather than age itself. The particularly high rate of null findings in studies targeting adults with mental health conditions warrants further investigation in future population-specific reviews.

In addition, the use of vote counting based on direction of effect as the primary synthesis method has inherent limitations, as it does not account for effect sizes, sample sizes or study quality, and may therefore provide an incomplete picture of the overall evidence base. A more quantitative synthesis method would have been preferable, but was infeasible, since not all included studies reported effect sizes or CIs, and the diversity of outcome measures prevented direct effect size comparisons.

### Policy implications

The main implication for policy is that interventions should be designed at the same ecological level as the outcome they seek to change. Structured skills-based interventions may be appropriate when the aim is to improve social communication or social behaviour, and this may help explain the stronger results observed in child-focused autism studies. However, if the aim is to improve community participation or broader social inclusion, interventions are unlikely to succeed if they focus on the individual alone. More effective approaches will likely need to combine individual support with measures that address environmental and organisational barriers in schools, workplaces, services and communities. Community-level initiatives—such as accessible urban design, inclusive education and employment policies and public awareness efforts—remain underused despite their potential impact, and clearer alignment between individual-focused and systemic measures, supported by intersectoral collaboration, may improve outcomes and guide more efficient resource allocation. Positioning inclusion as a health equity objective can usefully guide intersectoral action across health, social care, education and urban planning toward barrier removal and access to participation as health-relevant outcomes.^121^ For adults with mental health conditions in particular, the findings suggest that sustained community-based supports may be more appropriate than short, time-limited programmes.

### Future research directions

To more effectively inform policies to advance social inclusion for people with disabilities, future research should prioritise multilevel and community-focused interventions that address structural barriers alongside individual skills, using innovative methods such as cluster trials or natural experiments with longer follow-up periods. Intersectoral approaches combining health, education and urban planning, alongside expanded outcome measures capturing empowerment, identity, rights and civic engagement, are needed. Improved methodological rigour—including larger well-powered samples, transparent reporting and preregistration—will also be essential to building a stronger evidence base.

Future research should also prioritise the development and psychometric validation of standardised social inclusion outcome measures that are applicable across disability types and age groups. The lack of a shared measurement framework across studies was a major source of heterogeneity in this review and prevented quantitative synthesis.

A core outcome set for social inclusion interventions, developed in collaboration with people with disabilities, clinicians and researchers, would significantly strengthen the comparability and cumulative value of future studies. Actively involving people with disabilities in designing and conducting research will help ensure interventions are relevant, respectful and impactful.^103 122^

The ultimate goal of future research should be not only to document whether an intervention works, but to understand how and why it works, for whom and under what conditions. This approach, informed by theory-driven and realist evaluation traditions,^123^,^125^ enhances the likelihood that findings can be adapted effectively across diverse real-world settings and helps narrow the persistent gap between research and practice.

## Conclusion

This systematic review found that interventions to promote social inclusion for people with disabilities in high-income countries show mixed effectiveness and remain concentrated mainly at the individual level. The clearest gains were seen in skills-focused and family-focused approaches, while broader barriers related to participation, accessibility and inclusion at community and structural levels were rarely addressed. Future research and policy should therefore prioritise more rigorous, multilevel interventions that better align with the full concept of social inclusion.

## Supplementary material

10.1136/bmjopen-2026-117213 online supplemental file 1

## Data Availability

Data are available upon reasonable request.
