# Supplementary material for: Effectiveness of interventions to promote social inclusion of people with disabilities in high-income countries: a systematic review of quantitative evaluation studies
Source: BMJ Open. 2026 Jun 16;16(6):e117213. doi: 10.1136/bmjopen-2026-117213 (PMC13288884; doi:10.1136/bmjopen-2026-117213)
Supplement: online supplemental file 1 [file bmjopen-16-6-s001.pdf]

Appendices

Appendix 1

Included and excluded criteria:

1. Included criteria

To be considered for inclusion, a study must meet the following criteria:

- Types of studies:
  - Quantitative studies, mixed methods studies (If criteria are met for the quantitative part)
  - Study assessing the impact of interventions on social inclusion outcomes for People with disabilities
  - Impact evaluation results
  - Peer-reviewed article
  - Published in English, French or German
  - The interventions(s) and the outcome(s) of the social inclusion intervention(s) should be clearly stated as the improvement of social inclusion for people with disabilities.
  - Study design:
- randomized controlled trial (RCT), non-randomized controlled trial (non-RCT, , where outcomes are measured in both an intervention and a comparison group before and after the intervention, without random allocation), single-group before-and-after (where outcomes are measured in one group only, before and after the intervention, with no control condition), interrupted time-series designs, historically controlled study, quasi-experimental methods (e.g. regression discontinuity design (RDD), difference-in-differences, matched designs) Types of participants
  - People with disabilities (sensory disabilities, physical disabilities, intellectual disabilities, cognitive disabilities, neuro-motor, neurodevelopmental disorders, and chronic psychiatric disorders)
  - Age: children and adults of working age: 0-65 years
  - Living in high-income countries (classified by the World Bank released on July 1, 2023) (If the intervention is studied in more than one country, it must be studied in at least one high-income country).
  - When the aim of the intervention is to improve the social inclusion of people with disabilities, other participants, for example, environment of people with disabilities, their family, friends, or support staff are eligible for inclusion in the study as well.
- Types of interventions
  - If the study explores interventions that aim to promote social inclusion for people with disabilities and aligns with the social inclusion intervention matrix, it will be included.
  - Other interventions, if the aim of the intervention is to promote the social inclusion of people with disabilities.
- Types of outcomes

- If the main outcomes of the study are related to changes in the social inclusion of people with disabilities and fit into the social inclusion outcomes matrix, the study will be included.
- Other outcomes may include improved social inclusion for people with disabilities.

2. Excluded criteria

Excluded criteria:

- Types of studies:
  - Qualitative studies
  - Descriptive quantitative studies
  - Process evaluations
  - Cross-sectional studies
  - If there is no comparison
  - No statistical methods used to calculate effect size
  - No peer-reviewed article
  - Reference not found
  - No impact evaluation of an intervention, or no impact evaluation related to social inclusion outcomes for people with disabilities.
- Types of participants:
  - If the participants are the community of people with disabilities, their family, friends or support staff, but the aim is not clearly stated as to promote the social inclusion of people with disabilities.
  - Age: older than 65 (for people with disabilities)
  - When no participant lives in high-income countries
  - Sample size too small < 20
- Types of interventions:
  - If the intervention's goal of improving social inclusion of people with disabilities was not clearly stated
- Types of outcomes:
  - When a study does not consider any social inclusion outcomes, it is excluded

Appendix 2

Matrix of social inclusion interventions:

	Individual*	Interpersonal*	Organizational*	Community*	Socio-political*
Personal-Assistance**					
Relationship, marriage and family**					
Culture and Arts**					
Recreation, leisure and sports**					
Justice**					
Assistive technology and rehabilitation**					
Policies and programs**					

*Social inclusion **interventions** across the five ecological levels as defined by Simplican et al. (2015):

- **Individual**
- **Interpersonal** (Attitudes of social network members, relationships with staff, family members, friends and etc., relationship between social network members)
- **Organizational** (Culture of the group home, mission statements, attitudes of staff and managers, training and opportunities for staff, organizational cultures of groups within the community (e.g. schools, employment centres, law enforcement), access to communication services)
- **Community** (Availability of appropriate services, access to appropriate services, transportation, online communities, resources, community attitudes, culture, geography and discourse)
- **Socio-political** (laws, legal enforcement, marked forces, state perspectives, histories of service delivery, legislative cutbacks)

Social inclusion **Interventions related to the social pillar of the CBR matrix (WHO, 2010) further developed by Saran et al. (2021).:

- **Personal assistance** (Formal personal assistance and support, Informal personal assistance and support)
- **Relationship, marriage and family** (Networking and social support, Improving community attitude, Community attitude, social and communication skill training, violence prevention interventions)

- **Culture and arts** (Access and participation in cultural programs, arts, drama and theatre, access and participation in religious activities)
- **Recreation, leisure and sports** (Access and participation in sports events, Access and participation in recreation and leisure)
- **Justice** (Accessibility of legal system and justice, Access to legal system and justice)
- **Assistive technology and rehabilitation*** (Assistive technology, Rehabilitation, Medical care)
- **Policies and programs** (International legislation and policies, Social inclusion policies)

Appendix 3

<u>Matrix of social inclusion outcomes:</u>

		Individual*	Interpersonal*	Organizational*	Community*	Socio-political*
**Social****	Social identity**					
	Personal assistance**					
**Skills for social inclusion****	Social and communication skills**					
	Social behaviour**					
**Broad based social inclusion and participation measure****	Social inclusion**					
	Community integration**					
	Community participation**					
	Access to justice**					
**Relationships****	Interpersonal and Family relationship**					
	Peer and community relationship**					
	Violence and abuse**					

*Social inclusion **outcomes** related to Simplican et al. (2015) ecological pathways levels:

- **Individual** (increased happiness, improved self-esteem, sense of belonging)
- **Interpersonal** (respect and trust between people, increased social capital)
- **Organizational** (changes in organizational culture, becoming more affirming of inclusion)
- **Community** (availability of and access to appropriate services and transportation, positive community attitudes)
- **Socio-political** (political change, laws)

**** Social inclusion **outcomes** related to the social pillar of the CBR matrix (WHO, 2010), further developed by (Saran et al., 2021):

- **Social**
  - Social identity (a person's self-concept, value and emotional significance attached to his or her membership in a social group)
  - Personal assistance (e.g. individual support plans, access to training to manage the personal assistance needs, availability of support for families who provide informal personal assistance)
- **Skills for social inclusion**
  - Social and communication skills (learned verbal and nonverbal behaviour, individual's social perceptual accuracy, transforming information [vocally, written, visually, nonverbally], availability and use of communication aids, speech and reading devices)
  - Social behaviours (behaviours that connect individuals)
- **Broad based social inclusion and participation measures**

- Social inclusion (people with disabilities are spending time outside of their house, travelling away from the house)
- Community integration (help people with disabilities optimize their personal, social and vocational competencies to live successfully in the community.)
- Community participation (participation in community activities, which may include leisure activities [hobbies, arts, sports], political or civic activities, organizations activities, productive activities [e.g. employment, education], consumption or access to goods and services, religious activities, cultural activities)
- Access to justice: (access to or interact with the legal system)

- **Relationship:**
  - Interpersonal and Family relationship (sense of belonging to a network, participation in household activities, positive behaviour of the family towards people with disabilities, family is sensitive to the interests of the person with disabilities)
  - Peer and community relationships (Community members understand and acknowledge that individuals with disabilities are capable of having meaningful relationships, people with disabilities marry and have children)
  - Violence and abuse (Protection from violence; collaboration between all relevant stakeholders to address the issue of violence).

Appendix 4

1) full search strategy strategiesWeb of Science (Clarivate):
   Initial search date: 08.04.20264
   Update search date: 20.03.2026

Search strategy:

1: TS=((developed or “high* developed” or “high income”) NEAR (countr* or nation? or population? or world or state*))

2: TS = ((Aruba or Andorra or "United Arab Emirates" or "American Samoa" or "Antigua and Barbuda*" or Australia or Austria or Belgium or Bahrain or "The Bahamas*" or Bermuda or Barbados or "Brunei Darussalam" or Canada or Switzerland or "Channel Island" or Chile or Curacao or "Cayman Islands" or Cyprus or Czechia* or Germany or Denmark or Spain or Estonia or Finland or France or "Faroe* Islands" or (“United Kingdom” or “England” or “Wales” or “Scotland” or “North Ireland”) or Gibraltar or Greece or Greenland or Guam or Guyana or ("Hong Kong" or "Hong Kong SAR" or "Hong Kong SAR, China") or Croatia or Hungary or "Isle of Man" or Ireland or Iceland or Israel or Italy or Japan or "St. Kitts and Nevis" or ("Korea" or "Rep. Korea" or "South Korea") or Kuwait or Lichtenstein or Lithuania or Luxembourg or Latvia or ("Macao" or "Macao SAR, China") or ("St. Martin French* part" or "French St Martin") or Monaco or Malta or "Northern Mariana Islands" or "New Caledonia" or Netherlands or Norway or Nauru or "New Zealand" or Oman or Panama or Poland or "Puerto Rico" or Portugal or "French Polynesia" or Qatar or Romania or "Saudi Arabia" or Singapore or "San arino" or ("Slovakia" or "Slovak Republic") or Slovenia or Sweden or ("Sint Maarten" or "Sint Maarten”) or Seychelles or "Turks and Caicos Islands" or "Trinidad and Tobago" or ("Taiwan" or "Taiwan, China") or Uruguay or "United States" or "British Virgin Islands" or ("Virgin Islands U.S." or "Virgin Islands" or "American Virgin Islands") or (Venezuela or "Venezuela, RB")) NOT (“African-American*“ or “African-American*“ or “Mexican American*“ or “American Indian*“ or “Asian American*“ or “native American*“))

3: TS=((developed or “high* developed” or "high income”) NEAR (econom*))

4: TS = (high NEAR/3 countr*)

5: #1 OR #2 OR #3 OR #4

6: TS=(disabili* OR disable* OR handicap* OR deficien* OR impair*)

7: TI=(physical* NEAR/5 (impair* or deficien* or disable* or disabili* or handicap*))

8: TI=((“cerebral pals*“ or “spina bifida” or “muscular dystroph*“ or arthriti* or “osteogenesis imperfecta” or “musculoskeletal abnormalit*“ or “musculo-skeletal abnormalit*“ or “muscular abnormalit*“ or “skeletal abnormalit*“ or “limb abnormalit*“ or “brain injur*“ or amput* or clubfoot or polio* or paraplegi* or paralys* or paralyz* or hemiplegi* or stroke* or “cerebrovascular accident*“) NEAR/2 (impair* or disabilit* or disabl* or handicap*))

9: TI=(((visual* or vision or eye* or ocular) NEAR/5 (loss* or impair* or deficien* or disable* or disabili* or handicap*)) or blind*)

10: TI=(autis* or dyslexi* or “Down* syndrome” or mongolism or “trisomy 21”)

11: TI=(((hearing or acoustic or ear*) NEAR/5 (loss* or impair* or deficien* or disable* or disabili* or handicap*)) or deaf*)

12: TI=((intellectual* or educational* or mental* or psychological* or developmental) NEAR/5 (impair* or retard* or deficien* or disable* or disabili* or handicap* or ill*))

13: TI=((mental* or emotional* or psychiatric or neurologic*) NEAR/2 (disorder* or ill or illness*))

14: TI=((schizophreni* or psychos* or psychotic or schizoaffective or schizophreniform or dementia* or alzheimer*) NEAR/2 (impair* or disabilit* or disabl* or handicap*))

15: TS=((depression or depressive or anxiety or psychiat* or well-being or “quality of life” or self-esteem or “self perception”) NEAR/2 (impair* or disabilit* or disabl* or handicap*) NEAR (severe or chronic or major))

16: TI=((mental* or emotional* or psychiatric or neurologic*) NEAR/2 (disorder* or ill or illness*) NEAR (severe or chronic or major))

17: TI=((communication or language or speech or learning) NEAR/5 disorder*)

18: TI=((cognitive* or learning or mobility or sensory or visual* or vision or sight or hearing or physical* or mental* or intellectual*) NEAR/2 (impair* or disabilit* or disabl* or handicap*))

19: TI=((disable* or disabilit* or handicapped) NEAR/5 (person* or people or child* or adolescen* or women or mother* or maternal or father* or men or parents or group*))

20: TI=((physical* or intellectual* or learning or psychiatric* or sensory or motor or neuromotor or cognitive or mental* or developmental or communication or learning) NEAR/2 (disabilit* or disabl* or handicap*))

21: TS = (intervention$ or *promoting* or *enhancing* or *policy)

22: TS=(("social inclusion" OR "social participation" OR "social interaction" OR "social integration" OR "community participation" OR "community involvement" OR "social network$" OR "community inclusion" OR “social capital”))

23: #6 OR #7 OR #8 OR #12 OR #10 OR #9 OR #11 OR #18 OR #17 OR #16 OR #15 OR #14 OR #13 OR #19 OR #20

24: #23 AND #21 AND #22 AND #5

Limits / filters:
None. No restrictions on language or publication date.

Last search: 20.03.2026

2) Scopus (Elsevier)
   Initial search date: 16.04.2024
   Update search date: 20.03.2026

Search strategy:

((TITLE-ABS-KEY ("social inclusion" OR "social participation" OR "social interaction" OR "social integration" OR "community participation" OR "community involvement" OR "social network$" OR "community inclusion" OR “social capital”)) AND ((TITLE ((disabili* OR disable* OR handicap* OR deficien* OR impair*) OR (developmental* W/5 (impair* OR deficien* OR disable* OR disabili* OR handicap*)))) OR (TITLE (("cerebral pals*" OR "spina bifida" OR "muscular dystroph*" OR arthriti* OR "osteogenesis imperfecta" OR "musculoskeletal abnormalit*" OR "musculo-skeletal abnormalit*" OR "muscular abnormalit*" OR "skeletal abnormalit*" OR "limb abnormalit*" OR "brain injur*" OR amput* OR clubfoot OR polio* OR paraplegi* OR paralys* OR paralyz* OR hemiplegi* OR stroke* OR "cerebrovascular accident*") W/2 (impair* OR disabilit* OR disabl* OR handicap*))) OR (TITLE (((visual* OR vision OR eye* OR ocular) W/5 (loss* OR impair* OR deficien* OR disable* OR disabili* OR handicap*)) OR blind*)) OR (TITLE (((hearing OR acoustic OR ear*) W/5 (loss* OR impair* OR deficien* OR disable* OR disabili* OR handicap*)) OR deaf*)) OR (TITLE ((intellectual* OR educational* OR mental* OR psychological* OR developmental) W/5 (impair* OR retard* OR deficien* OR disable* OR disabili* OR handicap* OR ill*))) OR (TITLE (autis* OR dyslexi* OR "Down* syndrome" OR mongolism OR "trisomy 21")) OR (TITLE ((mental* OR emotional* OR psychiatric OR neurologic*) W/2 (disorder* OR ill OR illness*))) OR (TITLE ((mental* OR emotional* OR psychiatric OR neurologic*) W/2 (disorder* OR ill OR illness*))) OR (TITLE ((schizophreni* OR psychos* OR psychotic OR schizoaffective OR schizophreniform OR dementia* OR alzheimer*) W/2 (impair* OR disabilit* OR disabl* OR handicap*))) OR (TITLE-ABS-KEY ((depression OR depressive OR anxiety OR psychiat* OR well-being OR "quality of life" OR self-esteem OR "self perception") W/2 (impair* OR disabilit* OR disabl* OR handicap*) W/1 (severe OR chronic OR major))) OR (TITLE ((mental* OR emotional* OR psychiatric OR neurologic*) W/2 (disorder* OR ill OR illness*) W/1 (severe OR chronic OR major))) OR (TITLE ((schizophreni* OR psychos* OR psychotic OR schizoaffective OR schizophreniform OR dementia* OR alzheimer*) W/2 (impair* OR disabilit* OR disabl* OR handicap*))) OR (TITLE-ABS-KEY ((depression OR depressive OR anxiety OR psychiat* OR well-being OR "quality of life" OR self-esteem OR "self perception") W/2 (impair* OR disabilit* OR disabl* OR handicap*) W/1 (severe OR chronic))) OR (TITLE ((mental* OR emotional* OR psychiatric OR neurologic*) W/2 (disorder* OR ill OR illness*) W/1 (severe OR chronic))) OR (TITLE ((communication OR language OR speech OR learning) W/5 disorder*)) OR (TITLE ((cognitive* OR learning OR mobility OR sensory OR visual* OR vision OR sight OR hearing OR developmental* OR mental* OR intellectual*) W/2 (impair* OR disabilit* OR disabl* OR handicap*))) OR (TITLE ((developmental* OR intellectual* OR learning OR psychiatric* OR sensory OR motor OR neuromotor OR cognitive OR mental* OR developmental OR communication OR learning) W/2 (disabilit* OR disabl* OR handicap*))) OR (TITLE ((disable* OR disabilit* OR handicapped) W/5 (person* OR people OR child* OR adolescen* OR women OR mother* OR maternal OR men OR father* OR parents OR group*)))) AND (TITLE-ABS-KEY (intervention$ OR *promoting* OR *enhancing* OR *policy*)) AND ((TITLE-ABS-KEY (high W/3 countr*)) OR (TITLE-ABS-KEY ((developed OR "high* developed" OR "high income") W/1 (econom*))) OR (TITLE-ABS-KEY (aruba OR andorra OR "United Arab Emirates" OR "American Samoa" OR "Antigua and Barbuda*" OR australia OR austria OR belgium OR bahrain OR "The Bahamas*" OR bermuda OR barbados OR "brunei darussalam" OR canada OR switzerland OR "channel island" OR chile OR curaçao OR "Cayman Islands" OR cyprus OR czechia* OR germany OR denmark OR spain OR estonia OR finland OR france OR "Faroe* Islands" OR (“united kingdom” OR “england” OR “wales” OR “scotland” OR “north ireland”) OR gibraltar OR greece OR greenland OR guam OR guyana OR ("Hong Kong" OR "Hong Kong SAR" OR "Hong Kong SAR, China") OR croatia OR hungary OR "Isle of Man" OR ireland OR iceland OR israel OR italy OR japan OR "St. Kitts and Nevis" OR (korea OR "Rep. Korea" OR "South Korea") OR kuwait OR lichtenstein OR lithuania OR

luxembourg OR latvia OR (macao OR "Macao SAR, China") OR ("St. Martin French* part" OR "French St Martin") OR monaco OR malta OR "Northern Mariana Islands" OR "New Caledonia" OR netherlands OR norway OR nauru OR "New Zealand" OR oman OR panama OR poland OR "Puerto Rico" OR portugal OR "French Polynesia" OR qatar OR romania OR "Saudi Arabia" OR singapore OR "San Marino" OR ("Slovakia" OR "Slovak Republic") OR slovenia OR sweden OR ("Sint Maarten" OR "Sint Maarten (Duch part)") OR seychelles OR "Turks and Caicos Islands" OR "Trinidad and Tabago" OR ("Taiwan" OR "Taiwan, China") OR uruguay OR "United States" OR "British Virgin Islands" OR ("Virgin Islands U.S." OR "Virgin Islands" OR "American Virgin Islands") OR (venezuela OR "Venezuela, RB") AND NOT ("African-American*" OR "African-American*" OR "Mexican American*" OR "American Indian*" OR "Asian American*" OR "native American*"))) OR (TITLE-ABS-KEY ((developed OR "high* developed" OR "high income") W/1 (countr* OR nation? OR population? OR world OR state*)))))

Limits / filters:
None. No restrictions on language or publication date.

Last search: 20.03.2026

3) PubMed (NCBI)
   Initial search date: 15.04.2024
   Update search date: 20.03.2026

Search Strategy:

((("social inclusion"[Title/Abstract] OR "social participation"[Title/Abstract] OR "social interaction"[Title/Abstract] OR "social integration"[Title/Abstract] OR "community participation"[Title/Abstract] OR "community involvement"[Title/Abstract] OR "social network"[Title/Abstract] OR "community inclusion"[Title/Abstract] OR "social capital"[Title/Abstract]) AND ((((((((((((("disabili*"[Title/Abstract] OR "disable*"[Title/Abstract] OR "handicap*"[Title/Abstract] OR "deficien*"[Title/Abstract] OR "impair*"[Title/Abstract]) OR ("physical impairment"[Title:~5] OR "physical deficient"[Title:~5] OR "physical deficiency"[Title:~5] OR "physical disabled"[Title:~5] OR "physical disability"[Title:~5] OR "physical disabilities"[Title:~5] OR "physical handicap"[Title:~5] OR "physical handicapped"[Title:~5])) OR ("cerebral palsy impairment "[Title: ~2] OR "cerebral palsy disability " [Title: ~2] OR "cerebral palsy disabilities "[Title: ~2] OR "cerebral palsy disabled "[Title: ~2] OR "cerebral palsy handicap"[Title: ~2] OR "cerebral palsy handicapped "[Title: ~2] OR "spinal bifida impairment "[Title: ~2] OR "spina bifida disability " [Title: ~2] OR "spina bifida disabilities "[Title: ~2] OR "spina bifida disabled "[Title: ~2] OR "spina bifida handicap"[Title: ~2] OR "spina bifida handicapped "[Title: ~2] OR "muscular dystrophy impairment "[Title: ~2] OR "muscular dystrophy disability " [Title: ~2] OR "muscular dystrophy disabilities "[Title: ~2] OR "muscular dystrophy disabled "[Title: ~2] OR "muscular dystrophy handicap"[Title: ~2] OR "muscular dystrophy handicapped "[Title: ~2] OR "muscular dystrophies impairment "[Title: ~2] OR "muscular dystrophies disability " [Title: ~2] OR "muscular dystrophies disabilities "[Title: ~2] OR "muscular dystrophies disabled "[Title: ~2] OR "muscular dystrophies handicap"[Title: ~2] OR "muscular dystrophies handicapped "[Title: ~2] OR "arthritis impairment "[Title: ~2] OR "arthritis disability " [Title: ~2] OR "arthritis disabilities "[Title: ~2] OR "arthritis disabled "[Title: ~2] OR "arthritis handicap"[Title: ~2] OR "arthritis handicapped "[Title: ~2] OR "osteogenesis imperfecta impairment "[Title: ~2] OR "osteogenesis imperfecta disability " [Title: ~2] OR "osteogenesis imperfecta disabilities "[Title: ~2] OR "osteogenesis imperfecta disabled "[Title: ~2] OR "osteogenesis imperfecta handicap"[Title: ~2] OR "osteogenesis imperfecta handicapped "[Title: ~2] OR "musculoskeletal

abnormality impairment "[Title: ~2] OR "musculoskeletal abnormality disability " [Title: ~2] OR "musculoskeletal abnormality disabilities" [Title: ~2] OR "musculoskeletal abnormality disabled "[Title: ~2] OR "musculoskeletal abnormality handicap"[Title: ~2]OR "musculoskeletal abnormality handicapped "[Title: ~2] OR "musculo-skeletal abnormality impairment "[Title: ~2] OR "musculo-skeletal abnormality disability " [Title: ~2] OR "musculo-skeletal abnormality disabilities "[Title: ~2] OR "musculo-skeletal abnormality disabled "[Title: ~2] OR "musculo-skeletal abnormality handicap"[Title: ~2] OR "musculo-skeletal abnormality handicapped "[Title: ~2] OR "muscular abnormality impairment "[Title: ~2] OR "muscular abnormality disability " [Title: ~2] OR "muscular abnormality disabilities "[Title: ~2] OR "muscular abnormality disabled "[Title: ~2] OR "muscular abnormality handicap"[Title: ~2] OR "muscular abnormality handicapped "[Title: ~2] OR "skeletal abnormality impairment "[Title: ~2] OR "skeletal abnormality disability " [Title: ~2] OR "skeletal abnormality disabilities "[Title: ~2] OR "skeletal abnormality disabled "[Title: ~2] OR "skeletal abnormality handicap"[Title: ~2] OR "skeletal abnormality handicapped "[Title: ~2] OR "limb abnormality impairment "[Title: ~2] OR "limb abnormality disability " [Title: ~2] OR "limb abnormality disabilities "[Title: ~2] OR "limb abnormality disabled "[Title: ~2] OR "limb abnormality handicap"[Title: ~2] OR "limb abnormality handicapped "[Title: ~2] OR "brain injury impairment "[Title: ~2] OR "brain injury disability " [Title: ~2] OR "brain injury disabilities "[Title: ~2] OR "brain injury disabled "[Title: ~2] OR "brain injury handicap"[Title: ~2] OR "brain injury handicapped "[Title: ~2] OR "amputation impairment "[Title: ~2] OR "amputation disability " [Title: ~2] OR "amputation disabilities "[Title: ~2] OR "amputation disabled "[Title: ~2] OR "amputation handicap"[Title: ~2] OR "amputation handicapped "[Title: ~2] OR "clubfoot impairment "[Title: ~2] OR "clubfoot disability " [Title: ~2] OR "clubfoot disabilities "[Title: ~2] OR "clubfoot disabled "[Title: ~2] OR "clubfoot handicap"[Title: ~2] OR "clubfoot handicapped "[Title: ~2] "Poliomyelitis impairment "[Title: ~2] OR "Poliomyelitis disability " [Title: ~2] OR "Poliomyelitis disabilities "[Title: ~2] OR "Poliomyelitis disabled "[Title: ~2] OR "Poliomyelitis handicap"[Title: ~2] OR "Poliomyelitis handicapped "[Title: ~2] OR "paraplegic impairment "[Title: ~2] OR "paraplegic disability " [Title: ~2] OR "paraplegic disabilities "[Title: ~2] OR "paraplegic disabled "[Title: ~2] OR "paraplegic handicap"[Title: ~2] OR "paraplegic handicapped "[Title: ~2] OR "paraplegia impairment "[Title: ~2] OR "paraplegia disability " [Title: ~2] OR "paraplegia disabilities "[Title: ~2] OR "paraplegia disabled "[Title: ~2] OR "paraplegia handicap"[Title: ~2] OR "paraplegia handicapped "[Title: ~2] OR "paralysis impairment "[Title: ~2] OR "paralysis disability " [Title: ~2] OR "paralysis disabilities "[Title: ~2] OR "paralysis disabled "[Title: ~2] OR "paralysis handicap"[Title: ~2]OR "paralysis handicapped "[Title: ~2] OR "paralyze impairment "[Title: ~2] OR "paralyze disability " [Title: ~2] OR "paralyze disabilities "[Title: ~2] OR"paralyze disabled "[Title: ~2] OR "paralyze handicap"[Title: ~2] OR "paralyze handicapped "[Title: ~2] OR "paralyzed impairment "[Title: ~2] OR "paralyzed disability " [Title: ~2] OR "paralyzed disabilities" [Title: ~2] OR "paralyzed disabled "[Title: ~2] OR "paralyzed handicap"[Title: ~2] OR "paralyzed handicapped "[Title: ~2] OR "hemiplegia impairment "[Title: ~2] OR "hemiplegia disability " [Title: ~2] OR "hemiplegia disabilities "[Title: ~2] OR "hemiplegia disabled "[Title: ~2] OR "hemiplegia handicap"[Title: ~2] OR "hemiplegia handicapped "[Title: ~2] OR "stroke impairment "[Title: ~2] OR "stroke disability " [Title: ~2] OR "stroke disabilities "[Title: ~2] OR "stroke disabled "[Title: ~2] OR "stroke handicap"[Title: ~2] OR "stroke handicapped "[Title: ~2] OR "cerebrovascular accident impairment "[Title: ~2] OR "cerebrovascular accident disability " [Title: ~2] OR "cerebrovascular accident disabilities "[Title: ~2] OR "cerebrovascular accident disabled "[Title: ~2] OR "cerebrovascular accident handicap"[Title: ~2] OR "cerebrovascular accident handicapped "[Title: ~2])) OR ("visual loss" [Title: ~5] OR "visual impairment" [Title:~5] OR "visual deficiency" [Title: ~5] OR "visual deficient" [Title: ~5] OR "visual disabled" [Title: ~5] OR "visual disable" [Title: ~5] OR "visual disability" [Title: ~5] OR "visual disabilities" [Title: ~5] OR "visual handicap" [Title: ~5] OR "visual handicapped" [Title: ~5] OR "eye loss" [Title: ~5] OR "eye impairment" [Title: ~5] OR "eye deficiency" [Title: ~5] OR "eye deficient" [Title: ~5] OR "eye disabled" [Title: ~5] OR "eye disable" [Title: ~5] OR "eye disability" [Title: ~5] OR "eye disabilities" [Title: ~5] OR "eye handicap" [Title: ~5] OR "eye handicapped" [Title: ~5] OR "occular loss" [Title: ~5] OR

"occular impairment" [Title: ~5] OR "occular deficiency" [Title: ~5] OR "occular deficient" [Title: ~5] OR "occular disabled" [Title: ~5] OR "occular disable" [Title: ~5] OR "occular disability" [Title: ~5] OR "occular disabilities" [Title: ~5] OR "occular handicap" [Title: ~5] OR "occular handicapped" [Title: ~5] OR blind*)) OR ("hearing loss" [Title: ~5] OR "hearing impairment" [Title:~5] OR "hearing deficiency" [Title: ~5] OR "hearing deficient" [Title: ~5] OR "hearing disabled" [Title: ~5] OR "hearing disable" [Title: ~5] OR "hearing disability" [Title: ~5] OR "hearing disabilities" [Title: ~5] OR "hearing handicap" [Title: ~5] OR "hearing handicapped" [Title: ~5] OR "acoustic loss" [Title: ~5] OR "acoustic impairment" [Title:~5] OR "acoustic deficiency" [Title: ~5] OR "acoustic deficient" [Title: ~5] OR "acoustic disabled" [Title: ~5] OR "acoustic disable" [Title: ~5] OR "acoustic disability" [Title: ~5] OR "acoustic disabilities" [Title: ~5] OR "acoustic handicap" [Title: ~5] OR "acoustic handicapped" [Title: ~5] OR "visual loss" [Title: ~5] OR "visual impairment" [Title:~5] OR "visual deficiency" [Title: ~5] OR "visual deficient" [Title: ~5] OR "visual disabled" [Title: ~5] OR "visual disable" [Title: ~5] OR "visual disability" [Title: ~5] OR "visual disabilities" [Title: ~5] OR "visual handicap" [Title: ~5] OR "visual handicapped" [Title: ~5] OR deaf*)) OR ("intellectual impairment" [Title:~5] OR "intellectual impaired" [Title: ~5] "intellectual retard" [Title: ~5] OR "intellectual retarded " [Title: ~5] OR "intellectual retardation" [Title: ~5] OR "intellectual deficiency" [Title: ~5] OR "intellectual deficient" [Title: ~5] OR "intellectual disabled" [Title: ~5] OR "intellectual disable" [Title: ~5] OR "intellectual disability" [Title: ~5] OR "intellectual disabilities" [Title: ~5] OR "intellectual handicap" [Title: ~5] OR "intellectual handicapped" [Title: ~5] OR "intellectual ill" [Title: ~5] OR "intellectual illness" [Title: ~5] OR "educational impairment" [Title:~5] OR "educational impaired" [Title: ~5] "educational retard" [Title: ~5] OR "educational retarded " [Title: ~5] OR "educational retardation" [Title: ~5] OR "educational deficiency" [Title: ~5] OR "educational deficient" [Title: ~5] OR "educational disabled" [Title: ~5] OR "educational disable" [Title: ~5] OR "educational disability" [Title: ~5] OR "educational disabilities" [Title: ~5] OR "educational handicap" [Title: ~5] OR "educational handicapped" [Title: ~5] OR "educational ill" [Title: ~5] OR "educational illness" [Title: ~5] OR "mental impairment" [Title:~5] OR "mental impaired" [Title: ~5] "mental retard" [Title: ~5] OR "mental retarded " [Title: ~5] OR "mental retardation" [Title: ~5] OR "mental deficiency" [Title: ~5] OR "mental deficient" [Title: ~5] OR "mental disabled" [Title: ~5] OR "mental disable" [Title: ~5] OR "mental disability" [Title: ~5] OR "mental disabilities" [Title: ~5] OR "mental handicap" [Title: ~5] OR "mental handicapped" [Title: ~5] OR "mental ill" [Title: ~5] OR "mental illness" [Title: ~5] OR "mentally impairment" [Title:~5] OR "mentally impaired" [Title: ~5] "mentally retard" [Title: ~5] OR "mentally retarded " [Title: ~5] OR "mentally retardation" [Title: ~5] OR "mentally deficiency" [Title: ~5] OR "mentally deficient" [Title: ~5] OR "mentally disabled" [Title: ~5] OR "mentally disable" [Title: ~5] OR "mentally disability" [Title: ~5] OR "mentally disabilities" [Title: ~5] OR "mentally handicap" [Title: ~5] OR "mentally handicapped" [Title: ~5] OR "mentally ill" [Title: ~5] OR "mentally illness" [Title: ~5] OR "psychological impairment" [Title:~5] OR "psychological impaired" [Title: ~5] "psychological retard" [Title: ~5] OR "psychological retarded " [Title: ~5] OR "psychological retardation" [Title: ~5] OR "psychological deficiency" Title: ~5] OR "psychological deficient" [Title: ~5] OR "psychological disabled" [Title: ~5] OR "psychological disable" [Title: ~5] OR "psychological disability" [Title: ~5] OR "psychological disabilities" [Title: ~5] OR "psychological handicap" [Title: ~5] OR "psychological handicapped" [Title: ~5] OR "psychological ill" [Title: ~5] OR "psychological illness" [Title: ~5] OR "psychologically impairment" [Title:~5] OR "psychologically impaired" [Title: ~5] "psychologically retard" [Title: ~5] OR "psychologically retarded " [Title: ~5] OR "psychologically retardation" [Title: ~5] OR "psychologically deficiency" [Title: ~5] OR "psychologically deficient" [Title: ~5] OR "psychologically disabled" [Title: ~5] OR "psychologically disable" [Title: ~5] OR "psychologically disability" [Title: ~5] OR "psychologically disabilities" [Title: ~5] OR "psychologically handicap" [Title: ~5] OR "psychologically handicapped" [Title: ~5] OR "psychologically ill" [Title: ~5] OR "psychologically illness" [Title: ~5] OR "developmental impairment" [Title:~5] OR "developmental impaired" [Title: ~5] "developmental retard" [Title: ~5] OR "developmental retarded " [Title: ~5] OR "developmental retardation" [Title: ~5] OR "developmental deficiency" [Title: ~5] OR "developmental deficient" [Title: ~5]

OR "developmental disabled" [Title: ~5] OR "developmental disable" [Title: ~5] OR "developmental disability" [Title: ~5] OR "developmental disabilities" [Title: ~5] OR "developmental handicap" [Title: ~5] OR "developmental handicapped" [Title: ~5] OR "developmental ill" [Title: ~5] OR "visual illness" [Title: ~5])) OR (autis*[Title] OR dyslexi*[Title] OR "Down* syndrome"[Title] OR mongolism[Title] OR "trisomy 21"[Title])) OR ("mental disorder" [Title: ~2] OR "mental disorders" [Title: ~2] OR "mental ill" [Title: ~2] OR "mental illness" [Title: ~2] OR "mental illnesses" [Title: ~2] OR "mentally disorder" [Title: ~2] OR "mentally disorders" [Title: ~2] OR "mentally ill" [Title: ~2] OR "mentally illness" [Title: ~2] OR "mentally illnesses" [Title: ~2] OR "emotional disorder" [Title: ~2] OR "emotional disorders" [Title: ~2] OR "emotional ill" [Title: ~2] OR "emotional illness" [Title: ~2] OR "emotional illnesses" [Title: ~2] OR "emotionally disorder" [Title: ~2] OR "emotionally disorders" [Title: ~2] OR "emotionally ill" [Title: ~2] OR "emotionally illness" [Title: ~2] OR "emotionally illnesses" [Title: ~2] OR "psychiatric disorder" [Title: ~2] OR "psychiatric disorders" [Title: ~2] OR "psychiatric ill" [Title: ~2] OR "psychiatric illness" [Title: ~2] OR "psychiatric illnesses" [Title: ~2] OR "neurological disorder" [Title: ~2] OR "neurological disorders" [Title: ~2] OR "neurological ill" [Title: ~2] OR "neurological illness" [Title: ~2] OR "neurological illnesses" [Title: ~2])) OR ("schizophrenia impairment" [Title:~2] OR "schizophrenia impaired" [Title: ~2] OR "schizophrenia disabled" [Title: ~2] OR "schizophrenia disable" [Title: ~2] OR "schizophrenia disability" [Title: ~2] OR "schizophrenia disabilities" [Title: ~2] OR "schizophrenia handicap" [Title: ~2] OR "schizophrenia handicapped" [Title: ~2] OR "schizophrenics impairment" [Title:~2] OR "schizophrenics impaired" [Title: ~2] OR "schizophrenics disabled" [Title: ~2] OR "schizophrenics disable" [Title: ~2] OR "schizophrenics disability" [Title: ~2] OR "schizophrenics disabilities" [Title: ~2] OR "schizophrenics handicap" [Title: ~2] OR "schizophrenics handicapped" [Title: ~2] OR "psycho impairment" [Title:~2] OR "psycho impaired" [Title: ~2] OR "psycho disabled" [Title: ~2] OR "psycho disable" [Title: ~2] OR "psycho disability" [Title: ~2] OR "psycho disabilities" [Title: ~2] OR "psycho handicap" [Title: ~2] OR "psycho handicapped" [Title: ~2] OR "psychos impairment" [Title:~2] OR "psychos impaired" [Title: ~2] OR "psychos disabled" [Title: ~2] OR "psychos disable" [Title: ~2] OR "psychos disability" [Title: ~2] OR "psychos disabilities" [Title: ~2] OR "psychos handicap" [Title: ~2] OR "psychos handicapped" [Title: ~2] OR "psychotric impairment" [Title:~2] OR "psychotric impaired" [Title: ~2] OR "psychotric disabled" [Title: ~2] OR "psychotric disable" [Title: ~2] OR "psychotric disability" [Title: ~2] OR "psychotric disabilities" [Title: ~2] OR "psychotric handicap" [Title: ~2] OR "psychotric handicapped" [Title: ~2] OR "schizoaffective impairment" [Title:~2] OR "schizoaffective impaired" [Title: ~2] OR "schizoaffective disabled" [Title: ~2] OR "schizoaffective disable" [Title: ~2] OR "schizoaffective disability" [Title: ~2] OR "schizoaffective disabilities" [Title: ~2] OR "schizoaffective handicap" [Title: ~2] OR "schizoaffective handicapped" [Title: ~2] OR "schizophreniform impairment" [Title:~2] OR "schizophreniform impaired" [Title: ~2] OR "schizophreniform disabled" [Title: ~2] OR "schizophreniform disable" [Title: ~2] OR "schizophreniform disability" [Title: ~2] OR "schizophreniform disabilities" [Title: ~2] OR "schizophreniform handicap" [Title: ~2] OR "schizophreniform handicapped" [Title: ~2] OR "dementia impairment" [Title:~2] OR "dementia impaired" [Title: ~2] OR "dementia disabled" [Title: ~2] OR "dementia disable" [Title: ~2] OR "dementia disability" [Title: ~2] OR "dementia disabilities" [Title: ~2] OR "dementia handicap" [Title: ~2] OR "dementia handicapped" [Title: ~2] OR "alzheimer impairment" [Title:~2] OR "alzheimer impaired" [Title: ~2] OR "alzheimer disabled" [Title: ~2] OR "alzheimer disable" [Title: ~2] OR "alzheimer disability" [Title: ~2] OR "alzheimer disabilities" [Title: ~2] OR "alzheimer handicap" [Title: ~2] OR "alzheimer handicapped" [Title: ~2] OR "alzheimer’s impairment" [Title:~2] OR "alzheimer’s impaired" [Title: ~2] OR "alzheimer’s disabled" [Title: ~2] OR "alzheimer’s disable" [Title: ~2] OR "alzheimer’s disability" [Title: ~2] OR "alzheimer’s disabilities" [Title: ~2] OR "alzheimer’s handicap" [Title: ~2] OR "alzheimer’s handicapped" [Title: ~2])) OR ("severe depression impairment" [Title:~2] OR "severe depression impaired" [Title: ~2] OR "severe depression disabled" [Title: ~2] OR "severe depression disable" [Title: ~2] OR "severe depression disability" [Title: ~2] OR "severe depression disabilities" [Title: ~2] OR "severe depression handicap" [Title: ~2] OR "severe depression handicapped" [Title: ~2] OR "chronic depression impairment" [Title:~2]

OR "chronic depression impaired" [Title: ~2] OR "chronic depression disabled" [Title: ~2] OR "chronic depression disable" [Title: ~2] OR "chronic depression disability" [Title: ~2] OR "chronic depression disabilities" [Title: ~2] OR "chronic depression handicap" [Title: ~2] OR "chronic depression handicapped" [Title: ~2] "major depression impairment" [Title:~2] OR "major depression impaired" [Title: ~2] OR "major depression disabled" [Title: ~2] OR "major depression disable" [Title: ~2] OR "major depression disability" [Title: ~2] OR "major depression disabilities" [Title: ~2] OR "major depression handicap" [Title: ~2] OR "major depression handicapped" [Title: ~2] OR "severe depressive impairment" [Title:~2] OR "severe depressive impaired" [Title: ~2] OR "severe depressive disabled" [Title: ~2] OR "severe depressive disable" [Title: ~2] OR "severe depressive disability" [Title: ~2] OR "severe depressive disabilities" [Title: ~2] OR "severe depressive handicap" [Title: ~2] OR "severe depressive handicapped" [Title: ~2] OR "chronic depressive impairment" [Title:~2] OR "chronic depressive impaired" [Title: ~2] OR "chronic depressive disabled" [Title: ~2] OR "chronic depressive disable" [Title: ~2] OR "chronic depressive disability" [Title: ~2] OR "chronic depressive disabilities" [Title: ~2] OR "chronic depressive handicap" [Title: ~2] OR "chronic depressive handicapped" [Title: ~2] OR "major depressive impairment" [Title:~2] OR "major depressive impaired" [Title: ~2] OR "major depressive disabled" [Title: ~2] OR "major

depressive disable" [Title: ~2] OR "major depressive disability" [Title: ~2] OR "major depressive disabilities" [Title: ~2] OR "major depressive handicap" [Title: ~2] OR "major depressive handicapped" [Title: ~2] OR "severe anxiety impairment" [Title:~2] OR "severe anxiety impaired" [Title: ~2] OR "severe anxiety disabled" [Title: ~2] OR "severe anxiety disable" [Title: ~2] OR "severe anxiety disability" [Title: ~2] OR "severe anxiety disabilities" [Title: ~2] OR "severe anxiety handicap" [Title: ~2] OR "severe anxiety handicapped" [Title: ~2] OR "chronic anxiety impairment" [Title:~2] OR "chronic anxiety impaired" [Title: ~2] OR "chronic anxiety disabled" [Title: ~2] OR "chronic anxiety disable" [Title: ~2] OR "chronic anxiety disability" [Title: ~2] OR "chronic anxiety disabilities" [Title: ~2] OR "chronic anxiety handicap" [Title: ~2] OR "chronic anxiety handicapped" [Title: ~2] OR "major anxiety impairment" [Title:~2] OR "major anxiety impaired" [Title: ~2] OR "major anxiety disabled" [Title: ~2] OR "major anxiety disable" [Title: ~2] OR "major anxiety disability" [Title: ~2] OR "major anxiety disabilities" [Title: ~2] OR "major anxiety handicap" [Title: ~2] OR "major anxiety handicapped" [Title: ~2] OR "severe psychiatric impairment" [Title:~2] OR "severe psychiatric impaired" [Title: ~2] OR "severe psychiatric disabled" [Title: ~2] OR "severe psychiatric disable" [Title: ~2] OR "severe psychiatric disability" [Title: ~2] OR "severe psychiatric disabilities" [Title: ~2] OR "severe psychiatric handicap" [Title: ~2] OR "severe psychiatric handicapped" [Title: ~2] OR "chronic psychiatric impairment" [Title:~2] OR "chronic psychiatric impaired" [Title: ~2] OR "chronic psychiatric disabled" [Title: ~2] OR "chronic psychiatric disable" [Title: ~2] OR "chronic psychiatric disability" [Title: ~2] OR "chronic psychiatric disabilities" [Title: ~2] OR "chronic psychiatric handicap" [Title: ~2] OR "chronic psychiatric handicapped" [Title: ~2] OR"major psychiatric impairment" [Title:~2] OR "major psychiatric impaired" [Title: ~2] OR "major psychiatric disabled" [Title: ~2] OR "major psychiatric disable" [Title: ~2] OR "major psychiatric disability" [Title: ~2] OR "major psychiatric disabilities" [Title: ~2] OR "major psychiatric handicap" [Title: ~2] OR "major psychiatric handicapped" [Title: ~2] OR "severe well-being impairment" [Title:~2] OR "severe well-being impaired" [Title: ~2] OR "severe well-being disabled" [Title: ~2] OR "severe well-being disable" [Title: ~2] OR "severe well-being disability" [Title: ~2] OR "severe well-being disabilities" [Title: ~2] OR "severe well-being handicap" [Title: ~2] OR "severe well-being handicapped" [Title: ~2] OR "chronic well-being impairment" [Title:~2] OR "chronic well-being impaired" [Title: ~2] OR "chronic well-being disabled" [Title: ~2] OR "chronic well-being disable" [Title: ~2] OR "chronic well-being disability" [Title: ~2] OR "chronic well-being disabilities" [Title: ~2] OR "chronic well-being handicap" [Title: ~2] OR "chronic well-being handicapped" [Title: ~2] OR "major well-being impairment" [Title:~2] OR "major well-being impaired" [Title: ~2] OR "major well-being disabled" [Title: ~2] OR "major well-being disable" [Title: ~2] OR "major well-being disability" [Title: ~2] OR "major well-being disabilities" [Title: ~2] OR "major well-being handicap" [Title: ~2] OR "major well-

being handicapped" [Title: ~2] OR "severe qualit of life impairment" [Title:~2] OR "severe qualit of life impaired" [Title: ~2] OR "severe qualit of life disabled" [Title: ~2] OR "severe qualit of life disable" [Title: ~2] OR "severe qualit of life disability" [Title: ~2] OR "severe qualit of life disabilities" [Title: ~2] OR "severe qualit of life handicap" [Title: ~2] OR "severe qualit of life handicapped" [Title: ~2] OR "chronic qualit of life impairment" [Title:~2] OR "chronic qualit of life impaired" [Title: ~2] OR "chronic qualit of life disabled" [Title: ~2] OR "chronic qualit of life disable" [Title: ~2] OR "chronic qualit of life disability" [Title: ~2] OR "chronic qualit of life disabilities" [Title: ~2] OR "chronic qualit of life handicap" [Title: ~2] OR "chronic qualit of life handicapped" [Title: ~2] OR "major qualit of life impairment" [Title:~2] OR "major qualit of life impaired" [Title: ~2] OR "major qualit of life disabled" [Title: ~2] OR "major qualit of life disable" [Title: ~2] OR "major qualit of life disability" [Title: ~2] OR "major qualit of life disabilities" [Title: ~2] OR "major qualit of life handicap" [Title: ~2] OR "major qualit of life handicapped" [Title: ~2] OR "severe self-esteem impairment" [Title:~2] OR "severe self-esteem impaired" [Title: ~2] OR "severe self-esteem disabled" [Title: ~2] OR "severe self-esteem disable" [Title: ~2] OR "severe self-esteem disability" [Title: ~2] OR "severe self-esteem disabilities" [Title: ~2] OR "severe self-esteem handicap" [Title: ~2] OR "severe self-esteem handicapped" [Title: ~2] OR "chronic self-esteem impairment" [Title:~2] OR "chronic self-esteem impaired" [Title: ~2] OR "chronic self-esteem disabled" [Title: ~2] OR "chronic self-esteem disable" [Title: ~2] OR "chronic self-esteem disability" [Title: ~2] OR "chronic self-esteem disabilities" [Title: ~2] OR "chronic self-esteem handicap" [Title: ~2] OR "chronic self-esteem handicapped" [Title: ~2] OR "major self-esteem impairment" [Title:~2] OR "major self-esteem impaired" [Title: ~2] OR "major self-esteem disabled" [Title: ~2] OR "major self-esteem disable" [Title: ~2] OR "major self-esteem disability" [Title: ~2] OR "major self-esteem disabilities" [Title: ~2] OR "major self-esteem handicap" [Title: ~2] OR "major self-esteem handicapped" [Title: ~2] OR "severe self-perception impairment" [Title:~2] OR "severe self-perception impaired" [Title: ~2] OR "severe self-perception disabled" [Title: ~2] OR "severe self-perception disable" [Title: ~2] OR "severe self-perception disability" [Title: ~2] OR "severe self-perception disabilities" [Title: ~2] OR "severe self-perception handicap" [Title: ~2] OR "severe self-perception handicapped" [Title: ~2] OR "chronic self-perception impairment" [Title:~2] OR "chronic self-perception impaired" [Title: ~2] OR "chronic self-perception disabled" [Title: ~2] OR "chronic self-perception disable" [Title: ~2] OR "chronic self-perception disability" [Title: ~2] OR "chronic self-perception disabilities" [Title: ~2] OR "chronic self-perception handicap" [Title: ~2] OR "chronic self-perception handicapped" [Title: ~2] OR "major self-perception impairment" [Title:~2] OR "major self-perception impaired" [Title: ~2] OR "major self-perception disabled" [Title: ~2] OR "major self-perception disable" [Title: ~2] OR "major self-perception disability" [Title: ~2] OR "major self-perception disabilities" [Title: ~2] OR "major self-perception handicap" [Title: ~2] OR "major self-perception handicapped" [Title: ~2])) OR ("severe mental disorder" [Title: ~2] OR "severe mental disorders" [Title: ~2] OR "severe mental ill" [Title: ~2] OR "severe mental illness" [Title: ~2] OR "severe mental illnesses" [Title: ~2] OR "chronic mental disorder" [Title: ~2] OR "chronic mental disorders" [Title: ~2] OR "chronic mental ill" [Title: ~2] OR "chronic mental illness" [Title: ~2] OR "chronic mental illnesses" [Title: ~2] OR "major mental disorder" [Title: ~2] OR "major mental disorders" [Title: ~2] OR "major mental ill" [Title: ~2] OR "major mental illness" [Title: ~2] OR "major mental illnesses" [Title: ~2] OR "severe mentally disorder" [Title: ~2] OR "severe mentally disorders" [Title: ~2] OR "severe mentally ill" [Title: ~2] OR "severe mentally illness" [Title: ~2] OR "severe mentally illnesses" [Title: ~2] OR "chronic mentally disorder" [Title: ~2] OR "chronic mentally disorders" [Title: ~2] OR "chronic mentally ill" [Title: ~2] OR "chronic mentally illness" [Title: ~2] OR "chronic mentally illnesses" [Title: ~2] OR "major mentally disorder" [Title: ~2] OR "major mentally disorders" [Title: ~2] OR "major mentally ill" [Title: ~2] OR "major mentally illness" [Title: ~2] OR "major mentally illnesses" Title: ~2] OR "severe emotional disorder" [Title: ~2] OR "severe emotional disorders" [Title: ~2] OR "severe emotional ill" [Title: ~2] OR "severe emotional illness" [Title: ~2] OR "severe emotional illnesses" [Title: ~2] OR "chronic emotional disorder" [Title: ~2] OR "chronic emotional disorders" [Title: ~2] OR "chronic emotional ill" [Title: ~2] OR "chronic emotional illness" [Title: ~2] OR

"chronic emotional illnesses" [Title: ~2] OR "major emotional disorder" [Title: ~2] OR "major emotional disorders" [Title: ~2] OR "major emotional ill" [Title: ~2] OR "major emotional illness" [Title: ~2] OR "major emotional illnesses" [Title: ~2] OR "severe emotionally disorder" [Title: ~2] OR "severe emotionally disorders" [Title: ~2] OR "severe emotionally ill" [Title: ~2] OR "severe emotionally illness" [Title: ~2] OR "severe emotionally illnesses" [Title: ~2] OR "chronic emotionally disorder" [Title: ~2] OR "chronic emotionally disorders" [Title: ~2] OR "chronic emotionally ill" [Title: ~2] OR "chronic emotionally illness" [Title: ~2] OR "chronic emotionally illnesses" [Title: ~2] OR "major emotionally disorder" [Title: ~2] OR "major emotionally disorders" [Title: ~2] OR "major emotionally ill" [Title: ~2] OR "major emotionally illness" [Title: ~2] OR "major emotionally illnesses" [Title: ~2] OR "severe psychiatric disorder" [Title: ~2] OR "severe psychiatric disorders" [Title: ~2] OR "severe psychiatric ill" [Title: ~2] OR "severe psychiatric illness" [Title: ~2] OR "severe psychiatric illnesses" [Title: ~2] OR "chronic psychiatric disorder" [Title: ~2] OR "chronic psychiatric disorders" [Title: ~2] OR "chronic psychiatric ill" [Title: ~2] OR "chronic psychiatric illness" [Title: ~2] OR "chronic psychiatric illnesses" [Title: ~2] OR "major psychiatric disorder" [Title: ~2] OR "major psychiatric disorders" [Title: ~2] OR "major psychiatric ill" [Title: ~2] OR "major psychiatric illness" [Title: ~2] OR "major psychiatric illnesses" [Title: ~2] OR "severe neurologic disorder" [Title: ~2] OR "severe neurologic disorders" [Title: ~2] OR "severe neurologic ill" [Title: ~2] OR "severe neurologic illness" [Title: ~2] OR "severe neurologic illnesses" [Title: ~2] OR "chronic neurologic disorder" [Title: ~2] OR "chronic neurologic disorders" [Title: ~2] OR "chronic neurologic ill" [Title: ~2] OR "chronic neurologic illness" [Title: ~2] OR "chronic neurologic illnesses" [Title: ~2] OR "major neurologic disorder" [Title: ~2] OR "major neurologic disorders" [Title: ~2] OR "major neurologic ill" [Title: ~2] OR "major neurologic illness" [Title: ~2] OR "major neurologic illnesses" [Title: ~2])) OR ("communication disorder" [Title: ~5] OR "communication disorders" [Title: ~5] OR "language disorder" [Title: ~5] OR "language disorders" [Title: ~5] OR "speech disorder" [Title: ~5] OR "speech disorders" [Title: ~5] OR "learning disorder" [Title: ~5] OR "learning disorders" [Title: ~5])) OR ("cognitive impairment" [Title:~2] OR "cognitive impaired" [Title: ~2] OR "cognitive disabled" [Title: ~2] OR "cognitive disable" [Title: ~2] OR "cognitive disability" [Title: ~2] OR "cognitive disabilities" [Title: ~2] OR "cognitive handicap" [Title: ~2] OR "cognitive handicapped" [Title: ~2] OR "learning impairment" [Title:~2] OR "learning impaired" [Title: ~2] OR "learning disabled" [Title: ~2] OR "learning disable" [Title: ~2] OR "learning disability" [Title: ~2] OR "learning disabilities" [Title: ~2] OR "learning handicap" [Title: ~2] OR "learning handicapped" [Title: ~2] OR "mobility impairment" [Title:~2] OR "mobility impaired" [Title: ~2] OR "mobility disabled" [Title: ~2] OR "mobility disable" [Title: ~2] OR "mobility disability" [Title: ~2] OR "mobility disabilities" [Title: ~2] OR "mobility handicap" [Title: ~2] OR "mobility handicapped" [Title: ~2] OR "sensory impairment" [Title:~2] OR "sensory impaired" [Title: ~2] OR "sensory disabled" [Title: ~2] OR "sensory disable" [Title: ~2] OR "sensory disability" [Title: ~2] OR "sensory disabilities" [Title: ~2] OR "sensory handicap" [Title: ~2] OR "sensory handicapped" [Title: ~2] OR "visual impairment" [Title:~2] OR "visual impaired" [Title: ~2] OR "visual disabled" [Title: ~2] OR "visual disable" [Title: ~2] OR "visual disability" [Title: ~2] OR "visual disabilities" [Title: ~2] OR "visual handicap" [Title: ~2] OR "visual handicapped" [Title: ~2] OR "visually impairment" [Title:~2] OR "visually impaired" [Title: ~2] OR "visually disabled" [Title: ~2] OR "visually disable" [Title: ~2] OR "visually disability" [Title: ~2] OR "visually disabilities" [Title: ~2] OR "visually handicap" [Title: ~2] OR "visually handicapped" [Title: ~2] OR "vision impairment" [Title:~2] OR "vision impaired" [Title: ~2] OR "vision disabled" [Title: ~2] OR "vision disable" [Title: ~2] OR "vision disability" [Title: ~2] OR "vision disabilities" [Title: ~2] OR "vision handicap" [Title: ~2] OR "vision handicapped" [Title: ~2] OR "sight impairment" [Title:~2] OR "sight impaired" [Title: ~2] OR "sight disabled" [Title: ~2] OR "sight disable" [Title: ~2] OR "sight disability" [Title: ~2] OR "sight disabilities" [Title: ~2] OR "sight handicap" [Title: ~2] OR "sight handicapped" [Title: ~2] OR "hearing impairment" [Title:~2] OR "hearing impaired" [Title: ~2] OR "hearing disabled" [Title: ~2] OR "hearing disable" [Title: ~2] OR "hearing disability" [Title: ~2] OR "hearing disabilities" [Title: ~2] OR "hearing handicap" [Title: ~2] OR "hearing handicapped" [Title: ~2] OR "physical impairment" [Title:~2] OR "physical impaired" [Title: ~2] OR

"physical disabled" [Title: ~2] OR "physical disable" [Title: ~2] OR "physical disability" [Title: ~2] OR "physical disabilities" [Title: ~2] OR "physical handicap" [Title: ~2] OR "physical handicapped" [Title: ~2] OR "physically impairment" [Title:~2] OR "physically impaired" [Title: ~2] OR "physically disabled" [Title: ~2] OR "physically disable" [Title: ~2] OR "physically disability" [Title: ~2] OR "physically disabilities" [Title: ~2] OR "physically handicap" [Title: ~2] OR "physically handicapped" [Title: ~2] OR "mental impairment" [Title:~2] OR "mental impaired" [Title: ~2] OR "mental disabled" [Title: ~2] OR "mental disable" [Title: ~2] OR "mental disability" [Title: ~2] OR "mental disabilities" [Title: ~2] OR "mental handicap" [Title: ~2] OR "mental handicapped" [Title: ~2] OR "mentally impairment" [Title:~2] OR "mentally impaired" [Title: ~2] OR "mentally disabled" [Title: ~2] OR "mentally disable" [Title: ~2] OR "mentally disability" [Title: ~2] OR "mentally disabilities" [Title: ~2] OR "mentally handicap" [Title: ~2] OR "mentally handicapped" [Title: ~2] OR "developemental impairment" [Title:~2] OR "developemental impaired" [Title: ~2] OR "developemental disabled" [Title: ~2] OR "developemental disable" [Title: ~2] OR "developemental disability" [Title: ~2] OR "developemental disabilities" [Title: ~2] OR "developemental handicap" [Title: ~2] OR "developemental handicapped" [Title: ~2] OR "communication impairment" [Title:~2] OR "communication impaired" [Title: ~2] OR "communication disabled" [Title: ~2] OR "communication disable" [Title: ~2] OR "communication disability" [Title: ~2] OR "communication disabilities" [Title: ~2] OR "communication handicap" [Title: ~2] OR "communication handicapped" [Title: ~2] OR "learning impairment" [Title:~2] OR "learning impaired" [Title: ~2] OR "learning disabled" [Title: ~2] OR "learning disable" [Title: ~2] OR "learning disability" [Title: ~2] OR "learning disabilities" [Title: ~2] OR "learning handicap" [Title: ~2] OR "learning handicapped" [Title: ~2])) OR ("physical disabled" [Title: ~2] OR "physical disable" [Title: ~2] OR "physical disability" [Title: ~2] OR "physical disabilities" [Title: ~2] OR "physical handicap" [Title: ~2] OR "physical handicapped" [Title: ~2] OR "physically disabled" [Title: ~2] OR "physically disable" [Title: ~2] OR "physically disability" [Title: ~2] OR "physically disabilities" [Title: ~2] OR "physically handicap" [Title: ~2] OR "physically handicapped" [Title: ~2] OR "intellectual disabled" [Title: ~2] OR "intellectual disable" [Title: ~2] OR "intellectual disability" [Title: ~2] OR "intellectual disabilities" [Title: ~2] OR "intellectual handicap" [Title: ~2] OR "intellectual handicapped" [Title: ~2] OR "intellectually disabled" [Title: ~2] OR "intellectually disable" [Title: ~2] OR "intellectually disability" [Title: ~2] OR "intellectually disabilities" [Title: ~2] OR "intellectually handicap" [Title: ~2] OR "intellectually handicapped" [Title: ~2] OR "learning disabled" [Title: ~2] OR "learning disable" [Title: ~2] OR "learning disability" [Title: ~2] OR "learning disabilities" [Title: ~2] OR "learning handicap" [Title: ~2] OR "learning handicapped" [Title: ~2] OR "psychiatric disabled" [Title: ~2] OR "psychiatric disable" [Title: ~2] OR "psychiatric disability" [Title: ~2] OR "psychiatric disabilities" [Title: ~2] OR "psychiatric handicap" [Title: ~2] OR "psychiatric handicapped" [Title: ~2] OR "sensory disabled" [Title: ~2] OR "sensory disable" [Title: ~2] OR "sensory disability" [Title: ~2] OR "sensory disabilities" [Title: ~2] OR "sensory handicap" [Title: ~2] OR "sensory handicapped" [Title: ~2] OR "motor disabled" [Title: ~2] OR "motor disable" [Title: ~2] OR "motor disability" [Title: ~2] OR "motor disabilities" [Title: ~2] OR "motor handicap" [Title: ~2] OR "motor handicapped" [Title: ~2] OR "motor disabled" [Title: ~2] OR "motor disable" [Title: ~2] OR "motor disability" [Title: ~2] OR "motor disabilities" [Title: ~2] OR "motor handicap" [Title: ~2] OR "motor handicapped" [Title: ~2] OR "neuromotor disabled" [Title: ~2] OR "neuromotor disable" [Title: ~2] OR "neuromotor disability" [Title: ~2] OR "neuromotor disabilities" [Title: ~2] OR "neuromotor handicap" [Title: ~2] OR "neuromotor handicapped" [Title: ~2] OR "cognitive disabled" [Title: ~2] OR "cognitive disable" [Title: ~2] OR "cognitive disability" [Title: ~2] OR "cognitive disabilities" [Title: ~2] OR "cognitive handicap" [Title: ~2] OR "cognitive handicapped" [Title: ~2] OR "mental disabled" [Title: ~2] OR "mental disable" [Title: ~2] OR "mental disability" [Title: ~2] OR "mental disabilities" [Title: ~2] OR "mental handicap" [Title: ~2] OR "mental handicapped" [Title: ~2] OR "mentally disabled" [Title: ~2] OR "mentally disable" [Title: ~2] OR "mentally disability" [Title: ~2] OR "mentally disabilities" [Title: ~2] OR "mentally handicap" [Title: ~2] OR "mentally handicapped" [Title: ~2] OR "developmental disabled" [Title: ~2] OR "developmental disable" [Title: ~2] OR "developmental disability" [Title: ~2] OR "developmental

disabilities" [Title: ~2] OR "developmental handicap" [Title: ~2] OR "developmental handicapped" [Title: ~2] OR "communication disabled" [Title: ~2] OR "communication disable" [Title: ~2] OR "communication disability" [Title: ~2] OR "communication disabilities" [Title: ~2] OR "communication handicap" [Title: ~2] OR "communication handicapped" [Title: ~2] OR "learning disabled" [Title: ~2] OR "learning disable" [Title: ~2] OR "learning disability" [Title: ~2] OR "learning disabilities" [Title: ~2] OR "learning handicap" [Title: ~2] OR "learning handicapped" [Title: ~2])) OR ("disabled person" [Title: ~5] OR "disable person" [Title: ~5] OR "disability person" [Title: ~5] OR "disabilities person" [Title: ~5] OR "handicapped person" [Title: ~5] OR "disabled persons" [Title: ~5] OR "disable persons" [Title: ~5] OR "disability persons" [Title: ~5] OR "disabilities persons" [Title: ~5] OR "handicapped persons" [Title: ~5] OR "disabled people" [Title: ~5] OR "disable people" [Title: ~5] OR "disability people" [Title: ~5] OR "disabilities people" [Title: ~5] OR "handicapped people" [Title: ~5] OR "disabled child" [Title: ~5] OR "disable child" [Title: ~5] OR "disability child" [Title: ~5] OR "disabilities child" [Title: ~5] OR "handicapped child" [Title: ~5] OR "disabled children" [Title: ~5] OR "disable children" [Title: ~5] OR "disability children" [Title: ~5] OR "disabilities children" [Title: ~5] OR "handicapped children" [Title: ~5] OR "disabled adolescent" [Title: ~5] OR "disable adolescent" [Title: ~5] OR "disability adolescent" [Title: ~5] OR "disabilities adolescent" [Title: ~5] OR "handicapped adolescent" [Title: ~5] OR "disabled adolescents" [Title: ~5] OR "disable adolescents" [Title: ~5] OR "disability adolescents" [Title: ~5] OR "disabilities adolescents" [Title: ~5] OR "handicapped adolescents" [Title: ~5] OR "disabled women" [Title: ~5] OR "disable women" [Title: ~5] OR "disability women" [Title: ~5] OR "disabilities women" [Title: ~5] OR "handicapped women" [Title: ~5] OR "disabled mother" [Title: ~5] OR "disable mother" [Title: ~5] OR "disability mother" [Title: ~5] OR "disabilities mother" [Title: ~5] OR "handicapped mother" [Title: ~5] OR "disabled mothers" [Title: ~5] OR "disable mothers" [Title: ~5] OR "disability mothers" [Title: ~5] OR "disabilities mothers" [Title: ~5] OR "handicapped mothers" [Title: ~5] OR "disabled maternal" [Title: ~5] OR "disable maternal" [Title: ~5] OR "disability maternal" [Title: ~5] OR "disabilities maternal" [Title: ~5] OR "handicapped maternal" [Title: ~5] OR "disabled group" [Title: ~5] OR "disable group" [Title: ~5] OR "disability group" [Title: ~5] OR "disabilities group" [Title: ~5] OR "handicapped group" [Title: ~5] OR "disabled groups" [Title: ~5] OR "disable groups" [Title: ~5] OR "disability groups" [Title: ~5] OR "disabilities groups" [Title: ~5] OR "handicapped groups" [Title: ~5]))) AND ((*intervention*[Title/Abstract] OR *promoting*[Title/Abstract] OR *enhancing*[Title/Abstract] OR *policy*[Title/Abstract]))) AND (((("high country" [Title/Abstract: ~3] OR "high countries" [Title/Abstract:~3]) OR ("developed econom*"[Title/Abstract] OR "highly developed economy"[Title/Abstract] OR "high income econom*"[Title/Abstract])) OR (Aruba[Title/Abstract] OR Andorra[Title/Abstract] OR "United Arab Emirates"[Title/Abstract] OR "American Samoa"[Title/Abstract] OR "Antigua and Barbuda*"[Title/Abstract] OR Australia[Title/Abstract] OR Austria[Title/Abstract] OR Belgium[Title/Abstract] OR Bahrain[Title/Abstract] OR "The Bahamas*"[Title/Abstract] OR Bermuda[Title/Abstract] OR Barbados[Title/Abstract] OR "Brunei Darussalam"[Title/Abstract] OR Canada[Title/Abstract] OR Switzerland[Title/Abstract] OR "Channel Island"[Title/Abstract] OR Chile[Title/Abstract] OR Curaçao[Title/Abstract] OR "Cayman Islands"[Title/Abstract] OR Cyprus[Title/Abstract] OR Czechia*[Title/Abstract] OR Germany[Title/Abstract] OR Denmark[Title/Abstract] OR Spain[Title/Abstract] OR Estonia[Title/Abstract] OR Finland[Title/Abstract] OR France[Title/Abstract] OR "Faroe* Islands" or OR (“United Kingdom” [Title/Abstract] or “England” [Title/Abstract] or “Wales” [Title/Abstract] or “Scotland” [Title/Abstract] or “North Ireland”[Title/Abstract]) OR Gibraltar or Greece or Greenland or Guam or Guyana or ("Hong Kong"[Title/Abstract] OR "Hong Kong SAR"[Title/Abstract] OR "Hong Kong SAR, China"[Title/Abstract]) OR Croatia[Title/Abstract] OR Hungary[Title/Abstract] OR "Isle of Man"[Title/Abstract] OR Ireland[Title/Abstract] OR Iceland[Title/Abstract] OR Israel[Title/Abstract] OR Italy[Title/Abstract] OR Japan[Title/Abstract] OR "St. Kitts and Nevis" or ("Korea"[Title/Abstract] OR "Republic Korea"[Title/Abstract] OR "South Korea") or Kuwait or Lichtenstein or Lithuania or Luxembourg or Latvia or ("Macao"[Title/Abstract] OR "Macao SAR, China"[Title/Abstract]) OR "St. Martin"[Title/Abstract] OR

Monaco[Title/Abstract] OR Malta[Title/Abstract] OR "Northern Mariana Islands"[Title/Abstract] OR "New Caledonia"[Title/Abstract] OR Netherlands[Title/Abstract] OR Norway[Title/Abstract] OR Nauru[Title/Abstract] OR "New Zealand"[Title/Abstract] OR Oman[Title/Abstract] OR Panama[Title/Abstract] OR Poland[Title/Abstract] OR "Puerto Rico"[Title/Abstract] OR Portugal[Title/Abstract] OR "French Polynesia"[Title/Abstract] OR Qatar[Title/Abstract] OR Romania[Title/Abstract] OR "Saudi Arabia"[Title/Abstract] OR Singapore[Title/Abstract] OR "San Marino" or ("Slovakia"[Title/Abstract] OR "Slovak Republic") or Slovenia or Sweden or ("Sint Maarten"[Title/Abstract] OR "Sint Maarten"[Title/Abstract]) OR Seychelles[Title/Abstract] OR "Turks and Caicos Islands"[Title/Abstract] OR "Trinidad and Tobago" or ("Taiwan"[Title/Abstract] OR "Taiwan, China"[Title/Abstract]) OR Uruguay[Title/Abstract] OR "United States"[Title/Abstract] OR "British Virgin Islands" or ("Virgin Islands U.S."[Title/Abstract] OR "Virgin Islands"[Title/Abstract] OR "American Virgin Islands") or Venezuela NOT ("African-American*"[Title/Abstract] OR "African-American*"[Title/Abstract] OR "Mexican American*"[Title/Abstract] OR "American Indian*"[Title/Abstract] OR "Asian American*"[Title/Abstract] OR "native American*"[Title/Abstract]))) OR ("developed countr*" OR "developed nation?" OR "developed population?" OR "developed world" OR "developed state*" OR "highly developed countr*" OR "high developed countr*" OR "high income countr*" OR "high income nation?" OR "high income population?" OR "high income state*"))

Limits / filters:
None. No restrictions on language or publication date.

Last search: 20.03.2026

4) Ovid MEDLINE (Ovid)
   Initial search date: 16.04.2024
   Update search date: 20.03.2026

Search strategy:

1 ((developed or "high* developed" or "high income") adj (countr* or nation? or population? or world or state*)).tw.

2 ((Aruba or Andorra or "United Arab Emirates" or "American Samoa" or "Antigua and Barbuda*" or Australia or Austria or Belgium or Bahrain or "The Bahamas*" or Bermuda or Barbados or "Brunei Darussalam" or Canada or Switzerland or "Channel Island" or Chile or Curacao or "Cayman Islands" or Cyprus or Czechia* or Germany or Denmark or Spain or Estonia or Finland or France or "Faroe* Islands" or (“United Kingdom” or “England” or “Wales” or “Scotland” or “North Ireland”) or Gibraltar or Greece or Greenland or Guam or Guyana or ("Hong Kong" or "Hong Kong SAR" or "Hong Kong SAR, China") or Croatia or Hungary or "Isle of Man" or Ireland or Iceland or Israel or Italy or Japan or "St Kitts and Nevis" or ("Korea" or "Rep Korea" or "South Korea") or Kuwait or Lichtenstein or Lithuania or Luxembourg or Latvia or ("Macao" or "Macao SAR, China") or ("St Martin French* part" or "French St Martin") or Monaco or Malta or "Northern Mariana Islands" or "New Caledonia" or Netherlands or norway or Nauru or "New Zealand" or Oman or Panama or Poland or "Puerto Rico" or Portugal or "French Polynesia" or Qatar or Romania or "Saudi Arabia" or Singapore or "San Marino" or ("Slovakia" or "Slovak Republic") or Slovenia or Sweden or ("Sint Maarten" or "Sint Maarten") or Seychelles or "Turks and Caicos Islands" or "Trinidad and Tobago" or ("Taiwan" or "Taiwan, China") or Uruguay or "United States" or "British Virgin Islands" or ("Virgin Islands U S " or "Virgin Islands" or "American Virgin Islands") or (Venezuela or "Venezuela, RB")) not ("African-American*" or "African-American*" or "Mexican American*" or "American Indian*" or "Asian American*" or "native American*")).tw.

3 ((developed or "high* developed" or "high income") adj econom*).tw.
4 (high adj3 countr*).tw.
5 1 or 2 or 3 or 4
6 (disabili* or disable* or handicap* or deficien* or impair*).tw.
7 (physical* adj5 (impair* or deficien* or disable* or disabili* or handicap*)).ti.
8 (("cerebral pals*" or "spina bifida" or "muscular dystroph*" or arthriti* or "osteogenesis imperfecta" or "musculoskeletal abnormalit*" or "musculo-skeletal abnormalit*" or "muscular abnormalit*" or "skeletal abnormalit*" or "limb abnormalit*" or "brain injur*" or amput* or clubfoot or polio* or paraplegi* or paralys* or paralyz* or hemiplegi* or stroke* or "cerebrovascular accident*") adj2 (impair* or disabilit* or disabl* or handicap*)).ti.
9 (((visual* or vision or eye* or ocular) adj5 (loss* or impair* or deficien* or disable* or disabili* or handicap*)) or blind*).ti.
10 (autis* or dyslexi* or "Down* syndrome" or mongolism or "trisomy 21").ti.
11 (((hearing or acoustic or ear*) adj5 (loss* or impair* or deficien* or disable* or disabili* or handicap*)) or deaf*).ti.
12 ((intellectual* or educational* or mental* or psychological* or developmental) adj5 (impair* or retard* or deficien* or disable* or disabili* or handicap* or ill*)).ti.
13 ((mental* or emotional* or psychiatric or neurologic*) adj2 (disorder* or ill or illness*)).ti.
14 ((schizophreni* or psychos* or psychotic or schizoaffective or schizophreniform or dementia* or alzheimer*) adj2 (impair* or disabilit* or disabl* or handicap*)).ti.
15 ((depression or depressive or anxiety or psychiat* or well-being or "quality of life" or self-esteem or "self perception") adj2 (impair* or disabilit* or disabl* or handicap*) adj (severe or chronic or major)).ti.
16 ((mental* or emotional* or psychiatric or neurologic*) adj2 (disorder* or ill or illness*) adj (severe or chronic or major)).ti.
17 ((communication or language or speech or learning) adj5 disorder*).ti.
18 ((cognitive* or learning or mobility or sensory or visual* or vision or sight or hearing or physical* or mental* or intellectual*) adj2 (impair* or disabilit* or disabl* or handicap*)).ti
19 ((disable* or disabilit* or handicapped) adj5 (person* or people or child* or adolescen* or women or mother* or maternal or father* or men or parents or group*)).ti.
20 ((physical* or intellectual* or learning or psychiatric* or sensory or motor or neuromotor or cognitive or mental* or developmental or communication or learning) adj2 (disabilit* or disabl* or handicap*)).ti.
21 6 or 7 or 8 or 9 or 10 or 11 or 12 or 13 or 14 or 15 or 16 or 17 or 18 or 19 or 20
22 (intervention$ or promoting* or enhancing* or policy*).tw.
23 ("social inclusion" or "social participation" or "social interaction" or "social integration" or "community participation" or "community involvement" or "social network$" or "community inclusion" or “social capital”).tw.
24 5 and 21 and 22 and 23
Limits / filters:
None. No restrictions on language or publication date.
Last search: 20.03.2026

5) APA PsychInfo (Ovid)
Initial search date: 17.04.2024
Update search date: 20.03.2026

Search strategy:

1 ((developed or "high* developed" or "high income") adj (countr* or nation? or population? or world or state*)).tw.

2 ((Aruba or Andorra or "United Arab Emirates" or "American Samoa" or "Antigua and Barbuda*" or Australia or Austria or Belgium or Bahrain or "The Bahamas*" or Bermuda or Barbados or "Brunei Darussalam" or Canada or Switzerland or "Channel Island" or Chile or Curacao or "Cayman Islands" or Cyprus or Czechia* or Germany or Denmark or Spain or Estonia or Finland or France or "Faroe* Islands" or or (“United Kingdom” or “England” or “Wales” or “Scotland” or “North Ireland”) or Gibraltar or Greece or Greenland or Guam or Guyana or ("Hong Kong" or "Hong Kong SAR" or "Hong Kong SAR, China") or Croatia or Hungary or "Isle of Man" or Ireland or Iceland or Israel or Italy or Japan or "St Kitts and Nevis" or ("Korea" or "Rep Korea" or "South Korea") or Kuwait or Lichtenstein or Lithuania or Luxembourg or Latvia or ("Macao" or "Macao SAR, China") or ("St Martin French* part" or "French St Martin") or Monaco or Malta or "Northern Mariana Islands" or "New Caledonia" or Netherlands or norway or Nauru or "New Zealand" or Oman or Panama or Poland or "Puerto Rico" or Portugal or "French Polynesia" or Qatar or Romania or "Saudi Arabia" or Singapore or "San Marino" or ("Slovakia" or "Slovak Republic") or Slovenia or Sweden or ("Sint Maarten" or "Sint Maarten") or Seychelles or "Turks and Caicos Islands" or "Trinidad and Tobago" or ("Taiwan" or "Taiwan, China") or Uruguay or "United States" or "British Virgin Islands" or ("Virgin Islands U S " or "Virgin Islands" or "American Virgin Islands") or (Venezuela or "Venezuela, RB")) not ("African-American*" or "African-American*" or "Mexican American*" or "American Indian*" or "Asian American*" or "native American*")).tw.

3 ((developed or "high* developed" or "high income") adj econom*).tw.

4 (high adj3 countr*).tw

5 1 or 2 or 3 or 4

6 (disabili* or disable* or handicap* or deficien* or impair*).tw.

7 (physical* adj5 (impair* or deficien* or disable* or disabili* or handicap*)).ti.

8 (("cerebral pals*" or "spina bifida" or "muscular dystroph*" or arthriti* or "osteogenesis imperfecta" or "musculoskeletal abnormalit*" or "musculo-skeletal abnormalit*" or "muscular abnormalit*" or "skeletal abnormalit*" or "limb abnormalit*" or "brain injur*" or amput* or clubfoot or polio* or paraplegi* or paralys* or paralyz* or hemiplegi* or stroke* or "cerebrovascular accident*") adj2 (impair* or disabilit* or disabl* or handicap*)).ti.

9 (((visual* or vision or eye* or ocular) adj5 (loss* or impair* or deficien* or disable* or disabili* or handicap*)) or blind*).ti.

10 (autis* or dyslexi* or "Down* syndrome" or mongolism or "trisomy 21").ti.

11 (((hearing or acoustic or ear*) adj5 (loss* or impair* or deficien* or disable* or disabili* or handicap*)) or deaf*).ti.

12 ((intellectual* or educational* or mental* or psychological* or developmental) adj5 (impair* or retard* or deficien* or disable* or disabili* or handicap* or ill*)).ti.

13 ((mental* or emotional* or psychiatric or neurologic*) adj2 (disorder* or ill or illness*)).ti.

14 ((schizophreni* or psychos* or psychotic or schizoaffective or schizophreniform or dementia* or alzheimer*) adj2 (impair* or disabilit* or disabl* or handicap*)).ti.

15 ((depression or depressive or anxiety or psychiat* or well-being or "quality of life" or self-esteem or "self perception") adj2 (impair* or disabilit* or disabl* or handicap*) adj (severe or chronic or major)).ti.

16 ((mental* or emotional* or psychiatric or neurologic*) adj2 (disorder* or ill or illness*) adj (severe or chronic or major)).ti.

17 ((communication or language or speech or learning) adj5 disorder*).ti.

18 ((cognitive* or learning or mobility or sensory or visual* or vision or sight or hearing or physical* or mental* or intellectual*) adj2 (impair* or disabilit* or disabl* or handicap*)).ti.

19 ((disable* or disabilit* or handicapped) adj5 (person* or people or child* or adolescen* or women or mother* or maternal or father* or men or parents or group*)).ti.
20 ((physical* or intellectual* or learning or psychiatric* or sensory or motor or neuromotor or cognitive or mental* or developmental or communication or learning) adj2 (disabilit* or disabl* or handicap*)).ti.
21 6 or 7 or 8 or 9 or 10 or 11 or 12 or 13 or 14 or 15 or 16 or 17 or 18 or 19 or 20
22 (intervention$ or promoting* or enhancing* or policy*).tw.
23 ("social inclusion" or "social participation" or "social interaction" or "social integration" or "community participation" or "community involvement" or "social network$" or "community inclusion" or “social capital”).tw.
24 5 and 21 and 22 and 23
Limits / filters:
None. No restrictions on language or publication date.
Last search: 20.03.2026

6) ERIC (EBSCOhost)
Initial search date: 24.04.2024
Update search date: 20.03.2026

Search strategy:

S24	S5 AND S21 AND S22 AND S23	Expanders - Apply equivalent subjects<br>Search modes - Boolean/Phrase
S23	TI ((("social inclusion" OR "social participation" OR "social interaction" OR "social integration" OR "community participation" OR "community involvement" OR "social network$" OR "community inclusion"))) OR AB ((("social inclusion" OR "social participation" OR "social interaction" OR "social integration" OR "community participation" OR "community involvement" OR "social network$" OR "community inclusion" OR “social capital”)))	Expanders - Apply equivalent subjects<br>Search modes - Boolean/Phrase
S22	TI ((intervention$ or *promoting* or *enhancing* or *policy*)) OR AB ((intervention$ or *promoting* or *enhancing* or *policy*))	Expanders - Apply equivalent subjects<br>Search modes - Boolean/Phrase
S21	S6 OR S7 OR S8 OR S9 OR S10 OR S11 OR S12 OR S13 OR S14 OR S15 OR S16 OR S17 OR S18 OR S19 OR S20	Expanders - Apply equivalent subjects<br>Search modes - Boolean/Phrase
S20	TI ((physical* or intellectual* or learning or psychiatric* or sensory or motor or	Expanders - Apply equivalent subjects<br>Search modes - Boolean/Phrase

	neuromotor or cognitive or mental* or developmental or communication or learning) N2 (disabilit* or disabl* or handicap*))	
S19	TI ((disable* or disabilit* or handicapped) N5 (person* or people or child* or adolescen* or women or mother* or maternal or father* or men or parents or group*))	Expanders - Apply equivalent subjects<br>Search modes - Boolean/Phrase
S18	TI ((cognitive* or learning or mobility or sensory or visual* or vision or sight or hearing or physical* or mental* or intellectual*) N2 (impair* or disabilit* or disabl* or handicap*))	Expanders - Apply equivalent subjects<br>Search modes - Boolean/Phrase
S17	TI =((communication or language or speech or learning) N5 disorder*)	Expanders - Apply equivalent subjects<br>Search modes - Boolean/Phrase
S16	TI ((mental* or emotional* or psychiatric or neurologic*) N2 (disorder* or ill or illness*) N (severe or chronic or major))	Expanders - Apply equivalent subjects<br>Search modes - Boolean/Phrase
S15	TI ((depression or depressive or anxiety or psychiat* or well-being or “quality of life” or self-esteem or “self perception”) N2 (impair* or disabilit* or disabl* or handicap*) N (severe or chronic or major))	Expanders - Apply equivalent subjects<br>Search modes - Boolean/Phrase
S14	TI ((schizophreni* or psychos* or psychotic or schizoaffective or schizophreniform or dementia* or alzheimer*) N2 (impair* or disabilit* or disabl* or handicap*))	Expanders - Apply equivalent subjects<br>Search modes - Boolean/Phrase
S13	TI ((mental* or emotional* or psychiatric or neurologic*) N2 (disorder* or ill or illness*))	Expanders - Apply equivalent subjects<br>Search modes - Boolean/Phrase
S12	TI ((intellectual* or educational* or mental* or psychological* or developmental) N5 (impair* or retard* or deficien* or disable* or disabili* or handicap* or ill*))	Expanders - Apply equivalent subjects<br>Search modes - Boolean/Phrase
S11	TI (((hearing or acoustic or ear*) N (loss* or impair* or deficien* or disable* or disabili* or handicap*)) or deaf*)	Expanders - Apply equivalent subjects<br>Search modes - Boolean/Phrase

S10	TI (autis* or dyslexi* or “Down* syndrome” or mongolism or “trisomy 21”)	Expanders - Apply equivalent subjects<br>Search modes - Boolean/Phrase
S9	TI (((visual* or vision or eye* or ocular) N (loss* or impair* or deficien* or disable* or disabili* or handicap*)) or blind*)	Expanders - Apply equivalent subjects<br>Search modes - Boolean/Phrase
S8	TI ((“cerebral pals*“ or “spina bifida” or “muscular dystroph*“ or arthriti* or “osteogenesis imperfecta” or “musculoskeletal abnormalit*“ or “musculo-skeletal abnormalit*“ or “muscular abnormalit*“ or “skeletal abnormalit*“ or “limb abnormalit*“ or “brain injur*“ or amput* or clubfoot or polio* or paraplegi* or paralys* or paralyz* or hemiplegi* or stroke* or “cerebrovascular accident*“) N2 (impair* or disabilit* or disabl* or handicap*))	Expanders - Apply equivalent subjects<br>Search modes - Boolean/Phrase
S7	TI (physical* N (impair* or deficien* or disable* or disabili* or handicap*))	Expanders - Apply equivalent subjects<br>Search modes - Boolean/Phrase
S6	TI ((disabili* OR disable* OR handicap* OR deficien* OR impair*)) OR AB ((disabili* OR disable* OR handicap* OR deficien* OR impair*))	Expanders - Apply equivalent subjects<br>Search modes - Boolean/Phrase
S5	S1 OR S2 OR S3 OR S4	Expanders - Apply equivalent subjects<br>Search modes - Boolean/Phrase
S4	TI (high N3 countr*) OR AB (high N3 countr*)	Expanders - Apply equivalent subjects<br>Search modes - Boolean/Phrase
S3	TI (((developed or “high* developed” or "high income”) N (econom*))) OR AB (((developed or “high* developed” or "high income”) N (econom*)))	Expanders - Apply equivalent subjects<br>Search modes - Boolean/Phrase
S2	TI (((Aruba or Andorra or "United Arab Emirates" or "American Samoa" or "Antigua and Barbuda*" or Australia or Austria or Belgium or Bahrain or "The Bahamas*" or Bermuda or Barbados or "Brunei Darussalam" or Canada or Switzerland or "Channel Island" or Chile or Curacao or "Cayman Islands" or Cyprus or Czechia* or Germany or Denmark or Spain or Estonia or Finland	Expanders - Apply equivalent subjects<br>Search modes - Boolean/Phrase

	or France or "Faroe* Islands" or Gibraltar or Greece or Greenland or Guam or Guyana or ("Hong Kong" or "Hong Kong SAR" or "Hong Kong SAR, China") or Croatia or Hungary or "Isle of Man" or Ireland or Iceland or Israel or Italy or Japan or "St. Kitts and Nevis" or ("Korea" or "Rep. Korea" or "South Korea") or Kuwait or Lichtenstein or Lithuania or Luxembourg or Latvia or ("Macao" or "Macao SAR, China") or ("St. Martin French* part" or "French St Martin") or Monaco or Malta or "Northern Mariana Islands" or "New Caledonia" or Netherlands or norway or Nauru or "New Zealand" or Oman or Panama or Poland or "Puerto Rico" or Portugal or "French Polynesia" or Qatar or Romania or "Saudi Arabia" or Singapore or "San Marino" or ("Slovakia" or "Slovak Republic") or Slovenia or Sweden or ("Sint Maarten" or "Sint Maarten") or Seychelles or "Turks and Caicos Islands" or "Trinidad and Tobago" or ("Taiwan" or "Taiwan, China") or Uruguay or "United States" or "British Virgin Islands" or ("Virgin Islands U.S." or "Virgin Islands" or "American Virgin Islands") or (Venezuela or "Venezuela, RB")) NOT (“African-American*“ or “African-American*“ or “Mexican American*“ or “American Indian*“ or “Asian American*“ or “native American*“))) OR AB (((Aruba or Andorra or "United Arab Emirates" or "American Samoa" or "Antigua and Barbuda*" or Australia or Austria or Belgium or Bahrain or "The Bahamas*" or Bermuda or Barbados or "Brunei Darussalam" or Canada or Switzerland or "Channel Island" or Chile or Curacao or "Cayman Islands" or Cyprus or Czechia* or Germany or Denmark or Spain or Estonia or Finland or France or "Faroe* Islands" or Gibraltar or Greece or Greenland or Guam or Guyana or ("Hong Kong" or "Hong Kong SAR" or "Hong Kong SAR, China") or	

	Croatia or Hungary or "Isle of Man" or Ireland or Iceland or Israel or Italy or Japan or "St. Kitts and Nevis" or ("Korea" or "Rep. Korea" or "South Korea") or Kuwait or Lichtenstein or Lithuania or Luxembourg or Latvia or ("Macao" or "Macao SAR, China") or ("St. Martin French* part" or "French St Martin") or Monaco or Malta or "Northern Mariana Islands" or "New Caledonia" or Netherlands or norway or Nauru or "New Zealand" or Oman or Panama or Poland or "Puerto Rico" or Portugal or "French Polynesia" or Qatar or Romania or "Saudi Arabia" or Singapore or "San Marino" or ("Slovakia" or "Slovak Republic") or Slovenia or Sweden or ("Sint Maarten" or "Sint Maarten”) or Seychelles or "Turks and Caicos Islands" or "Trinidad and Tobago" or ("Taiwan" or "Taiwan, China") or Uruguay or "United States" or "British Virgin Islands" or ("Virgin Islands U.S." or "Virgin Islands" or "American Virgin Islands") or (Venezuela or "Venezuela, RB")) NOT (“African-American*“ or “African-American*“ or “Mexican American*“ or “American Indian*“ or “Asian American*“ or “native American*“)))	
S1	TI (((developed or “high* developed” or “high income”) N (countr* or nation? or population? or world or state*))) OR AB (((developed or “high* developed” or “high income”) N (countr* or nation? or population? or world or state*)))	Expanders - Apply equivalent subjects<br>Search modes - Boolean/Phrase

Limits / filters:

None. No restrictions on language or publication date.

Last search: 20.03.2026

7) CINAHL Complete (EBSCOhost)
   Initial search date: 24.04.2024
   Update search date: 20.03.2026

Search strategy:

S24	S5 AND S21 AND S22 AND S23	Expanders - Apply equivalent subjects<br>Search modes - Boolean/Phrase
S23	TI ((("social inclusion" OR "social participation" OR "social interaction" OR "social integration" OR "community participation" OR "community involvement" OR "social network$" OR "community inclusion"))) OR AB ((("social inclusion" OR "social participation" OR "social interaction" OR "social integration" OR "community participation" OR "community involvement" OR "social network$" OR "community inclusion" OR “social capital”)))	Expanders - Apply equivalent subjects<br>Search modes - Boolean/Phrase
S22	TI ((intervention$ or *promoting* or *enhancing* or *policy*)) OR AB ((intervention$ or *promoting* or *enhancing* or *policy*))	Expanders - Apply equivalent subjects<br>Search modes - Boolean/Phrase
S21	(S6 OR S7 OR S8 OR S9 OR S10 OR S11 OR S12 OR S13 OR S14 OR S15 OR S16 OR S17 OR S18 OR S19 OR S20)	Expanders - Apply equivalent subjects<br>Search modes - Boolean/Phrase
S20	TI ((physical* or intellectual* or learning or psychiatric* or sensory or motor or neuromotor or cognitive or mental* or developmental or communication or learning) N2 (disabilit* or disabl* or handicap*))	Expanders - Apply equivalent subjects<br>Search modes - Boolean/Phrase
S19	TI ((disable* or disabilit* or handicapped) N5 (person* or people or child* or adolescen* or women or mother* or maternal or father* or men or parents or group*))	Expanders - Apply equivalent subjects<br>Search modes - Boolean/Phrase
S18	TI ((cognitive* or learning or mobility or sensory or visual* or vision or sight or hearing or physical* or mental* or intellectual*) N2 (impair* or disabilit* or disabl* or handicap*))	Expanders - Apply equivalent subjects<br>Search modes - Boolean/Phrase
S17	TI ((communication or language or speech or learning) N5 disorder*)	Expanders - Apply equivalent subjects<br>Search modes - Boolean/Phrase

S16	TI ((mental* or emotional* or psychiatric or neurologic*) N2 (disorder* or ill or illness*) N (severe or chronic or major))	Expanders - Apply equivalent subjects<br>Search modes - Boolean/Phrase
S15	TI ((depression or depressive or anxiety or psychiat* or well-being or “quality of life” or self-esteem or “self perception”) N2 (impair* or disabilit* or disabl* or handicap*) N (severe or chronic or major))	Expanders - Apply equivalent subjects<br>Search modes - Boolean/Phrase
S14	TI ((schizophreni* or psychos* or psychotic or schizoaffective or schizophreniform or dementia* or alzheimer*) N2 (impair* or disabilit* or disabl* or handicap*))	Expanders - Apply equivalent subjects<br>Search modes - Boolean/Phrase
S13	TI ((mental* or emotional* or psychiatric or neurologic*) N2 (disorder* or ill or illness*))	Expanders - Apply equivalent subjects<br>Search modes - Boolean/Phrase
S12	TI ((intellectual* or educational* or mental* or psychological* or developmental) N5 (impair* or retard* or deficien* or disable* or disabili* or handicap* or ill*))	Expanders - Apply equivalent subjects<br>Search modes - Boolean/Phrase
S11	TI (((hearing or acoustic or ear*) N (loss* or impair* or deficien* or disable* or disabili* or handicap*)) or deaf*)	Expanders - Apply equivalent subjects<br>Search modes - Boolean/Phrase
S10	TI (autis* or dyslexi* or “Down* syndrome” or mongolism or “trisomy 21”)	Expanders - Apply equivalent subjects<br>Search modes - Boolean/Phrase
S9	TI (((visual* or vision or eye* or ocular) N (loss* or impair* or deficien* or disable* or disabili* or handicap*)) or blind*)	Expanders - Apply equivalent subjects<br>Search modes - Boolean/Phrase
S8	TI ((“cerebral pals*“ or “spina bifida” or “muscular dystroph*“ or arthriti* or “osteogenesis imperfecta” or “musculoskeletal abnormalit*“ or “musculo-skeletal abnormalit*“ or “muscular abnormalit*“ or “skeletal abnormalit*“ or “limb abnormalit*“ or “brain injur*“ or amput* or clubfoot or polio* or paraplegi* or paralys* or paralyz* or hemiplegi* or stroke* or	Expanders - Apply equivalent subjects<br>Search modes - Boolean/Phrase

	“cerebrovascular accident*“) N2 (impair* or disabilit* or disabl* or handicap*))	
S7	TI (physical* N (impair* or deficien* or disable* or disabili* or handicap*))	Expanders - Apply equivalent subjects<br>Search modes - Boolean/Phrase
S6	TI ((disabili* OR disable* OR handicap* OR deficien* OR impair*)) OR AB ((disabili* OR disable* OR handicap* OR deficien* OR impair*))	Expanders - Apply equivalent subjects<br>Search modes - Boolean/Phrase
S5	S1 OR S2 OR S3 OR S4	Expanders - Apply equivalent subjects<br>Search modes - Boolean/Phrase
S4	TI (high N3 countr*) OR AB (high N3 countr*)	Expanders - Apply equivalent subjects<br>Search modes - Boolean/Phrase
S3	TI (((developed or “high* developed” or "high income”) N (econom*))) OR AB (((developed or “high* developed” or "high income”) N (econom*)))	Expanders - Apply equivalent subjects<br>Search modes - Boolean/Phrase
S2	TI (((Aruba or Andorra or "United Arab Emirates" or "American Samoa" or "Antigua and Barbuda*" or Australia or Austria or Belgium or Bahrain or "The Bahamas*" or Bermuda or Barbados or "Brunei Darussalam" or Canada or Switzerland or "Channel Island" or Chile or Curacao or "Cayman Islands" or Cyprus or Czechia* or Germany or Denmark or Spain or Estonia or Finland or France or "Faroe* Islands" or Gibraltar or Greece or Greenland or Guam or Guyana or ("Hong Kong" or "Hong Kong SAR" or "Hong Kong SAR, China") or Croatia or Hungary or "Isle of Man" or Ireland or Iceland or Israel or Italy or Japan or "St. Kitts and Nevis" or ("Korea" or "Rep. Korea" or "South Korea") or Kuwait or Lichtenstein or Lithuania or Luxembourg or Latvia or ("Macao" or "Macao SAR, China") or ("St. Martin French* part" or "French St Martin") or Monaco or Malta or "Northern Mariana Islands" or "New Caledonia" or Netherlands or norway or Nauru or "New Zealand" or Oman or Panama or Poland or "Puerto Rico" or Portugal or "French Polynesia" or Qatar or Romania or "Saudi	Expanders - Apply equivalent subjects<br>Search modes - Boolean/Phrase

	Arabia" or Singapore or "San Marino" or ("Slovakia" or "Slovak Republic") or Slovenia or Sweden or ("Sint Maarten" or "Sint Maarten") or Seychelles or "Turks and Caicos Islands" or "Trinidad and Tobago" or ("Taiwan" or "Taiwan, China") or Uruguay or "United States" or "British Virgin Islands" or ("Virgin Islands U.S." or "Virgin Islands" or "American Virgin Islands") or (Venezuela or "Venezuela, RB")) NOT ("African-American*" or "African-American*" or "Mexican American*" or "American Indian*" or "Asian American*" or "native American*"))) AND AB (((Aruba or Andorra or "United Arab Emirates" or "American Samoa" or "Antigua and Barbuda*" or Australia or Austria or Belgium or Bahrain or "The Bahamas*" or Bermuda or Barbados or "Brunei Darussalam" or Canada or Switzerland or "Channel Island" or Chile or Curacao or "Cayman Islands" or Cyprus or Czechia* or Germany or Denmark or Spain or Estonia or Finland or France or "Faroe* Islands" or Gibraltar or Greece or Greenland or Guam or Guyana or ("Hong Kong" or "Hong Kong SAR" or "Hong Kong SAR, China") or Croatia or Hungary or "Isle of Man" or Ireland or Iceland or Israel or Italy or Japan or "St. Kitts and Nevis" or ("Korea" or "Rep. Korea" or "South Korea") or Kuwait or Lichtenstein or Lithuania or Luxembourg or Latvia or ("Macao" or "Macao SAR, China") or ("St. Martin French* part" or "French St Martin") or Monaco or Malta or "Northern Mariana Islands" or "New Caledonia" or Netherlands or norway or Nauru or "New Zealand" or Oman or Panama or Poland or "Puerto Rico" or Portugal or "French Polynesia" or Qatar or Romania or "Saudi Arabia" or Singapore or "San Marino" or ("Slovakia" or "Slovak Republic") or Slovenia or Sweden or ("Sint Maarten" or "Sint Maarten") or Seychelles or "Turks	

	and Caicos Islands" or "Trinidad and Tobago" or ("Taiwan" or "Taiwan, China") or Uruguay or "United States" or "British Virgin Islands" or ("Virgin Islands U.S." or "Virgin Islands" or "American Virgin Islands") or (Venezuela or "Venezuela, RB")) NOT (“African-American*“ or “African-American*“ or “Mexican American*“ or “American Indian*“ or “Asian American*“ or “native American*“)))	
S1	TI (((developed or “high* developed” or “high income”) N (countr* or nation? or population? or world or state*))) OR AB (((developed or “high* developed” or “high income”) N (countr* or nation? or population? or world or state*)))	Expanders - Apply equivalent subjects<br>Search modes - Boolean/Phrase

Limits / filters:
None. No restrictions on language or publication date.

Last search: 20.03.2026

Appendix 5

Records retrieved from each database and deduplication process

Database	Platform	Records retrieved
**Web of Science**	Clarivate	1306
**PubMed**	NCBI	587
**MEDLINE**	Ovid	576
**APA PsycInfo**	Ovid	567
**Scopus**	Elsevier	428
**CINAHL Complete**	EBSCOhost	426
**ERIC**	EBSCOhost	104

**Total**	**3994**

Appendix 6

Extraction table

General information	Title	
	Authors	
	Year of publication	
	Reference/Citation	
	Country/region	
Characteristics of included studies	**Methods**	
	Study aim/objective	
	Study design	Randomized controlled trial (RCT)
		Non-randomized controlled trial (non-RCT)
		Single-group controlled before-and-after
		Interrupted time-series designs
		Quasi-experimental methods (e.g. regression discontinuity design (RDD), difference-in-differences, matched designs)
		Others (specify)

	Method of recruitment		
	Control condition		
	Time points and duration of follow-up	Baseline	
		T1	
		T2	
		T3	
		T4	
		Descriptive analysis	
	Social inclusion measure		
	Statistical analysis	t-test	
		Chi-square test	
		ANOVA	
		ANCOVA	
		Regression	
Participants	Children or adults?	Children with disabilities (0-18y)	
		Adults with disabilities (18-65y)	
		People with disabilities (0-65y)	
	Disability situation		
	Inclusion criteria		
	Exclusion criteria		
	Sample size	Intervention group	
		Control group	
		Drop outs	
	Population characteristics	Age	
		Gender	
		Disability situation	
		Socio-economic status	
		Cultural/ national background	
		Work situation	
		Living situation	
		Educational background	
		Other	
Intervention characteristics	Intervention:		
	Stated main aim/purpose of the intervention		
	Frequency:		
	Duration of intervention:		
	Length of each session		
	Setting (location of the intervention)		
	Intervention was delivered by	Intervention therapist	
		coach	
		Therapist	
		occupational	
		community members	

		etc.	
	Social inclusion intervention categories (social inclusion matrix)		
Risk of bias	RoB for RCT		
	ROBINS-I for non-RCT		
Social inclusion outcomes	Social inclusion outcomes categories (social inclusion matrix)		
	Who answered the questionnaire	Children with disabilities (0-18y)	
		Adults with disabilities (18-65y)	
		Children & adults with disabilities (0-65y)	
		Parents	
		Teachers	
		Support staff (educator,etc.)	
		Medical staff (kinesitherapy, nurses, rehabilitation staff, etc.)	
		Other	
	Results:		Overall positive
			Mixed results
			No effect
			Negative effect
		Effect size	
		Empirical results	
	Subgroups results:	Subgroup results	
			Overall positive
			Mixed results
			No effect
		Effect size	
		Empirical results	
		Subgroups results	
			Overall positive
			Mixed results
			No effect
		Effect size	
		Empirical results	
		Subgroups results:	
		…	…
	Secondary results	Secondary results 1	
			Overall positive
			Mixed results
			No effect
		Effect size	
		Empirical results	
		Secondary results 1	
			Overall positive

			Mixed results
			No effect
		Effect size	
		Empirical results	
		Secondary results 3	
		…	…
Comments			

Appendix 7

Table 3. Overview of included studies, intervention characteristics, and reported effectiveness of social inclusion interventions

Studies with statistically significant positive effects (n=20)

STUDY	POPULATION (AGE, DISABILITY)	INTERVENTION CATEGORY (CBR; ECOLOGICAL LEVEL)	INTERVENTION (BRIEF DESCRIPTION)	OUTCOME (CBR DOMAIN; ECOLOGICAL LEVEL)	SPECIFIC OUTCOME	KEY STATISTICS
**ISON ET AL. 2010**	Children, no disability	Relationship, marriage & family; Interpersonal; & organizational	Disability awareness program	Peer and community relationship; Interpersonal; & Organizational	Attitudes and acceptance toward disability	p<0.001
**WILLIS ET AL. 2018**	Children, cerebral palsy and other disabilities	Assistive technology and rehabilitation ; Individual ; & Interpersonal	Goal-directed family-centred intervention	Community participation; Individual	Achieving individualized participation goals	p<0.01 ES = 0.74
**AFSHARNEJAD ET AL. 2022**	Children, autism spectrum disorder	Relationship, marriage & family; Individual	KONTAKT® social skills group training	Social and communication skills; Individual	Personally personal goals<br>Social behaviour	p=0.04 ES=0.35;<br>p=0.03 ES=0.20
**WANG ET AL. 2015**	Adults, chronic stroke	Assistive technology & rehabilitation; Individual; & Interpersonal	Caregiver-mediated home-based rehabilitation	Community participation; Individual	Social participation and activities of daily living	p=0.019;<br>p=0.022
**MODUGNO ET AL. 2010**	Adults, Parkinson's disease	Culture & arts; & Recreation, leisure and sports ; Individual	Theatre workshop rehabilitation program	Interpersonal and Family relationship; Individual	Social support	p<0.001
**CALIENDO ET AL. 2021**	Children, autism spectrum disorder	Relationship, marriage & family; Individual	Neuro-psychomotor play-based therapy	Social and communication skills; & Social behaviour; Individual	Behaviours in social relations	p<0.0001

**TATENO ET AL. 2021**	Children, autism spectrum disorder	Relationship, marriage & family; & Assistive technology and rehabilitation ; Individual	Early Start Denver Model intervention	Social and communication skills; & Social behaviour; & Interpersonal and Family relationship; Individual	Interpersonal relationships and socialization	p=0.0094
**TAYLOR ET AL. 2016**	Adults, chronic musculoskeletal pain	Assistive technology and rehabilitation; Individual	Community-based nonpharmacological group intervention (COPERS)	Interpersonal and Family relationship; Interpersonal	Social integration and support	95%CI: 0.8 [0.4 to 1.2]
**CHEUNG ET AL. 2021**	Children, autism spectrum disorder	Assistive technology and rehabilitation; Individual	School-based social cognitive intervention	Social and communication skills; Individual	Social skills	p=0.005 ES=1.03
					Client-centred social goals	ES=0.052 p=0.047
**RODRÍGUEZ-RIVAS ET AL. 2021**	Children & adults, mental disorders	Relationship, marriage & family; Individual	Virtual stigma reduction program	Peer and community relationship; Interpersonal	Social distancing and stigma	p=0.004 ES=1.11; p=0.000 ES=2.33
**H.-H. HUANG ET AL. 2018**	Children, motor disabilities	Relationship, marriage and family; & Assistive technology & rehabilitation; Individual	Ride-on car training with social interaction program	Social and communication skills; Individual	Social functioning	p=0.03 ES=0.123
**MYLES ET AL. 2000**	Adults, learning disabilities	Moving from institutional life to community living; Individual<br>Policies and programs ; Socio-policial	Resettlement to community accommodation	Community participation; Individual	Community involvement	p<0.005
**JAMES ET AL. 2021**	Adults, learning disabilities	Civic-education; individual	Promote the Vote; electoral participation program	Community participation; Individual	Election participation, Registered to vote, Aware of their rights	p<0.001<br>p=0.0012;<br>p=0.0035;

**CHONG ET AL. 2024**	Children & adults, intellectual developmental disabilities	Relationship, marriage & family; Individual	Self-advocacy training program (OLOV)	Social and communication skills; Individual	Communication skills	p<0.01 ES=0.813
**MARTÍNEZ-TUR ET AL., 2025**	Adults, intellectual and developmental disabilities	Personal-Assistance; & Supported internship and works skills; Individual	Customized Employment	Social behaviour; & Community integration; & Community participation; & Interpersonal and Family relationship; Individual	Visibility<br>sense of belonging,<br>new places visited	p<0.001 ES=0.02;<br>p<0.001 ES=0.13;<br>p>0.001 ES = 0.19
**HSIAO ET AL., 2024**	Children, autism spectrum disorder	Relationship, marriage and family; Individual; & Interpersonal	PEERS® social skills training	Social and communication skills; & Social behaviour; & Violence and abuse; Individual	social skills knowledge<br><br>School bullying	p<0.001 ES=0.83<br><br>p<0.001 ES=0.59;
**CHAN ET AL., 2023**	Children, autism spectrum disorder	Relationship, marriage and family; Individual	The Transporters App animated video intervention	Social and communication skills; Individual	Emotion recognition	p<0.001 ES=0.445
**IMAMOTO ET AL., 2025**	Adults, psychiatric conditions	Relationship, marriage and family; & Assistive technology and rehabilitation ; Individual	OTIPM-based occupational therapy	Social and communication skills; & Social behaviour; & Community participation; Individual	Occupational performance<br><br>social interaction	p<0.001 ES=0.79<br><br>p<0.001 ES=0.72
**ALECU & ONEA, 2025**	Children, autism spectrum disorder	Relationship, marriage and family; Individual	Therapeutic swimming programme (ABA strategies, sensory integration, and positive reinforcement)	Social and communication skills; & Social behaviour; Individual	in self-confidence and social behaviors	p=0.001 ES=0.81
**REDDY ET AL., 2025**	Adults, subacute stroke	Assistive technology and rehabilitation; Individual	Transcranial direct current stimulation (tDCS) combined with gait-oriented motor training	Social inclusion; Individual<br><br>Community participation; individual	Social participation	p=0.001 ES=0.67

<u>Studies with no statistically significant effects (n=14)</u>

STUDY	POPULATION (AGE, DISABILITY)	INTERVENTION CATEGORY (CBR; ECOLOGICAL LEVEL)	INTERVENTION (BRIEF DESCRIPTION)	OUTCOME (CBR DOMAIN; ECOLOGICAL LEVEL)	SPECIFIC OUTCOME	KEY STATISTICS
**VAN VEEN ET AL. 2021**	Adults, severe non-psychotic mental disorders	Assistive technology & rehabilitation; Individual	Interpersonal community psychiatric treatment	Community participation; Individual	Social network quality and quantity	p=0.773;<br>p=0.072

**H. HUANG ET AL. 2018**	Children, motor disabilities	Assistive technology & rehabilitation; Individual	Modified ride-on cars and family-centred intervention	Social and communication skills; Individual	Social persistence with adults and children	p=0.88; p=0.86
**SHERIDAN ET AL. 2015**	Adults, enduring mental illness	Personal assistance; Individual & Interpersonal<br>Relationship, marriage and family; & Recreation, Leisure and sports Interpersonal	Supported socialisation with community volunteers	Social and communication skills; & Social behaviour; Interpersonal<br>Community participation; Community	Social family, or romantic loneliness<br>Social functioning	p=0.27; p=0.90<br>p > 0.05
**TARRANT ET AL. 2021**	Adults, aphasia post-stroke	Recreation, leisure & sports; Individual	Group singing intervention (SPA)	Community participation; Individual	Social integration	p=0.22
**BORGEN ET AL. 2023**	Adults, traumatic brain injury	Assistive technology & rehabilitation; Individual	Tailored home-based goal-oriented rehabilitation	Community participation; Individual	Social participation	p=0.29
**OKAJIMA ET AL. 2021**	Children, autism spectrum disorder	Relationship, marriage & family; Individual; & Interpersonal	Social skills and parenting training	Social and communication skills; Individual	Social behaviour, communication, motivation, awareness	No significant according to study authors (n.s.)
**KENDALL ET AL. 2007**	Adults, stroke	Assistive technology & rehabilitation; Individual	Chronic disease self-management group course	Social behaviour; Individual	Social roles and family roles	No significant effects reported (n.s.)
**SLAMAN ET AL. 2015**	Adults, cerebral palsy	Recreation, leisure & sports; Individual	Active lifestyle and sports participation intervention	Community participation; Individual	Social participation and social roles<br>Social support	95%CI: −0.22 [–0.50, 0.07]<br>95%CI: 0.04 [–0.60, 0.53]<br>95%CI: −0.08 [–1.99, 1.83]
**ADAMUS ET AL. 2022**	Adults, severe mental illness	Personal assistance; & Moving from institutional life to community living; Individual	Independent supported housing	Social behaviour; & Community participation; Individual	Social inclusion and participation	95%CI: 6.28 [–0.08 to 13.35]
**SANCHES ET AL. 2020**	Adults, severe mental illness	Relationship, marriage and family; &Assistive technology & rehabilitation; Individual	Boston University Psychiatric Rehabilitation (BPR)	Community participation; Individual	Social functioning, participation, Hours of participation	p=0.234; p=0.893; p=0.456

**LORANT ET AL. 2019**	Adults, severe mental illness	Policies & programs; Socio-political	Mental health care reform — service networks	Community participation; & Interpersonal and Family relationship; Individual	Social integration	p=0.20
**HASSIOTIS ET AL. 2018**	Adults, intellectual disabilities	Relationship, marriage & family; Organisational	Positive Behaviour Support training for staff	Community participation; Individual	Community participation	95%CI: −0.57–1.74
**CHIEN ET AL., 2024**	Children, developmental disabilities	Relationship, marriage and family; Interpersonal	Occupational Performance Coaching	Social and communication skills; Individual	Community participation satisfaction, frequency, involvement	p = 0.355<br>p = 0.611<br>p = 0.954
**STANLEY ET AL., 2025**	Adults, mild to moderate mental illness	Recreation, leisure and sports ; Individual	Nature Scripts nature prescribing programme	Community participation; Individual	Contact with people in community	p= 0.058

<u>Studies with mixed effects (n (n=16)</u>

**STUDY**	**POPULATION (AGE, DISABILITY)**	**INTERVENTION CATEGORY (CBR; ECOLOGICAL LEVEL)**	**INTERVENTION (BRIEF DESCRIPTION)**	**OUTCOME (CBR DOMAIN; ECOLOGICAL LEVEL)**	**SPECIFIC OUTCOME**	**KEY STATISTICS**
**GREEN ET AL. 2022**	Children, autism spectrum disorder	Relationship, marriage & family; Interpersonal	Caregiver-mediated video feedback intervention (PACT-G)	Social and communication skills; Individual	Social communication<br>Child communication	p=0.85 (no significant effect)<br>p = 0.0002 (positive effect)
**Y.-C. CHOU ET AL. 2011**	Adults, intellectual disabilities	Personal assistance; Interpersonal	Active Support Training for staff	Community participation; Individual<br>Interpersonal and Family relationship; Individual	Community inclusion<br>Social network with friends and family	p<0.001 (positive effect)<br>p=0.01 (negative effect)<br>p=0.00 (negative effect)
**YOO ET AL. 2014**	Children, autism spectrum disorder	Relationship, marriage & family; Individual; & Interpersonal	Parent-assisted social skills training (PEERS®)	Social and communication skills; Individual<br>Social behaviour; & Community integration; Individual	Social communication and interaction<br>Social competence (SSRS)	p<0.01 (positive effect)<br>p=0.03 (negative effect)

**KIEGALDIE ET AL. 2023**	Adults, various disabilities	Supported internship and works skills; Individual	Supported internship model (IPP)	Community participation; Individual	Work skills and readiness<br><br>Work motivation	p=0.046 (no significant effect)<br><br>p=0.048 (positive effect)
**HOLEVA ET AL. 2024**	Children, autism spectrum disorder	Relationship, marriage and family; Individual	Robot-assisted psychological intervention	Social and communication skills; Individual<br><br>Social behaviour; & Community integration; & Interpersonal and Family relationship; Individual	Speech-based and gesture-based social interaction<br><br>Peer relationships and prosocial behaviour	p=0.003; p=0.039 (positive effect)<br><br>p = 0.466<br>p=0.098<br>(no significant effect)
**TIMPSON ET AL. 2019**	Adults, traumatic brain injury	Assistive technology & rehabilitation; Individual	Advanced therapy for traumatic brain injury (AdvTx)	Social inclusion; & Community participation; Individual<br><br>Interpersonal and Family relationship; Individual	Community participation<br><br>Going out and social relations	p<0.001 (positive effect)<br><br>p=0.072;<br>p=0.227 (no significant effect)
**CHOU ET AL. 2019**	Children & adults, Rett syndrome	Assistive technology & rehabilitation; Individual	Group-based neurological music therapy	Social and communication skills; & Social behaviour; & Interpersonal and Family relationship; Individual<br><br>Social and communication skills; Individual	Socialization and interpersonal relationships<br><br>Communication skills<br><br>Daily living skills	p<0.01;<br>p<0.005 (positive effect)<br><br>p<0.05 (positive effect)<br><br>no significant effect according to study authors
**BOGNER ET AL. 2019**	Adults, traumatic brain injury	Relationship, marriage & family; & Assistive technology and rehabilitation ; Interpersonal	Family attendance during rehabilitation therapy	Social inclusion; Community participation; Individual	Community participation<br><br>Going out into the community<br><br>Social relations	p=0.08 (no significant effect)<br><br>p=0.02 (positive effect)<br><br>p=0.29 (no significant effect)
**BAUMGARDT ET AL. 2020**	Adults, chronic mental illness	Assistive technology and rehabilitation ; Individual	Community-based clinical social work	Social and communication skills; & Community participation; Individual	Social inclusion	p=0.03 ES=0.158 (positive effect)

				Interpersonal and Family relationship; Individual	Social relationships	p=0.381 (no significant effect)
**HEPPE ET AL. 2020**	Children & adults, visual impairment	Relationship, marriage & family; Individual & Interpersonal	Community-based mentoring program	Social inclusion; & Interpersonal and Family relationship; Individual	Social participation and network size	95%CI: −0.30, 0.21<br>95%CI: −1.42, 6.39; (no significant effect
				Community participation; Individual; & Interpersonal	Satisfaction with social support	95%CI: 0.02–0.49 d=0.38 (positive effect)
**CHOU ET AL. 2011**	Adults, intellectual disabilities	Moving from institutional life to community living; Individual	Moving to community-based accommodation	Community participation; Individual	Community inclusion	no significant effect according to study authors
				Interpersonal and Family relationship; Interpersonal	Family contact	p<0.01 (positive effect)
**FATTA ET AL., 2025**	Children, autism spectrum disorder	Relationship, marriage and family; Individual	PEERS® Italian telehealth social skills training	Social behaviour; Individual	Social Awareness, Social Cognition, Social Communication, Social Motivation, get-togethers hosted, socialization	n.s. (no significant effect according to study authors)<br>n.s. (no significant effect according to study authors)
				Social and communication skills; individual	Social knowledge	p < 0.001 ES = 0.08 (positive effect)
**YOO ET AL., 2025**	Children, autism spectrum disorder	Relationship, marriage and family; Assistive technology and rehabilitation ; Individual	NDTx-01 mobile application game	Social behaviour; Individual	Adaptive behavior,<br>Daily living skills<br>Socialization	p=0.037; (positive effect)<br>p < 0.001 (positive effect)<br>p=0.012 (positive effect)
				Social and communication skills: Individual	Social awareness, Social cognition, Social motivation Social skills	n.s.<br>(no significant effect according to study authors)
**AFSHARNEJAD ET AL., 2026**	Children, autism spectrum disorder	Relationship, marriage and family; Individual	KONTAKT™ social skills group training	Social and communication skills; Individual	Social goal attainment,<br>attainment,<br>Friendship quality,<br>social skills	(p>0.05) (no significant effect)<br>p=0.01 ES=0.67; (positive effect)<br>p=0.03 ES=0.43 (positive effect)

**JONES ET AL., 2026**	Children, autism spectrum disorder	Relationship, marriage and family; Individual	SCHOOL KONTAKT™ social skills group training	Social and communication skills; & Social behaviour; individual<br><br>Interpersonal and Family relationship; Individual	Social goal attainment,<br>social anxiety,<br><br>social skills knowledge,<br>friendship quality	p<0.001 ES=2.94; (positive effect)<br>p=0.017 ES=0.24; (positive effect)<br>p=0.007 ES=0.39 (positive effect)<br>(p=0.650) (no significant effect)
**KANG ET AL., 2024**	Children, autism spectrum disorder	Relationship, marriage and family; Individual<br><br>Recreation, leisure and sports ; Organizational ; & Community	PREP leisure and community participation	Community integration; & Community participation; Individual	Social goal attainment,<br>Participation, performance and satisfaction	p = 0.073 (no significant effect)<br>p = 0.046 ES = 0.193 (positive effect)<br>p = 0.479 (no significant effect)

ES = effect size

N.S. = No significant effect according to study authors

Appendix 8

Overview table of the included studies summarising the key characteristics of each study.

	Authors, year of publication	Sample (Participants and sample size)	Year of collecting data/ Recruitment of participants	Country	Study design	*Intervention* + stated aim of the intervention	Social inclusion outcomes	Who responded to the questionnaire concerning social inclusion outcomes:	Social inclusion measurement	Results
1	Van Veen et al. (2021)	80 adults with long-term severe non-psychotic mental disorders	2014-2016	Netherlands	RCT	*Interpersonal Community Psychiatric Treatment (ICPT)*; Improve mental and social functioning, enhance quality of life, and engage patients in recovery through structured, stage-based interaction with clinicians and their social system.	Social network; patient-perceived quantity and quality of the patient's social network	Adults with disabilities	The Social Network Map (SNM)	No significant effects for social network outcomes, including social network quality (p = 0.773) and quantity (p = 0.072), between the treatment and 'usual care' control groups.

2	Ison et al. (2010)	147 school students	N.I.	Australia	single-group before-and-after	*Disability awareness program for students;* To raise disability awareness and promote the development of supportive communities with a disability awareness program.	Acceptance of, and attitudes toward disability	Children (pupils)	Questionnaire related to questions to knowledge of disability, attitude toward disability and acceptance of people with disabilities.	Positive effect. Significant improvements in knowledge, attitudes, and acceptance of disability ($p < 0.001$).
3	H. Huang et al. (2018)	29 children with motor disabilities	2015-2017	Taiwan	Non-RCT	*Modified ride-on cars and a family-centred intervention*; Improving mastery motivation social interaction and mobility in children through structured car play activities with caregiver involvment.	Social persistence with adults, Social persistence with children	Caregivers	Revised Dimensions of Mastery Questionnaire (DMQ 18)	No effect. No significant differences between the treatment and control groups in terms of social persistence with adults ($p = .88$) or social persistence with children ($p = .86$).
4	Green et al. (2022)	248 children with autism spectrum disorder	2017-2018	England	RCT	*PACT-G intervention*; Caregiver-mediated	Social communicatio n and social skills	Researchers, parents	Autism Diagnostic Observation Schedule-	Mixed results. No significant treatment effect on social

						intervention using video feedback to enhance caregiver awareness and child communication and social interaction skills.			second edition (ADOS-2) and parent-rated Social Communication Questionnaire (SCQ-lifetime), Brief Observation of Social Communication Change (BOSCC) and dyadic social interaction between child and adult	communication (p = 0.85), restricted behaviours (p = 0.74), on language abilities (p = 0.67) and adaptive behaviour (p = 0.88) between the treatment and treatment-as-usual groups. However, treatment significantly improved synchronous responses from parents (p < 0.0001) and Learning Support Assistants (p = 0.0002), increasing child communication (p = 0.0002, p = 0.0054) and reducing autism symptoms at home (p = 0.022), though not in

										educational settings (p = 0.073).
5	Y.-C. Chou et al. (2011)	68 adults with intellectual disabilities	2009-2010	Taiwan	Non-RCT	*Active Support Training (AST)*; Support individuals with intellectual disabilities in meaningful activities through Active Support Training (AST) for supervisors and staff to improving engagement and participation of people with disabilities.	Community inclusion, contact with friends and family	Direct care workers (support staff) on behalf of the residents.	The Index of Participation in Domestic Life (IPDL); the Index of Community Involvement-Revised (ICI-R); the Social Network Index	Mixed results. A significant positive effect for community inclusion (p < 0.001), between the intervention group and the control group. However, social networks with family (p = 0.01, p = 0.03, p = 0.01) and friends (p = 0.00, p = 0.46, p = 0.00) significantly declined over the three time points in the intervention group, while no significant changes in the control group (p = 0.96, p = 0.95).
6	Yoo et al. (2014)	55 children with autism	N.I.	South Korea	RCT	*Korean Version of the PEERS® (Program for*	Social communicatio n, social	Children with disabilities,care givers and	Autism Diagnostic Observation	Mixed results. Significant positive effects

		spectrum disorder				*the Education and Enrichment of Relational Skills);* Parent-assisted social skills training program for adolescents with autism spectrum disorder.	interaction, social skills	members of the research team	Schedule (ADOS); Test of Adolescent Social Skills Knowledge—Revised (TASSK-R); Korean Version of the Social Skills Rating System (K-SSRS); Social Communication Questionnaire (SCQ), Current Form; Social Responsiveness Scale (SRS)	on social communication ($p < 0.01$), interpersonal relationships ($p < 0.01$), and social skills knowledge ($p < 0.01$), with improvements in play/leisure time ($p < 0.01$), coping skills ($p < 0.01$), hosted peer gatherings ($p = 0.01$), and peer interactions ($p = 0.06$). Reductions in autism symptoms ($p < 0.01$; $p = 0.01$), social withdrawal ($p = 0.02$), and internalizing problems ($p < 0.01$). However, the Social Skills Rating System (SSRS) total score decreased significantly ($p = 0.03$), which

										assesses social competence, including assertion, cooperation, empathy, and self-control.
7	Willis et al. (2018)	111 children with cerebral palsy, intellectual disability, other neurological and neuromuscular disorders, pervasive and specific developmental disorders	N.I.	Norway	single-group before-and-after	*Goal-directed, family-centred intervention;* To modify task/environment characteristics through goal-directed activities parent involvement, and community to facilitate participation in physical activities and participation.	Achieving individualized participation goals	Children with disabilities;Parents	The Canadian occupational performance measure (COPM); Goal attainment scale (GAS)	Positive effect. Significant improvements in children's ability to achieve their personally identified goals related to physical activity participation, with a large effect size ($p < 0.01$; $r = 0.74$). These improvements were maintained at 12 weeks follow-up, with 32% of children achieving their participation goals.
8	Kiegaldie et al. (2023)	48 adults with disabilities	N.I.	Australia	Non-RCT	*Integrated Practical Placement (IPP);*	Work skills and motivation to	Adults with disabilities supervisors	Self-reporting survey of personal	Mixed results. Significant improvements

						Supported internship model offering work experience, coaching, and skill development for students with disabilities.	engage with work related tasks		attributes and work skills.	in self-assessed work skills during the first internship (p = 0.001) and work readiness during the first rotation (p = 0.046), compared to the comparison group. However, these outcomes were not found after the third placement. Additionally, motivation to engage with work-related tasks increased significantly (p = 0.048) after the third placement.
9	Afsharnejad et al. (2022)	90 children with autism spectrum	2018-2019	Australia	RCT	*KONTAKT® social skills group training;* Improving communication, social interaction skills, problem-solving, and self-confidence	Achieving personally meaningful goals, social skills	Children with disabilities and parents	Goal Attainment Scaling (GAS); Social Responsiveness Scale—Second Edition (SRS-2); the Social Interaction Anxiety Scale (SIAS); the	Positive effect. Positive impact on achieving personally meaningful social goals, with significantly greater progress in intervention

						in autistic youth through structured intervention.			Emotion Regulation and Social Skills Questionnaire (ERSSQ)	group compared to the control group (p = 0.04, ES = 0.35). Additionally, lower scores on the social anxiety domain in intervention group at follow-up (p = 0.03, ES = 0.47) and improved social behaviours (p = 0.03, ES = 0.20).
10	Sheridan et al. (2015)	107 adults with enduring mental illness	2007-2011	Ireland	RCT	*Supported socialization*; Improving social functioning and reducing isolation by matching participants with community volunteers for social activities.	Social functioning and social and emotional loneliness	Adults with disabilities,volunteers and clinicians	Social Functioning Scale (SFS); Social and Emotional Loneliness Scale for Adults–Short Form (SELSA-S)	No effect. No significant differences in the social, family, or romantic loneliness scores between the intervention and control groups (Social p = 0.36, Family p = 0.27, Romantic p = 0.90). No significant differences in social functioning across the

										groups in terms of independence, recreation, pro-social behaviors, and overall social functioning (all p-values > 0.05).
11	Wang et al. (2015)	51 adults with chronic stroke	2011-2013	Taiwan	RCT	*Caregiver-mediated, home-based intervention (CHI);* Improve physical functioning and social participation in after hospital discharge through personalized rehabilitation and caregiver support	Social participation, activities of daily living	Adults with disabilities	The Stroke Impact Scale (SIS)	Positive effects. Significant improvement in social participation (p = 0.019) and activities of daily living/instrumental activities of daily living (p = 0.022), while no significant change in control group.
12	Tarrant et al. (2021)	41 adults with aphasia post-stroke	2017-2018	United Kingdom (England)	RCT	*Singing for People with Aphasia (SPA) Intervention;* Promote social participation, well-being, and quality of life through	Social integration	Adults with disabilities and parents	The modified Reintegration to Normal Living Index (mRNLI)	No effect. No significant difference for personal integration between the two groups over time (p = 0.22).

						participation in singing groups.				
13	Borgen et al. (2023)	120 adults sustained a traumatic brain injury	2018-2020	Norway	RCT	*Tailored and home-based goal-oriented intervention*; Enhance quality of life and social engagement through a goal-oriented rehabilitation program with personalised support..	Social participation	Adults with disabilities	The Participation Assessment With Recombined Tools–Objective (PART-O)	No effect. No significant effect on social participation between the control group and the intervention group over time (P = 0.29).
14	Holeva et al. (2024)	51 children with autism spectrum disorder	N.I.	Greece	RCT	*Robot-Assisted Psychological Intervention;* Improve social skills e.g. self-regulation, inhibition control and cognitive skills using a social-humanoid robot.	Prosocial behavior, peer relationship and interaction	Parents, teachers and medical staff (psychologist, paediatrician)	The Achenbach System of Empirically Based Assessment (ASEBA); the Strengths and Difficulties Questionnaire (SDQ); and Autism Diagnostic Interview–Revised (ADI-R)	Mix results. No significant differences in and peer relationships (p=4.66) in prosocial behaviour (p = 0.098) between the groups over time. A significant positive effect for speech-based social interaction (p=0.003) and gesture-based social

										interaction (p=0.039) and no significant interaction for eye contact (p = 0.056).
15	Modungo et al. (2010)	24 adults with Parkinson disease	N.I.	Italy	RCT	*Theatre workshop rehabilitation program*; Reduce clinical disability and improve quality of life in Parkinson's disease patients through theatre therapy focusing on social interaction and body control.	Social support	Adults with disabilities	Parkinson's Disease Quality of Life (PDQ39) Scale	Positive effect on social support (p < 0.001) between the control and the intervention group over time.
16	Timpson et al. (2018)	1845 adults with traumatic brain injury	2008-2011	United States of America	single-group before-and-after	*Advanced Theory (AdvTx)*; Improve traumatric brain injury rehabilitation outcomes like, community participation and life satisfaction by providing	Community participation, social relations, going out into the community	Adults with disabilities	The Participation Assessment with Recombined Tools-Objective-17 (PART-O)	Mixed results. Positive impact on community participation (p < 0.001) but no impact on going out into the community (p=0.072) and social relationships (p=0.227). No

						advanced therapy with early high-level tasks for better functional gain.				significant impact of severity of disability on community participation (p=0.76), going out into the community (p=0.072) and social relationships (p=0.66).
17	Chou et al. (2019)	23 children and adults with Rett syndrome	N.I.	Taiwan	Non-RCT	*Music therapy*; Improve attention, perception and communication skills Rett syndrome patients through group-based neurological music therapy with caregivers.	Socialization, interpersonal relationships, social and communication skills	Parents and trained reviewer	The Vineland Adaptive Behavior Scales (VABS); Rett Clinical Severity Scale (RCSS), and Rett Syndrome Motor Behavioral Assessment (RMBA)	Mixed results. Positive impact on socialization (p<0.01), interpersonal relationship (p<0.005), social interaction skills (p<0.01) and communication (p<0.05). No significant differences between control and intervention group in daily living skills.
18	Okajima et al. (2021)	39 children with autism	2012-2017	Japan	Non-RCT	*Social skill training and parenting*	Social behaviour, social	Parents and teachers	Social Responsiveness Scale-2 (SRS-2)	No significant tendency for improvement

		spectrum disorder				*training;* Enhance social interaction skills through social skills training, follow-up evaluations, and parental feedback sessions.	communication, social motivation, social awareness			social awareness (n.s.), social cognition (n.s.), social communication (n.s.), social Motivation (n.s.), and autistic mannerisms (p < 0.10) between treatment and treatment as usual group.
19	Caliendo et al. (2021)	84 children with autism spectrum disorder	2020-2021	Italy	single-group before-and-after	*A neuro-psychomotor treatment for children with Autism Spectrum Disorder;* Enhance developmental potential of children with autism spectrum disorder through individualized, play-based neuro-psychomotor therapy	Behaviours in social relations	Trained child therapists	Behaviors in social relations (RELSOC)	Positive effect on behaviors in social relations (p> 0.0001) between baseline and follow-up.

						targeting multiple skills (social, communication, motor, cognitive etc.)				
20	Bogner et al. (2019)	1843 adults with traumatic brain injury	2008-2011	United Stated of America	Non-RCT	*Family attendance during therapy session*; Improve community participation, functional independence and subjective well-being by involving family members in at least 10% of therapy session time during rehabilition.	Community participation, social relations, going out into the community	Adults with disabilities and parents	The Participation Assessment for Recombined Tools-Objective-17 (PART-O-17)	Mixed results. Positive impact on going out into the community (p= 0.02), no significant effect on social relations (p=0.29) and community participation (p=0.08) between intervention and control group.
21	Kendall et al. (2007)	1000 adults sustained a stroke	N.I.	Australia	RCT	*The Chronic Disease Self-Management Course*, Promote psychosocial recovery through group education focusing self-management.	Social roles, family roles	Adults with disabilities	The Stroke Specific Quality of Life scale (SSQOL)	No effect on social roles (n.s.) between intervention and control group.

22	Slaman et al. (2015)	57 adults with cerebral palsy	2009-2011	Netherlands	RCT	*Active lifestyle and sports participation intervention*; Improve physical behaviour, physical fitness, and sports participation through counselling, training, and identifying accessible sports facilities	Social participation, social support	Adults with disabilities	The short version of the Life Habits Questionnaire (LIFE-H 3.0); the Social Support for Exercise Behaviour Scale	No significant effect for participation in daily activities (95%CI: −0.22 [−0.50, 0.07]), social roles. (95%CI: 0.04 [−0.60, 0.53]) and social support (95%CI: −0.08 [−1.99, 1.83]).
23	Tateno et al. (2021)	27 children with autism spectrum disorder	2018-2019	Japan	single-group before-and-after	*The Early Start Denver Model*; Reduce the severity of autism spectrum disorder symptoms, particularly in the areas of language and social skills, through comprehensive early intervention that incorporates the principles of	Interpersonal relationships, socialization and verbal activities	Caregiver, psychologists and research team members	The Kyoto Scale of Psychological Development (K-test)	Positive impact. Significant positive impact on interpersonal relationships, socialization and verbal activities (p=0.0094) between baseline and 12 weeks follow-up.

						Applied Behavioural Analysis.				
24	Taylor et al. (2016)	703 adults with chronic musculoskeletal pain	2011-2012	United Kingdom	RCT	*Community-Based, Nonpharmacological Group Intervention for Chronic Musculoskeletal Pain (COPERS)*; Improve understanding, mood and behaviour to increase self-efficacy in managing chronic pain through a cognitive behavioural group course focusing on pain acceptance, goal setting and relaxation techniques.	Social integration and Support, having a supportive social network or reliable people to rely on	Adults with disabilities	Health Education Impact Questionnaire (heiQ) social integration and support	Positive impact on social integration and support (95%CI: 0.8 [0.4 to 1.2]) between intervention group and control group after follow up.
25	Cheung et al. (2021)	74 children with autism spectrum disorder	2017-2018	Hong Kong	Non-RCT	*School-Based social cognitive intervention*; Improve the social participation,	Social skills, achieving client-centred social goals	Parents	The Chinese version of the Social Skills Improvement System	Positive effect. Large effect size of social skills (ES= 1.03, p=0.005) and of mediate effect

						social skills and cognitive development of children with autism spectrum disorder through the integration of visually scaffolded materials, social skills role play and games.			Rating Scales (SSIS-RS-C); Goal Attainment Scaling (GAS)	on achieved social goals (ES=0.052 p=.047).
26	Rodríguez -Rivas et al. (2021)	40 children and adults with mental disorders	N.I.	Chile	RCT	*Virtual program to reduce stigma among university students toward people with mental disorders;* reducing the stigma of mental disorders through online e-contact, standardised patient simulations and project-based learning for	Stereotypes toward schizophrenia and social distancing and stigma toward people with mental disorders	University students	Questionnaire on Student Attitudes Toward Schizophrenia (QSAS); Attribution Questionnaire (AQ–27) ; Learning Strategies Assessment Scale	Positive effect**:** A significant positive effect in intervention group between baseline and follow-up in reducing stigma towards schizophrenia social distancing (p = 0.004, d = 1.11) and people with mental disorders (p = 0.000, d = 2.33). In the control group, no effect between baseline and

						university students.				follow-up for both scales.
27	H.-H. Huang et al. (2018)	29 children with motor disabilities	2015-2017	Taiwan	Non-RCT	*Ride-on car training combined with an adult-directed, social interaction program;* improve mobility, social function, and reduce parenting stress by using ride-on car training with social skills for young children.	Social functioning	Parents	Pediatric Evaluation of Disability Inventory (PEDI)	Positive effect on social function between intervention and control group over time between the groups (ES=0.123, p=0.03).
28	Myles et al. (2000)	58 adults with learning disabilities	1995-1997	United Kingdom	single-group before-and-after	*Resettlement across different models of community accommodation*; Enhance social integration and community involvement of clients with learning disabilities in community settings by transitioning them from	Community involvement	Adults with disabilities and/ or their key workers	The index of community involvement (ICI)	Significant impact on community involvement (p< 0.005) between baseline and follow-up. (Myers et al. 1997)<br><br>Myers F., Ager A., Green A., Kerr P., Myles S. & Matheson J. (1997) *Moving Home. Community Integration and Costs Associated*

						institutionalized care to various community accommodation models.				*with Different Models of Accommodation for Adults with Learning Disabilities*. A Report
29	Adamus et al. (2022)	58 adults with severe mental illness	2019-2021	Switzerland	RCT	*Independent Supported Housing*; improve social inclusion for individuals with severe mental illness through independent supported housing, providing personalized housing support and rehabilitation in a community setting.	Social inclusion and participation; social engagement, interpersonal behaviour, pro-social and recreational activities, leisure, and occupational participation.	Adults with disabilities	The German version of the Social Functioning Scale (SFS)	No effect on social inclusion and participation between intervention and control group over time (95% CI: (6.28 [–0.08 to 13.35]).
30	Heppe et al. (2020)	76 children and adults with a visual impairment	2015-2017	Netherlands	RCT	*Community-based mentoring program;* promote social participation in young people with visual impairment by providing	Participation, social network size, social support	Adults with disabilities	The Visual Activity and Participation (VAP) scale; Social participation composite score; the Social Network Map; Personal	No significant effect on social participation (VAP scale; 95%CI: −0.30, 0.21), social participation composite score (95%CI: −0.79,

						mentoring sessions, focusing on activities such as social interaction and goal setting.			Network List (PNL); Multidimensional Scale of Perceived Social Support (MSPSS); Satisfaction with social support was measured with two items	0.23) degree of peer activity (95%CI: −0.32, 0.03; d=0.27), social network size (95%CI: −1.42, 6.39), perceived peer support (95%CI: −7.32, 23.01), perceived parent support (95%CI: −14.44, 16.86), and online social support (95%CI: −0.35, 0.21) between the mentoring conditions and the care-as-usual group, as well as between the mentor and no-mentor groups. However, a positive effect in satisfaction with social support between the mentor and no-mentor groups

										(95% CI: 0.02–0.49, d = 0.38).
31	Sanches et al. (2020)	188 adults with severe mental illnesses	2014-2017	Netherlands	RCT	*Boston University Approach to Psychiatric Rehabilitation (BPR)*; improve social participation in individuals with severe mental illness by supporting rehabilitation goal achievement in housing, education, work, and social contacts through regular sessions with trained professionals.	Social functioning, social participation,	Adults with disabilities	The Occupation and Employment subscale of the Birchwood Social Functioning Scale (SFS_OE); the Dutch National Societal Participation Ladder; the Birchwood Social Functioning Scale (SFS)	No effect was found over time between intervention and control group for social functioning (p=0.234), social participation (p=0.893) and hours of participation (p=0.456).
32	Lorant et al. (2019)	1407 adults with severe mental illness	2014-2015	Belgium	Non-RCT (case-control design using non-randomised comparison areas)	*Mental health care delivery reform in Belgium (2014-2015)*; strengthen community-based care, improve	Social integration	Adults with disabilities	Objective social outcomes index (SIX)	No impact on social integration (p=0.20).

						continuity, reduce hospitalisation and promote social inclusion of patients by reforming mental health care to improve continuity of care for patients with severe mental illness through the establishment of service networks.				
33	Baumgardt et al. (2020)	498 adults with chronically mental illness	2015-2017	Germany	single-group before-and-after	*Community-based clinical social work*; Improve capabilities in individuals with chronic mental disorders by fostering empowerment and social inclusion through psychosocial interventions like psychoeducation, life skills	Social inclusion, social relationships, psychosocial functioning	Adults with disabilities	German measure for participation and social inclusion for use in people with a chronic mental disorder “Fragebogens zur Erfassung sozialer Inklusion und Partizipation” (F-INK); the questionnaire Empowerment in the Psychiatric	Mixed results. Positive effect on social inclusion (ES = 0.158, p = 0.03), with no significant effect on social relationships (p = 0.381) and psychosocial functioning (p = 0.183).

						training, and family interventions			Treatment Process for People with Schizophrenic Disorder (EPAS); Global Assessment of Functioning Scale (GAF)	
34	Chou et al. (2011)	49 adults with intellectual disabilities	2005-2007	Taiwan	single-group before-and-after	*Moving into a community-based accommodation*; improved adaptive behaviour, quality of life, community inclusion and family contact following transition from institutional or family homes to small community-based settings.	Community inclusion, family contact	Adults with disabilities	Use of Community Facilities Scale (UCFS); Family Contact Item	Mixed effects. No significant effect on community inclusion, while a positive effect on family contact ($p < 0.01$).
35	James et al. (2021)	135 adults with learning disabilities	2015-2019	United Kingdom	single-group before-and-after	*Promote the Vote;* To increase the participation of people with learning	Election participation	Adults with disabilities	The survey tool to collect data in the 2015 UK Parliamentary Election.	Positive effect on people registered to vote ($p = 0.0012$), people aware of their

						disabilities in elections by providing information about their voting rights and practical support in registering and voting.				right to register and participate in voting (p = 0.0035) and number of people who voted (p < 0.001).
36	Hassiotis et al. (2018)	245 adults with intellectual disabilities	2013-2015	United Kingdom	RCT	*Positive Behaviour Support (PBS*); reduce challenging behaviour and improve quality of life of people with intellectual disabilities through PBS training for support, including functional assessment, prevention strategies, response strategies and ongoing therapist mentoring.	Participation in activities in the community	Family carer or paid carer	Guernsey Community Participation and Leisure Activities Scale (GCPLA)	No effect on frequency of activities (95%CI: -0.57-1.74).

37	Chong et al. (2024)	48 children and adults with intellectual developmental disabilities	N.I.	Singapore	single-group before-and-after	*Our Lives, Our Voices (OLOV) programme*; promote self-advocacy for persons with intellectual developmental disabilities by enhancing autonomy, confidence, communication, and rights awareness through training, role-playing, and real-world advocacy application.	Communication skills	Family members, primary caregivers and the respective support staff	A self-constructed 19-questions questionnaire to collect data on: awareness of rights, communication skills, confidence to speak up and ability to practice skills.	Positive effect on communication skills with a large effect size ($p<0.01$, $d=0.813$).
38	**Fatta et al. 2025**	44 children with autism spectrum disorder	N.I.	Italy	RCT	*PEERS® Italian adaptation delivered via telehealth;* to improve social skills, social knowledge, and social performance in autistic adolescents through structured group-based	Social knowledge, social performance (get-togethers hosted and attended), global social competence	Children with disabilities, parents, teachers	Social Responsiveness Scale (SRS) parent and teacher version ; Social Initiative and Social Reciprocity scales (TASSK-R; QSQ-R)	Mixed results. Significant improvement in social knowledge between treatment and waitlist group ($p<0.001$; $\eta^2p=0.78$). Significant increase in get-togethers hosted between

						social skills training with parallel parent coaching sessions.				treatment and waitlist group ($p<0.001$). No significant differences between groups on parent-rated SRS total (p=n.s.), teacher-rated SRS total (p=n.s.), and quality of socialization ($p=0.168$).
39	Martínez-Tur et al. 2025	435 adults with intellectual and developmental disabilities	2022-2023	Spain	RCT	*Customized Employment (CE);* to improve competitive integrated employment and social inclusion through individualized job matching between the person's strengths and interests and employer needs across four phases: discovery, planning,	Visibility to others due to employment activities; sense of belonging to a neighbourhood or community; number of new places visited for employment	Adults with disabilities; support staff	Single-item validated scales	Positive effects on visibility to others between intervention and control group ($p<0.01$; ç^{2}=0.02) and sense of belonging to community ($p<0.001$; ç^{2}=0.03). Professionals additionally reported significant improvement in number of new places visited for employment

						negotiation, and support				(p<0.001; ç²=0.19). No significant differences between groups in outcomes by severity level of intellectual disability (p>0.05).
40	Yoo et al. 2025	38 children with autism spectrum disorder or social communication disorder	2023	South Korea	RCT	*NDTx-01 (mobile application game);* to improve social communication skills in adolescents with ASD or SCD through gamified social skills training scenarios focusing on school peer relationships, modelling verbal and nonverbal social communication	Adaptive behavior (communication, daily living skills, socialization); social responsiveness; social communication	Children with disabilities, parents	Korean Vineland Adaptive Behavior Scales-II (K-VABS-II); Social Responsiveness Scale-2 (SRS-2); Korean Social Communication Questionnaire (K-SCQ); Korean Version of the Social Skill Rating System (K-SSRS)	Mixed results. Significant improvement in adaptive (p=0.037), in daily living skills (p<0.001) and socialization (p=0.012) between intervention and control group. Significant reduction in restricted and repetitive behaviours (p=0.037). No significant between-group differences on SRS-2 total score (n.s.), social

										communication (n.s.), social awareness (n.s.), social cognition (n.s.), social motivation (n.s.) and social skills (n.s.).
41	Chien et al., 2024	50 children with developmental disabilities	2021 - 2023	Hong Kong	RCT	*Occupational Performance Coaching (OPC);* to enhance community participation in young children with developmental disabilities by guiding parents through a collaborative goal-setting and performance analysis process to develop and implement individualised strategies	Goal-specific community participation performance and satisfaction; community participation frequency and involvement	Parents	Canadian Occupational Performance Measure (COPM); Young Children's Participation and Environment Measure (YC-PEM)	No effect. No significant between-group differences on goal-specific community participation performance (p=0.355) or satisfaction (p=0.611), community participation frequency (p=0.066), or community participation involvement (p=0.954).
42	Kang et al., 2024	21 children with autism spectrum disorder and their parents	2018-2020	Taiwan	RCT	*PREP (Pathways and Resources for Engagement and Participation);*	Goal-specific participation performance and satisfaction;	Children with disabilities, parents	Canadian Occupational Performance Measure (COPM); Goal	Mixed effect. No significant between-group differences on COPM

						to support autistic children to participate in self-chosen leisure and community activities by reducing environmental barriers and modifying activity demands through individualized therapist coaching sessions with parents	goal attainment; parent empowerment and efficacy		Attainment Scaling (GAS); Parent Empowerment and Efficacy Measure (PEEM)	satisfaction (p=0.479), GAS T-score (p=0.073), or parent efficacy (PEEM; p=0.291 at 12 weeks; p=0.158 at 24 weeks). Positive effect for COPM performance (p=0.046, ES = 0.193).
43	Afsharnejad et al. 2026	84 children with autism spectrum disorder	2020-2023	Australia	RCT	*KONTAKT™ social skills group programme;* to support children with autism spectrum disorder in achieving their personally meaningful social goals through structured group-based social skills	Personal social goal attainment; friendship quality; social skills	Children with disabilities, parents	Goal Attainment Scaling (GAS); LERID Friendship Scale; Social Skills Group Assessment (SSGA)	Mixed results. No significant between-group differences on personal social goal attainment (p>0.05) at primary endpoint. Significant improvement in friendship quality (p=0.01; ES=0.67). No significant differences on

						training combining implicit and explicit educational strategies including role-play, discussion and real-life practice				social skills (p>0.05) at primary endpoint. At 1-year follow-up, significant improvement in social skills (p=0.03; ES=0.43).
44	Jones et al., 2026	69 children with autism spectrum disorder	2023–2024	Australia	single-group before-and-after	*SCHOOL KONTAKT™;* a manualised social skills group training programme delivered by trained teachers in a school setting, combining structured and unstructured components including role-play, group activities, and themed discussions; aimed at improving social participation outcomes for autistic high school students	Personal social goal attainment; social anxiety; knowledge of social skills; friendship quality; social responsivenes s; school inclusiveness	Children with disabilities, parents, teachers	Goal Attainment Scaling (GAS); Social Interaction Anxiety Scale (SIAS); KONTAKT Social Skills Questionnaire (KSSQ); LERID Friendship Scale; Social Responsiveness Scale 2nd edition (SRS-2; parent- and teacher-reported); Emotion Regulation Checklist (ERC; parent-reported); INCLUSIO (teacher-reported)	Mixed results. Significant improvement in personal social goal attainment (p<0.001; ES=2.94) and in social skills knowledge (p=0.007; ES=0.39). Significant reduction in social interaction anxiety (p=0.017; ES=−0.24) and in autistic traits by parent-reported (p<0.001; ES=−0.50) and teacher-reported

						through individually meaningful goal setting and social skills practice in a naturalistic school environment				(p=0.009; ES=−0.40) assessment. No significant change in friendship quality (p=0.650) and in overall school inclusiveness (p=0.096).
45	Chan et al., 2023	95 children with autism spectrum disorder	N.I.	Hong Kong	RCT	*The Transporters App;* a home-based animated video intervention delivering episodes via a mobile App; aimed at improving emotion recognition in young autistic children, with or without ADHD, through illustrative stories linking facial expressions to social contexts	Emotion recognition (vocabulary and recognition)	Children with disabilities	Emotion Vocabulary Task (EVT); Emotion Recognition Task levels 1–3 (ERT1, ERT2, ERT3)	Positive effect. Significant differences on emotion recognition ($p<0.001$; $\eta p^2=0.445$)

46	Hsiao et al., 2024	21 children with autism spectrum disorder	N.I.	Taiwan	RCT	*PEERS® (Program for the Education and Enrichment of Relational Skills);* a parent-assisted structured social skills training programme with concurrent adolescent and parent groups; aimed at reducing school bullying and enhancing social function among autistic adolescents	School bullying (victimization, perpetration); social challenges; social skills knowledge; social skills performance (peer conflicts)	Children with disabilities, parents	Chinese version of the School Bullying Experience Questionnaire (C-SBEQ); Social Responsiveness Scale (SRS); Test of Adolescent Social Skills Knowledge (TASSK); Quality of Socialization Questionnaire–Parent (QSQ-P)	Positive effect. Significant reduction in overall school bullying ($p<0.001$; $\eta^2=0.59$), victimization ($p<0.001$; $\eta^2=0.66$), perpetration ($p=0.026$; $\eta^2=0.25$), social challenges ($p<0.001$; $\eta^2=0.60$), peer conflicts ($p<0.001$; $\eta^2=0.71$), and improvement in social skills knowledge ($p<0.001$; $\eta^2=0.83$) at 4-month follow-up.
47	Imamoto et al., 2025	90 adults with psychiatric conditions	2017-2021	Japan	single-group before-and-after	*Individualized occupational therapy programme based on the Occupational Therapy Intervention Process Model*	Occupational performance; social interaction skills	Adults with disabilities	Canadian Occupational Performance Measure (COPM); Assessment of Motor and Process Skills (AMPS);	Positive effect. Significant improvements in occupational performance (COPM performance: $p<0.001$; $d=0.79$) and

						(OTIPM); a client-centred, occupation-based programme; aimed at improving occupational performance and social interaction skills to support community reintegration among adults with psychiatric conditions			Evaluation of Social Interaction (ESI)	social interaction skills (ESI: $p<0.001$; $d=0.72$).
48	Alecu & Onea, 2025	36 children with autism spectrum disorder	2022-2024	Romania	single-group before-and-after	*Therapeutic swimming programme;* an aquatic intervention of combining ABA strategies, sensory integration, and positive reinforcement; aimed at improving well-being, attention, learning, physical activity, and social	Self-confidence and social behaviors	Parents	Parent Perception Questionnaire (PPQ-Autism-Swim; non-validated, study-specific tool)	Positive effect. Significant improvements in self-confidence and social behaviors ($\eta^2=0.81$; $p < 0.001$).

						functioning in autistic children following ABA therapy				
49	Reddy et al., 2025	57 adults with subacute stroke	2023-2024	Saudi Arabia	RCT	*Transcranial direct current stimulation (tDCS) combined with gait-oriented motor training* (treadmill, overground walking, balance and functional mobility exercises; aimed at improving disability, quality of life, motor function, and balance in subacute stroke patients	Social participation	Adults with disabilities	Stroke Impact Scale (SIS)	Positive effect. Significant improvements in social participation (SIS participation domain; p=0.001; d=0.67)
50	Stanley et al., 2025	39 adults with mild to moderate mental illness	2023-2024	Australia	single-group before-and-after	*Nature Scripts;* a curated group-based nature prescribing programme including nature journaling, yoga, tree planting, bushwalks and	loneliness; social connectedness ; feeling part of community	Adults with disabilities	Contact with people in community, single item scale	No effect. Community contact improved in a positive direction but did not reach statistical significance at either post-

						nature photography; aimed at improving wellbeing, mental health, social connectedness and pro-environmental behaviour in young adults with mild to moderate mental illness				intervention (p=0.072) or follow-up (p=0.058).

Appendix 9

Classification of social inclusion interventions across included studies

	Individual*	Interpersonal*	Organizational*	Community*	Socio-political*
Personal-Assistance**	(Sheridan et al., 2015); (Adamus et al., 2022) ; (Martínez-Tur et al. 2025)	(Chou et al, 2011)			
Relationship, marriage and family**	(Yoo et al. 2014) ; (Afsharnejad et al., 2022) ; (Holeva et al., 2024) ; (Okajima et al, 2021) ; Caliendo et al. (2021) ; (Tateno et al., 2021) ; (Huang et al.m 2018) ; (Heppe et al., 2020) ; (Sanches et al., 2020) ; (Fatta et al., 2024) ; (Yoo et al., 2025) ; (Afsharnejad1 et al., 2026) ; (Jones et al., 2026) ; (Chan et al., 2023) ; (Hsiao et al., 2024) ; (Imamoto et al., 2025) ; (Alecu & Onea, 2025)	(Ison et al., 2010) ; (Green et al. 2022) ; (Sheridan et al., 2015) ; (Yoo et al., 2014) ; (Okajima et al, 2021) ; (Bogner et al., 2019) ; (Rodríguez-Rivas et al., 2021) ; (Heppe et al., 2020) ; Chien et al., 2024) ; (Kang et al., 2024) ; (Hsiao et al., 2024)	(Ison et al., 2010) ; Hassiotis et al. 2018)		
Culture and Arts**	(Modugno et al., 2010)				
Recreation, leisure and sports**	(Tarrant et al., 2021); (Modugno et al., 2010) ; (Slaman et al., 2015) ; (Stanley et al., 2025)	(Sheridan et al., 2015)	(Kang et al., 2024)	(Kang et al., 2024)	

Justice**					
Assistive technology and rehabilitation**	(Van Veen et al., 2021); (Huang et al., 2018) : (Willis et al., 2018) ; (Wang et al., 2015) ; (Borgen et al., 2023) ; (Timpson et al. 2019) ; (Chou et al., 2019) ; (Caliendo et al., 2021) ; (Kendall et al., 2007) ; (Tateno et al., 2021) ; (Taylor et al., 2016) ; (Cheung et al., 2021) ; (Huang et al., 2018) ; (Sanches et al., 2020); (Yoo et al., 2025) ; (Imamoto et al., 2025) ; (Reddy et al., 2025)	(Willis et al., 2018); (Wang et al., 2015) ; (Bogner et al., 2019)			
Policies and programs**					(Lorant et al., 2019) : Myles et al. 2000
Supported internship + works skills	(Kiegali et al., 2023)); (Martínez-Tur et al. 2025)				
Moving from institutional life to community living	(Adamus et al., 2022); (Chou et al., 2021) ; (Myles et al., 2000)				
Promoting electoral participation	(James et al. 2021)				

1

2

Appendix 10

Classification of social inclusion outcomes across included studies

		**Individual***	**Interpersonal***	**Organizational***	**Community***	**Socio-political***
**Social****	Social identity**					
	Personal assistance**					
**Skills for social inclusion****	Social and communication skills**	(Huang et al., 2018); (Yoo et al., 2014) ; (Green et al. 2022) ; (Afsharnejad et al., 2022) ; (Holeva et al, 2024) ; (Chou et al., 2019) ; (Okajima et al., 2021) ; (Caliendo et al., 2021) ; (Tateno et al., 2021) ; (Cheung et al., 2021) ; (Huang et al., 2018) ; (Chong et al., 2024) (Baumgardt et al. ; 2020) ; (Fatta et al., 2024) ; (Yoo et al., 2025) ; (Chien et al., 2024) : (Afsharnejad et al., 2026) ; (Jones et al., 2026) ; (Chan et al., 2023) ; (Hsiao et al., 2024) ; (Imamoto et al., 2025) ; (Alecu & Onea, 2025)	(Sheridan et al, 2015)			
	Social behaviour**	(Yoo et al., 2014) ; (Holeva et al, 2024) ;(Chou et al., 2019) ; (Caliendo et al., 2021) ; (Kendall et al., 2007) ; (Tateno et al., 2021) ; (Adamus et al., 2022) (Fatta et al., 2024) ; (Martínez-Tur et al. 2025) ; (Yoo et al., 2025) ; (Jones et al.,	(Sheridan et al, 2015)			

		2026) ; (Hsiao et al., 2024) ; (Imamoto et al., 2025) ; (Alecu & Onea, 2025)				
**Broad based social inclusion and participation measure****	Social inclusion**	(Timpson et al., 2019) ; (Bogner et al., 2019) ; (Heppe et al., 2020) ; (Reddy et al., 2025)				
	Community integration**	(Yoo et al., 2014) ; (Holeva et al, 2024) ; (Martínez-Tur et al. 2025) ; (Kang et al., 2024)				
	Community participation**	(van Veen et al., 2021); (Chou et al., 2011) ; (Willis et al., 2018) ; (Kiegaldie et al, 2023) ; (Wang et al, 2015) ; (Tarrant et al, 2021) ; (Borgen et al, 2023) ; (Timpson et al., 2019) ;(Bogner et al., 2019) ; (Slaman et al., 2015) ; (Myles et al., 2000) ; (Adamus et al., 2022) ;(Heppe et al., 2020) ; (Sanches et al., 2020) ; (Lorant et al., 2019) ; (Baumgardt et al. ; 2020) ; (Chou et al. 2011) ; (James et al., 2021) ; (Hassiotis et al., 2018) ; (Martínez-Tur et al. 2025) ; (Kang et al., 2024) ; (Imamoto et al., 2025) ; (Reddy et al., 2025) ; (Stanley et al., 2025)	(Heppe et al., 2020)		(Sheridan et al, 2015)	

	Access to justice**					
**Relationships** **	Interpersonal and Family relationship**	(Chou et al, 2011); (Modungo et al., 2010) ; (Chou et al., 2019) ; (Tateno et al., 2021) ; (Lorant et al., 2019) ; (Baumgardt et al. ; 2020) ; Holeva et al, 2024) ; (Timpson et al., 2019 ; ; (Heppe et al., 2020) ; (Martínez-Tur et al. 2025); (Jones et al., 2026)	(Taylor et al., 2016); (Chou et al., 2011)			
	Peer and community relationship**		(Ison, 2010); (Rodríguez-Rivas et al.; 2021)	(Ison, 2010)		
	Violence and abuse**	(Hsiao et al., 2024)				

Appendix 11

Results of the risk of bias assessment

ROBINS-I (The Risk Of Bias In Non-randomized Studies)								
Study ID	Bias due to confounding	Bias in selection of participants into the study	Bias in classification of interventions	Bias due to deviations from intended interventions	Bias due to missing data	Bias in measurement of outcomes	Bias in selection of the reported result	Overall bias
**Ison et al. 2010**								**Serious**
**Huang et al. 2018**								**Serious**
**Chou et al. 2011**								**Moderate**
**Willis et al. 2018**								**Serious**
**Kiegaldie et al. 2023**								**Serious**
**Timpson et al. 2019**								**Moderate**
**Chou et al. 2019**								**Moderate**
**Okajima et al. 2021**								**Moderate**
**Caliendo et al. 2021**								**Serious**
**Bogner et al. 2019**								**Serious**
**Tateno et al. 2021**								**Serious**
**Cheung et al. 2021**								**Moderate**
**Huang et al. 2018**								**Serious**
**Myles et al. 2000**								**Serious**
**Lorant v2019**								**Moderate**
**Baumgardt et al. 2020**								**Serious**
**Chou et al. 2011**								**Moderate**
**James et 2021**								**Moderate**
**Chong et al. 2024**								**Serious**
**Alec et al. 2025**								**Serious**
**Imamoto et al. 2025**								**Serious**

**Jones et al. 2026**								**Serious**
**Stanley et al. 2025**								**Serious**

1

Colour key				
Low	Moderate	Serious	Not applicable	No information

Risk of Bias in randomized trials (RoB 2)

Study ID	Domain 1: Risk of bias arising from the randomization process	Domain 2: Risk of bias due to deviations from the intended interventions (effect of adhering to intervention)	Domain 3: Risk of bias due to missing outcome data	Domain 4: Risk of bias in measurement of the outcome	Domain 5: Risk of bias in selection of the reported result	Overall risk of bias
**Van Veen et al. 2021**						**Some concerns**
**Green et al. 2022**						**Some concerns**
**Yoo et al. 2014**						**Some concerns**
**Afsharnejad et al. 2022**						**Low**
**Sheridan et al. 2015**						**High**
**Wang et al. 2015**						**Some concerns**
**Tarrant et al. 2021**						**Some concerns**
**Borgen et al. 2023**						**Low**
**Holeva et al. 2024**						**Low**
**Modungo et al. 2010**						Low
**Kendall et al. 2007**						**High**
**Slaman et al. 2015**						**High**
**Taylor et al. 2016**						**High**
**Rodriguez-Rivas et al. 2021**						**Some concerns**
**Adamus et al. 2022**						**Some concerns**
**Heppe et al. 2020**						**Low**
**Sanches et al. 2020**						**Low**
**Hassiotis et al. 2018**						**Some concerns**
**Yoo et al. 2025**						**High**
**Afsharnejad et al., 2026**						**Some concerns**
**Chan et al. 2024**						**Some concerns**

**Chien et al., 2024**						**Some concerns**
**Fatta et al. 2025**						**High**
**Hsiao et al., 2024**						**Some concerns**
**Kang et al. 2023**						**Low**
**Martínez-Tur et al. 2025**						**Some concerns**
**Reddy et al. 2025**						**Low**

Colour key		
Low	Some concerns	High
