# Supplementary material for: Effectiveness of interventions to promote social inclusion of people with disabilities in high-income countries: a systematic review of quantitative evaluation studies
Source: BMJ Open. 2026 Jun 16;16(6):e117213. doi: 10.1136/bmjopen-2026-117213 (PMC13288884; doi:10.1136/bmjopen-2026-117213)
Supplement: Supplementary file 2 [file bmjopen-2026-117213.reviewer_comments.pdf]

PEER REVIEW HISTORY

BMJ Open publishes all reviews undertaken for accepted manuscripts. Reviewers are asked to complete a checklist review form and are provided with free text boxes to elaborate on their assessment. These free text comments are reproduced below.

ARTICLE DETAILS

Title (Provisional)

Effectiveness of interventions to promote social inclusion of people with disabilities in high-income countries: a systematic review of quantitative evaluation studies.

Authors

Schroeder, Manon; Weber, Germain; Suhrcke, Marc

VERSION 1 - REVIEW

**Reviewer**	**1**
**Name**	**Kumurenzi, Anne**
**Affiliation**	**University of Rwanda College of Medicine and Health Sciences, Physiotherapy**
**Date**	**03-Mar-2026**
**COI**	**None**

General comments:

The manuscript presents a comprehensive study of "Effectiveness of Interventions to Promote Social Inclusion of People with Disabilities in High-Income Countries: A Systematic Review", offering valuable insights into social inclusion interventions and outcomes across various high-income contexts. The research question is clearly defined, and the paper aims to address a gap in the literature on the effectiveness of social interventions. However, there are several areas in the methodology, results, and discussion sections that require revision for clarity, consistency, and scientific rigor.

Major revisions:

Participants: The inclusion of both children and adults in the study raises questions about the comparability of the interventions and outcomes. It is unclear how the study accounted for potential differences in age, disability type, or intervention response across these two groups. The authors should either provide more details on how the data was disaggregated by age or consider justifying the inclusion of both groups in the same analysis.

Interventions: The manuscript mentions that interventions may differ, but it remains unclear how the diversity of interventions and their outcomes were handled in the analysis. The authors should describe how these variations were treated in their synthesis. Were interventions analyzed separately

for each age group, or were they combined?

Exclusion: The exclusion of studies not conducted in high-income countries is mentioned, but this is obvious since the review focused on high-income countries.

Results: The results section lacks clarity. For instance, the statement "Fifteen studies reported significant positive effects, 12 no effect" is vague. What specific outcomes were these studies assessing? What criteria were used to define "positive" or "no effect"? The authors should provide more detailed descriptions of the outcomes assessed, as well as the specific interventions that led to positive or no effects. A table summarizing the effects of different interventions would enhance the clarity of this section.

Narrative synthesis and statistical analysis: The results are narratively synthesized, but there is limited mention of statistical analysis or how the studies were quantitatively assessed (mainly in the abstract). For the main documents, if available, the authors should include statistical analyses, such as effect sizes, confidence intervals, or p-values, to provide more robust evidence of the effectiveness of the interventions.

Minor revisions:

Methodology: Some sections of the methods, particularly the search strategy, contain inconsistencies. For example, the abstract mentions eight databases, but the methods section mentions only seven. Additionally, the dates covered in the search are not consistent in the abstract and main document.

Bias assessment: The paper mentions a "risk of bias" section but does not provide sufficient detail on how the risk of bias was assessed for each included study. The authors should elaborate on the tools or criteria used for bias assessment. Improve the table in the appendix with the bias ratings for each study would improve the transparency of the review process.

There are several grammatical and structural issues, the authors should carefully proofread the manuscript for grammar and structure.

Discussion:

Interpretation of results: While the results are synthesized, the discussion does not sufficiently address the implications of the findings or how they relate to existing literature. The rationale behind the findings should be more thoroughly explored. The authors should explore deeper into the possible reasons for the lack of consistent outcomes across studies. They should also discuss the implications for practice more explicitly, suggesting how the findings can inform future interventions.

Some sentences in the discussion are bolded, but the reason for this emphasis is unclear.

Conclusion:

In conclusion, while the manuscript addresses a significant topic and has the potential to contribute meaningfully to the field, it requires revisions to improve clarity it. The study would benefit from clearer distinctions between age groups, a more transparent explanation of the data synthesis methods, and a stronger discussion of the results. I recommend a thorough revision to address the concerns raised above.

**Reviewer**	**2**
**Name**	**Wark, Stuart**
**Affiliation**	**University of New England, School of Rural Medicine**
**Date**	**06-Mar-2026**
**COI**	**None**

This manuscript reports on a systematic review of the research quantum regarding the effectiveness of interventions that support the better inclusion of people with disabilities. The demographic focus of the review is on high-income countries. I believe that the chosen databases are appropriate for the question, and the risk of bias assessed in a suitable manner. There does seem to be some inconsistency in the usage of US and UK spelling (i.e. words with 'ize' like synthesizes versus words with "iour" like behaviour) but this is easily remedied. The authors are to be commended for their consideration of this topic area, as it is not normally explored within more mainstream health journals.

I do have some initial observations and comments for the authors to consider, and these are detailed below:

- I was intrigued that the authors decided to exclude all qualitative papers from the review. This is potentially appropriate if the plan is to do a meta-analysis (for example), but I wasn't entire sure of the rationale for excluding personal testimony and/or feedback from the primary participants/beneficiaries of any intervention? I recognise the conceptual desire for only including evaluation studies, but does that approach necessarily exclude all qualitative papers? This is not a major issue, and a brief explanation as to why this decision was made would assist the reader to understand the context.

- The actual search was undertaken nearly two years ago (manuscript submitted to journal in late January 2026). I understand that review papers take a lot of time to develop, but this timeframe is now significant. Is there likely to be any more recent research that should be included? This is highly relevant as most of the papers identified occurred within the most recent time period (18 papers from 2020-24) and therefore it seems probable that more papers will have emerged that could add substantially to the review.

- The Abstract notes eight databases being searched, but then only lists seven. The Methods nominates seven, so I assume that the 'eight' is just a typo?

- The choice to not have at least two reviewers independently evaluate all papers at both the title/abstract screen stage and then definitely at the full-text stage is always slightly concerning to me. Again, I understand the rationale for this choice due to time efficiency, but this does come at the increased risk of errors. It would be good, at a minimum, to confirm that the second reviewer was truly independent (i.e. they were unaware of other reviewer's decisions) at each stage of their sample screening, whether all the full-text exclusions were verified by the second reviewer, and

whether the process used to resolve disagreements included referral to a third independent reviewer or not. If the data is available, it could also be useful to note the actual level of agreement (i.e. how many of the papers did the two reviewers agree on) to support the robustness of this process.

- I note in the Abstract and PRISMA flowchart that papers reporting on participants over the age of 65 were excluded, however children of any ages (i.e. 0 onwards) were included. The reasons for these decisions don't seem to be explained either in the Introduction or the Inclusion/Exclusion criteria. It would be useful to be quite explicit about this choice, as I am not sure of the reasoning behind potentially including a 6 year old and not a 66 year old (for example).

The actual results and discussion sections appear reasonable, but I would like clarification on the above issues (particularly timeframes/review protocol and inclusion criteria) before I could be confident that the described processes are sufficiently robust.

**Reviewer**	**3**
**Name**	**Oliver, Stacey**
**Affiliation**	**Summer Foundation, Research and Innovation**
**Date**	**10-Mar-2026**
**COI**	**None**

Thank you for the opportunity to review this systematic review examining interventions to promote social inclusion for people with disabilities in high-income countries. The manuscript addresses an important and relevant topic. Overall, this is a well-conducted and clearly presented review, and the authors are to be commended for their work. This manuscript will be a useful resource for researchers and practitioners working in this area.

I have a few minor comments for consideration that may help further strengthen the manuscript.

General - The authors use the ecological pathways model described by Simplican et al. (2015) and the social pillar of the CBR matrix (WHO, 2010), further developed by Saran et al. (2021), to categorise the results. While these frameworks are clearly referenced, including brief definitions of the categories within the manuscript would improve readability. I note that these definitions are provided in the appendices; however, presenting a table or brief summary earlier in the paper would make the results easier for readers to interpret.

Results - Page 12, line 219: The manuscript notes that “seven non-RCTs” were included. It would be helpful to briefly specify the study designs used for these studies. I am also unclear why “before-and-after” and “quasi-experimental” studies are presented as separate categories when they could also be considered types of non-RCT study designs. The authors may wish to clarify or standardise the categorisation of study designs. For example, they could either specify all individual study designs or consistently classify studies as RCTs and non-RCTs. This categorisation may also need to be amended in Appendix 6.

Figure 1 - The Prisma flow diagram lists two exclusion categories:

1. No impact evaluation (n = 6)

2. No assessment of the impact of interventions on social inclusion outcomes for people with disabilities (n = 13)

It would be helpful for the authors to clarify these two exclusion categories and how they differ. Additionally, these categories are not listed section 2.1 ‘Study inclusion and exclusion criteria’.

Appendices 2 and 3 - These matrices are somewhat difficult to follow in their current format. The authors may wish to consider combining them with the previously suggested table that includes the framework category definitions. It may also be worth considering whether this information could be integrated with Appendix 7 to improve clarity and reduce duplication.

Appendix 6 - This contains important data from the literature review and may be better presented as a table within the main manuscript rather than in the appendix.

VERSION 1 - AUTHOR RESPONSE

Reviewer: 1
Dr. Anne Kumurenzi, University of Rwanda College of Medicine and Health Sciences
Comments to the Author:

Review for Effectiveness of Interventions to Promote Social Inclusion of People with Disabilities in High-Income Countries: A Systematic Review
General comments:
The manuscript presents a comprehensive study of “Effectiveness of Interventions to Promote Social Inclusion of People with Disabilities in High-Income Countries: A Systematic Review”, offering valuable insights into social inclusion interventions and outcomes across various high-income contexts. The research question is clearly defined, and the paper aims to address a gap in the literature on the effectiveness of social interventions. However, there are several areas in the methodology, results, and discussion sections that require revision for clarity, consistency, and scientific rigor.

Major revisions:

- Participants: The inclusion of both children and adults in the study raises questions about the comparability of the interventions and outcomes. It is unclear how the study accounted for potential differences in age, disability type, or intervention response across these two groups. The authors should either provide more details on how the data was disaggregated by age or consider justifying the inclusion of both groups in the same analysis.

We thank the reviewer for this important comment, which is closely related to a point also raised by the Associate Editor and that we feel we have now addressed consistently across both responses. We agree that child- and adult-focused interventions may differ in their design, targets, and outcomes. Our intention in including both age groups was to review the broad field of social inclusion interventions across the life course, using a common conceptual framework applicable across age groups.

To address the reviewer's concern, we have now added an explicit rationale in section 2.1 (page 7):

“The age range of 0–65 years was selected to capture social inclusion across childhood, youth, and working-age adulthood. The upper limit was applied to keep the review focused on disability-related social inclusion interventions and to avoid conflating these with issues more specific to ageing, retirement, and later-life care. Both children and adults were included because social inclusion is a life-course construct, and the review aimed to examine whether intervention patterns and effectiveness differ across age groups. No lower age limit was applied, as social exclusion associated with disability often begins in early childhood, and many interventions aiming to improve social inclusion are implemented in educational, family, and community settings.”

Furthermore, an age-disaggregated analysis has been added in a dedicated Results section (section 3.6, table 4, p.20-p.21), distinguishing child-only (n=21), adult-only (n=25), and mixed-age (n=4) studies, and summarising intervention and outcome patterns by age group. We have not undertaken further disaggregation by combined age and disability subgroup because the number of studies within many cells was too small to support meaningful interpretation. This limitation is acknowledged in the Discussion (p. 31), where we clarify that any cross-age comparisons are interpretive and not based on pooled quantitative synthesis, and that disability type and outcome domain are likely stronger predictors of effectiveness than age group per se.

- Interventions: The manuscript mentions that interventions may differ, but it remains unclear how the diversity of interventions and their outcomes were handled in the analysis. The authors should describe how these variations were treated in their synthesis. Were interventions analyzed separately for each age group, or were they combined?

We respectfully note that this was already largely addressed in section 2.5 of the original manuscript (page 12). Interventions were not pooled statistically; instead, they were classified using the combined ecological-CBR framework and synthesised narratively by intervention category, ecological level, outcome domain, and age group. The section explicitly states:

“Data were synthesised and structured by intervention category, ecological level, outcome domain, and age group, using the combined CBR-ecological framework. This grouping was chosen because the heterogeneity of intervention types, populations, and outcome measures precluded statistical pooling.”

Fisher's exact tests were additionally used to explore associations between intervention category, ecological level, and effectiveness.

To further improve visibility of this information, we have substantially revised section 2.5 (page 12) to make even more explicit that: (1) studies were grouped narratively by intervention category, ecological level, outcome domain, and age group rather than quantitatively pooled, given the heterogeneity of intervention types, populations, and outcome measures; (2) the synthesis method used was vote counting based on direction of effect, following the SWiM reporting guideline ; and (3) age-group-specific patterns are described in a dedicated section (section 3.6), while acknowledging that formal subgroup comparison was not feasible given the small number of studies within many subgroup cells.

Exclusion: The exclusion of studies not conducted in high-income countries is mentioned, but this is obvious since the review focused on high-income countries.

We agree that this may appear self-evident; however, we have chosen to retain this criterion explicitly in the manuscript to ensure full transparency and reproducibility of the review process and to allow readers to fully assess the scope of the evidence base., in line with standard systematic review practice (PRISMA reporting guidelines).

- Results: The results section lacks clarity. For instance, the statement "Fifteen studies reported significant positive effects, 12 no effect" is vague. What specific outcomes were these studies assessing? What criteria were used to define "positive" or "no effect"? The authors should provide more detailed descriptions of the outcomes assessed, as well as the specific interventions that led to positive or no effects. A table summarizing the effects of different interventions would enhance the clarity of this section.

We agree that the Results section required greater clarity. We have now addressed this in three ways.

First, the effect classification criteria have been made explicit in the Methods section (section 2.5, page 13):

"The standardised metric was the direction of effect based on statistical significance as reported by study authors. Where available, effect sizes, p-values, and confidence intervals were also extracted. Studies were classified as 'positive', if all social inclusion outcomes reached statistical significance in the expected direction; as 'no effect' if none did; as 'mixed' if some outcomes were significant while others were not; or as 'negative', if all outcomes showed a statistically significant effect in the opposite direction. Mixed-effect studies were subsequently disaggregated to the outcome level, with each individual outcome reclassified as positive, no effect, or negative."

These definitions are also restated at the opening of the Results section (section 3.5, page 19): *"Effectiveness was categorised as positive (all outcomes statistically significant in the expected direction), no effect (no outcomes statistically significant), or mixed (some outcomes significant, others not), based on statistical significance as reported by study authors."*

Second, we have added a compact summary table in the main text (Table 3, page 20) presenting for each of the 50 included studies the population, intervention category (CBR domain and ecological level), intervention description, outcome category, specific outcome assessed, and key statistics including p-values and effect sizes where available. Studies are ordered by effectiveness (positive, no effect, mixed).

We initially included this as Table 3 in the main manuscript; however, given the scope of the review (50 included studies), a sufficiently detailed summary table inevitably exceeds the journal's formatting requirements of a maximum of two pages and nine columns. Following editorial guidance, the table has therefore been moved to the appendices. All in-text citations have been updated accordingly.

Third, the Results text (section 3.5, pages 19-20) has been revised so that summary numbers are immediately linked to the most commonly assessed outcomes and intervention categories.

- Narrative synthesis and statistical analysis: The results are narratively synthesized, but there is limited mention of statistical analysis or how the studies were quantitatively assessed (mainly in the abstract). For the main documents, if available, the authors should include statistical analyses, such as effect sizes, confidence intervals, or p-values, to provide more robust evidence of the effectiveness of the interventions.

We respectfully agree only in part. Where individual studies reported p-values or confidence intervals, these have been included selectively in the summary table and the text. However, a pooled quantitative synthesis was not feasible because the studies were highly heterogeneous in design, population, intervention content, outcome constructs, and reporting metrics. Many studies did not report effect sizes in a way that permitted direct comparison. We have addressed this comment in three ways.

First, the rationale for not conducting a meta-analysis is explicitly stated in section 2.5 (page 12), where we also describe how all data were handled analytically:

"Meta-analysis and effect-size pooling were not appropriate for this review, as the included studies differed considerably in study design, intervention type, outcome constructs, and reporting metrics, seriously compromising direct statistical comparison of effect estimates. The synthesis method used was therefore vote counting based on direction of effect, complemented by textual description and tabulation of study characteristics and findings, following the Synthesis Without Meta-analysis (SWiM) reporting guideline [49]. The standardised metric was the direction of effect based on statistical significance as reported by study authors. Where available, effect sizes, p-values, and confidence intervals were also extracted."

Second, the Fisher's exact tests used to explore broad patterns across categories are described and justified in section 2.5 (page 13):

"Fisher's exact tests were used to explore associations between effectiveness and intervention category, ecological level, and outcome category, as well as between effectiveness and risk of bias. These tests were selected due to small sample sizes and low expected cell counts in several subgroups, which limited the applicability of chi-squared or other parametric tests.' Results of these tests are reported in section 3.5 (pages 19-20): 'No significant association was found between intervention category and effectiveness ($p = 0.278$), and between effectiveness and ecological level ($p = 0.947$)' and 'Statistical analysis using Fisher's Exact Test found a significant association between outcome category and effectiveness ($p = 0.017$), but no significant association between ecological level and effectiveness ($p = 0.358$)."

Third, study-level statistical details including p-values, confidence intervals, and effect sizes where available have been added to the compact summary table (Table 3, page 20). This table presents for each of the 50 included studies the population, intervention category, outcome category, specific outcome, and key statistics, making the statistical evidence as transparent as possible given the heterogeneity of the evidence base.

Minor revisions:

- Methodology: Some sections of the methods, particularly the search strategy, contain inconsistencies. For example, the abstract mentions eight databases, but the methods section mentions only seven. Additionally, the dates covered in the search are not consistent in the abstract and main document.
  Thanks for pointing out these inconsistencies and errors! They have now been corrected throughout the manuscript. The abstract and methods section now consistently refer to seven databases. The incorrect date limit mentioned in the original abstract has been removed, and the manuscript now consistently states that no limits or filters were applied in terms of language or publication date (page 9).

- Bias assessment: The paper mentions a "risk of bias" section but does not provide sufficient detail on how the risk of bias was assessed for each included study. The authors should elaborate on the tools or criteria used for bias assessment.
  We agree that the original manuscript lacked sufficient detail on the risk of bias assessment. The methods section on risk of bias assessment (section 2.3, pages 10-11) has now been substantially expanded to provide full details on the tools used (ROB2 and ROBINS-I), the domains assessed by each tool, the rating categories applied, the adaptations made for single-

group before-after studies, the piloting process, the application and blinding procedure, and the use of Fisher's exact tests to explore the association between risk of bias and reported effectiveness.

- Improve the table in the appendix with the bias ratings for each study would improve the transparency of the review process.
  We agree that transparent reporting of risk-of-bias assessments is important. The appendix already provides study-level domain judgements and overall ratings for both ROBINS-I and RoB2 (Appendix 10), following the colour-coded presentation format recommended by Cochrane [Moore et al., 2023; https://www.cochrane.org/sites/default/files/uploads/PDFs/Final_ROBINS-I%20author%20guidance_0.pdf]. As stated in the Cochrane ROBINS-I guidance, these tools are based on categorical domain-level judgements rather than additive numeric scores, and the overall judgement should be based on an assessment of all domain-level biases. For this reason, we would prefer to retain the domain-level and overall ratings as the most appropriate and interpretable form of presentation, in line with standard Cochrane guidance. The appendix is referenced explicitly in the Results section (section 3.7): 'The detailed study-level risk-of-bias assessments, including domain-specific and overall judgements for each included study, are presented in Appendix 10.

  There are several grammatical and structural issues, the authors should carefully proofread the manuscript for grammar and structure.

  The manuscript has now been carefully proofread for grammar, spelling, and structural consistency. In particular, inconsistencies in spelling (e.g. US versus UK English), formatting, and reference style have been corrected throughout.

- Discussion:
  Interpretation of results: While the results are synthesized, the discussion does not sufficiently address the implications of the findings or how they relate to existing literature.
  The rationale behind the findings should be more thoroughly explored. The authors should explore deeper into the possible reasons for the lack of consistent outcomes across studies.
  They should also discuss the implications for practice more explicitly, suggesting how the findings can inform future interventions.

We thank the reviewer for this important comment. We agree that the original Discussion synthesised the findings but did not always make the interpretation sufficiently explicit. We have therefore very considerably revised the Discussion to address this concern in three ways.

First, we have added a dedicated paragraph (p.22) explicitly synthesising the likely reasons for heterogeneous findings across studies, including: the high heterogeneity of interventions in content, intensity, duration and target population; the greater difficulty of changing distal participation outcomes compared with more proximal skill-based outcomes; the dependence of community participation on environmental accessibility, social attitudes and structural supports that individual-level interventions cannot address alone; short follow-up periods insufficient to capture sustained change; and variation in outcome measurement that may have missed subjective or longer-term dimensions of inclusion.

Second, we have strengthened the connections to existing literature throughout the Discussion (pp. 22–27). For example, we now situate our findings in relation to prior systematic reviews and

empirical studies - including, among others, Killaspy et al. [104] on the particular complexity of social interventions for people with severe mental illness, and Eager et al. [105], whose qualitative study identified stigma as a barrier operating across almost every aspect of life for this population.

Third, we have made the implications for practice more explicit in the revised Policy Implications section (p.31), which now clearly states that interventions should be designed at the same ecological level as the outcome they seek to change, and that community participation outcomes require multi-level approaches combining individual support with environmental and structural measures.

To keep the manuscript concise, we have simultaneously streamlined the Policy Implications and Future Research sections (p.31-33) by removing content that was repetitive or too general, and we have made the Conclusion more concise.

- Some sentences in the discussion are bolded, but the reason for this emphasis is unclear.

The bold formatting has been removed from the Discussion section.

Conclusion:
In conclusion, while the manuscript addresses a significant topic and has the potential to contribute meaningfully to the field, it requires revisions to improve clarity it. The study would benefit from clearer distinctions between age groups, a more transparent explanation of the data synthesis methods, and a stronger discussion of the results. I recommend a thorough revision to address the concerns raised above.

We appreciate the constructive comments by the reviewer! As detailed above, we have now substantially revised the manuscript to address each of these points.

Reviewer: 2
Dr. Stuart Wark, University of New England
Comments to the Author:

This manuscript reports on a systematic review of the research quantum regarding the effectiveness of interventions that support the better inclusion of people with disabilities. The demographic focus of the review is on high-income countries. I believe that the chosen databases are appropriate for the question, and the risk of bias assessed in a suitable manner.

- There does seem to be some inconsistency in the usage of US and UK spelling (i.e. words with 'ize' like synthesizes versus words with "iour" like behaviour) but this is easily remedied.

The manuscript has now been re-checked thoroughly to ensure consistent use of UK English spelling.

The authors are to be commended for their consideration of this topic area, as it is not normally explored within more mainstream health journals.

I do have some initial observations and comments for the authors to consider, and these are detailed below:

- I was intrigued that the authors decided to exclude all qualitative papers from the review. This is potentially appropriate if the plan is to do a meta-analysis (for example), but I wasn't entire sure of the rationale for excluding personal testimony and/or feedback from the primary participants/beneficiaries of any intervention? I recognise the conceptual desire for only including evaluation studies, but does that approach necessarily exclude all qualitative papers? This is not a major issue, and a brief explanation as to why this decision was made would assist the reader to understand the context.

We agree that the rationale should be clearer. The review was designed specifically to evaluate intervention effectiveness, which required pre/post or between-group outcome assessment. Qualitative studies provide valuable insight into lived experience, implementation, and acceptability, but they do not usually permit the same kind of effectiveness classification used here. We have now clarified this rationale and acknowledge that excluding qualitative research limits insight into mechanisms and participant perspectives.

A brief justification has been added to the eligibility criteria section (section 2.1, page 7):
"*Qualitative studies were excluded, because the review focused on quantitatively measured changes in social inclusion outcomes, in order to evaluate the effectiveness of interventions. While qualitative research provides valuable insights into participants' lived experiences and perspectives, it does not yield the outcome measures needed to assess whether interventions produce measurable change."*

This limitation is also acknowledged in the Discussion (page 30): "*The exclusion of qualitative studies, while appropriate for an effectiveness review requiring quantitative outcome data, means that important insights into participants' lived experiences, implementation processes, and intervention mechanisms are not captured in this synthesis. A complementary review incorporating qualitative evidence would be valuable to provide a fuller understanding of how and why social inclusion interventions work."*

- The actual search was undertaken nearly two years ago (manuscript submitted to journal in late January 2026). I understand that review papers take a lot of time to develop, but this timeframe is now significant. Is there likely to be any more recent research that should be included? This is highly relevant as most of the papers identified occurred within the most recent time period (18 papers from 2020-24) and therefore it seems probable that more papers will have emerged that could add substantially to the review.

We agree. Given the elapsed time since the original search, we have now updated the search to March 2026 and we have revised the PRISMA flow diagram, study count, and relevant sections accordingly.

- The Abstract notes eight databases being searched, but then only lists seven. The Methods nominates seven, so I assume that the 'eight' is just a typo?

Apologies – this was our fault! The abstract and methods section now consistently refer to seven databases throughout the manuscript.

- The choice to not have at least two reviewers independently evaluate all papers at both the title/abstract screen stage and then definitely at the full-text stage is always slightly concerning to me. Again, I understand the rationale for this choice due to time efficiency, but this does come at the increased risk of errors. It would be good, at a minimum, to confirm that the second reviewer was truly independent (i.e. they were unaware of other reviewer's decisions) at each stage of their sample screening, whether all the full-text exclusions were verified by the second reviewer

We agree that the screening process should be reported more transparently. We have now clarified all aspects raised in section 2.4 (pages 10-11).

First, regarding reviewer independence, Covidence's built-in blinding functionality ensured full independence: (p.11) "*At all stages of screening, Covidence's built-in blinding functionality ensured that each reviewer's decisions remained concealed from the other until both had completed their independent assessments, preventing any influence between reviewers."*

Second, regarding the proportion of full-text papers independently reviewed and inter-rater agreement *(p.11): "Subsequently, the first author evaluated 168 full-text papers against the pre-established inclusion and exclusion criteria, of which 50% were randomly selected for independent review by co-author MSu, resulting in 50 articles for the final analysis. Inter-rater agreement was 83% at title and abstract screening stage and 82% at full-text screening stage, indicating substantial agreement."*

Third, regarding disagreement resolution (p.10): "*Disagreements between reviewers at any stage of the review process were resolved through discussion between both researchers."*

No third reviewer was involved.

Finally, regarding the risk of bias assessment, the same independent and blinded process was applied: "*One researcher (MSc) applied the assessment to all included studies, complemented by an independent double-review by another researcher (MSu) on a randomised 25% of the studies. Independent assessments were conducted using the Covidence systematic literature tool, which ensured that reviewers were blinded to each other's ratings. Any disagreements were resolved through discussion' (page 10)."*

- I note in the Abstract and PRISMA flowchart that papers reporting on participants over the age of 65 were excluded, however children of any ages (i.e. 0 onwards) were included. The reasons for these decisions don't seem to be explained either in the Introduction or the Inclusion/Exclusion criteria. It would be useful to be quite explicit about this choice, as I am not sure of the reasoning behind potentially including a 6 year old and not a 66 year old (for example).

We agree and have now made this explicit. The intention was to focus the review on childhood through working-age adulthood while avoiding conflation with later-life interventions that may be shaped by ageing-specific pathways. We acknowledge that this boundary is somewhat arbitrary. The following rationale has been added to section 2.1 (page 7):

"The age range of 0–65 years was selected to capture social inclusion across childhood, youth, and working-age adulthood. The upper limit was applied to keep the review focused on disability-related social inclusion interventions and to avoid conflating these with issues more specific to ageing, retirement, and later-life care, as participation in later life is commonly examined within distinct research traditions concerned with age-related health and long-term care (e.g., [39–41]). Both children and adults were included because social inclusion is a life-course construct, and the review aimed to examine whether intervention patterns and effectiveness differ across age groups. No lower age limit was applied, as social exclusion associated with disability often begins in early childhood, and many interventions aiming to improve social inclusion are implemented in educational, family, and community settings. In addition, several studies in this field include both children and adults within the same intervention, such that excluding children would have led to the omission of relevant evidence."

The actual results and discussion sections appear reasonable, but I would like clarification on the above issues (particularly timeframes/review protocol and inclusion criteria) before I could be confident that the described processes are sufficiently robust.

We appreciate the constructive feedback by the reviewer. As outlined above, we have significantly revised the manuscript now in order to address these points.

Reviewer: 3

Dr. Stacey Oliver, Summer Foundation, La Trobe University

Comments to the Author:

Thank you for the opportunity to review this systematic review examining interventions to promote social inclusion for people with disabilities in high-income countries. The manuscript addresses an important and relevant topic. Overall, this is a well-conducted and clearly presented review, and the authors are to be commended for their work. This manuscript will be a useful resource for researchers and practitioners working in this area.

I have a few minor comments for consideration that may help further strengthen the manuscript.

- General - The authors use the ecological pathways model described by Simplican et al. (2015) and the social pillar of the CBR matrix (WHO, 2010), further developed by Saran et al. (2021), to categorise the results. While these frameworks are clearly referenced, including brief definitions of the categories within the manuscript would improve readability. I note that these definitions are provided in the appendices; however, presenting a table or brief summary earlier in the paper would make the results easier for readers to interpret.

We agree. The appendices already contain the full category detail, but readability is improved by bringing a concise summary into the main manuscript. We have now added two summary tables in the Methods section (Tables 1 and 2, page 8-9) presenting concise definitions of all categories from both frameworks — the ecological levels based on Simplican et al. (2015) and the CBR social-domain categories based on WHO (2010) further developed by Saran et al. (2021). The full classification matrices remain in Appendices 2 and 3 for reference.

- Results - Page 12, line 219: The manuscript notes that "seven non-RCTs" were included. It would be helpful to briefly specify the study designs used for these studies. I am also unclear why "before-and-after" and "quasi-experimental" studies are presented as separate categories when they could also be considered types of non-RCT study designs. The authors may wish to clarify or standardise the categorisation of study designs. For example, they could either specify all individual study designs or consistently classify studies as RCTs and non-RCTs. This categorisation may also need to be amended in Appendix 6.

We agree that the categorisation of study designs required clarification and standardisation. We have now standardised the terminology used for study designs across the manuscript, including Appendix 7. The included studies are now presented using three consistent categories: RCTs (n=27), non-randomised controlled trials (n=9), and single-group before-and-after studies (n=14).

A clarifying sentence has been added in the Results (section 3.1, page 14) to explain the distinction between the latter two categories:

"While the latter two categories are both non-randomised in nature, they are distinguished here by the presence or absence of a control group: non-RCTs included a comparison group without random allocation, while before-and-after studies measured outcomes in a single group without a control group."

The quasi-experimental category has been removed from the Results, as the studies previously classified as such were more accurately described as non-randomised controlled trials. We have retained quasi-experimental designs in the eligibility criteria (section 2.1, page 6) because they were eligible for inclusion in principle, although no included study was ultimately reported under this category. Appendix 7 has been updated accordingly.

- Figure 1 - The Prisma flow diagram lists two exclusion categories:
  1. No impact evaluation (n = 6)
  2. No assessment of the impact of interventions on social inclusion outcomes for people with disabilities (n = 13)

  It would be helpful for the authors to clarify these two exclusion categories and how they differ. Additionally, these categories are not listed section 2.1 'Study inclusion and exclusion criteria'.

We thank the reviewer for this observation. We agree that the two exclusion categories were not distinct and could cause confusion. We have merged them in the PRISMA flow diagram into a single category '"no impact evaluation of social inclusion outcomes for people with disabilities"(n=19) (Figure 1) as both groups of studies ultimately failed on the same inclusion criterion. A corresponding exclusion criterion has been added to section 2.1 (page 6) and to Appendix 1 to ensure consistency between the flow diagram and the eligibility criteria:

"if they did not include an impact evaluation of an intervention or if the impact evaluation did not assess outcomes related to social inclusion for people with disabilities"

- Appendices 2 and 3 - These matrices are somewhat difficult to follow in their current format. The authors may wish to consider combining them with the previously suggested table that includes the framework category definitions. It may also be worth considering whether this information could be integrated with Appendix 7 to improve clarity and reduce duplication.

We acknowledge the reviewer's suggestion regarding the format of Appendices 2 and 3 and the concern about potential duplication. However, each appendix serves a distinct purpose and addresses a different need of the reader: Tables 1 and 2 (added to the Methods section, pages 8-9) provide a compact summary of both frameworks to improve readability within the manuscript itself; Appendices 2 and 3 present the full classification framework with detailed category definitions; and Appendices 8 and 9 present the actual classification of included studies within that framework. We believe this three-level structure adds clarity rather than redundancy, and that merging these elements would risk making individual components harder to navigate. We would therefore propose to retain the current structure.

- Appendix 6 - This contains important data from the literature review and may be better presented as a table within the main manuscript rather than in the appendix.

We thank the reviewer for this suggestion, which aligns with a similar comment raised by Reviewer 1. In response to both reviewers, we have now added a compact summary table (Table 3, page 20) in the main manuscript, presenting, for each of the 50 included studies, the population, intervention category, intervention description, outcome category, specific outcome assessed,

and key statistics including p-values and effect sizes where available. Studies are ordered by effectiveness (positive, no effect, mixed). The full detailed extraction table remains in Appendix 7 for readers who wish to consult the complete study-level data.

We initially included this as Table 3 in the main manuscript; however, given the scope of the review (50 included studies), a sufficiently detailed summary table inevitably exceeds the journal's formatting requirements of a maximum of two pages and nine columns. Following editorial guidance, the table has therefore been moved to the appendices. All in-text citations have been updated accordingly.

VERSION 2 - REVIEW

**Reviewer**	**2**
**Name**	**Wark, Stuart**
**Affiliation**	**University of New England, School of Rural Medicine**
**Date**	**05-May-2026**
**COI**	

I note that the authors have made significant changes in response to the feedback provided on the first version of this manuscript. I commend the authors for their willingness to respond positively to these comments, and believe that they have largely addressed the concerns as raised.
